# Cancer-Stem-Cell Phenotype-Guided Discovery of a Microbiota-Inspired Synthetic Compound Targeting NPM1 for Leukemia

**DOI:** 10.1021/jacsau.3c00682

**Published:** 2024-02-09

**Authors:** Sergio Algar, Henar Vázquez-Villa, Pedro Aguilar-Garrido, Miguel Ángel Navarro-Aguadero, María Velasco-Estévez, Anabel Sánchez-Merino, Iván Arribas-Álvarez, Alberto Paradela, Rafael L. Giner-Arroyo, Joaquín Tamargo-Azpilicueta, Irene Díaz-Moreno, Joaquín Martínez-López, Miguel Gallardo, María L. López-Rodríguez, Bellinda Benhamú

**Affiliations:** † Department of Organic Chemistry, Faculty of Chemistry, Universidad Complutense de Madrid, E-28040 Madrid, Spain; ‡ Department of Haematology, Hospital Universitario 12 de Octubre, Instituto de Investigación Sanitaria Hospital 12 de Octubre (imas12), E-28041 Madrid, Spain; § H12O-CNIO Haematological Malignancies Clinical Research Unit, Spanish National Cancer Research Centre, E-28029 Madrid, Spain; ∥ Proteomics Laboratory, CNB, CSIC, E-28049 Madrid, Spain; ⊥ Institute for Chemical Research, cicCartuja, University of Seville, CSIC, E-41092 Sevilla, Spain

**Keywords:** drug discovery, small molecule, microbiota, organocatalysis, stem cells, NPM1, leukemia

## Abstract

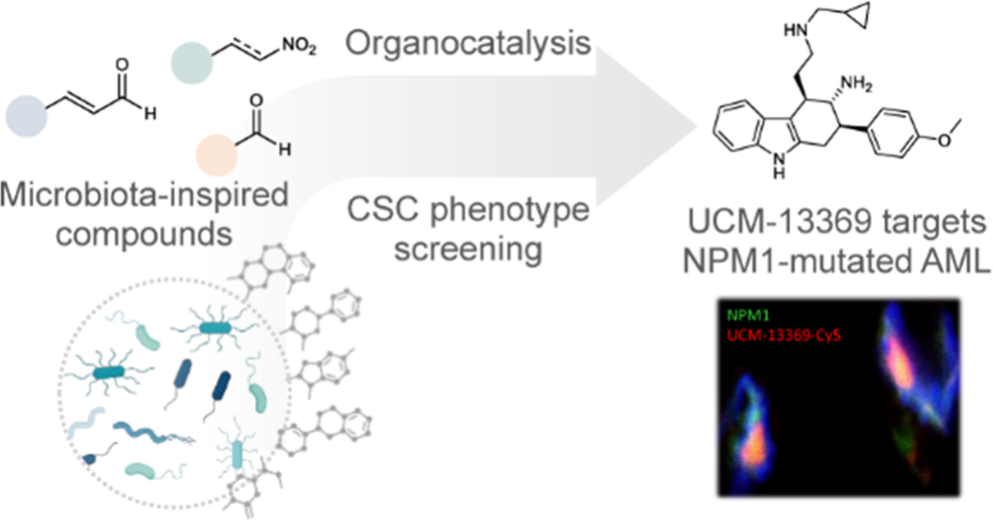

The human microbiota plays an important role in human health and disease, through the secretion of metabolites that regulate key biological functions. We propose that microbiota metabolites represent an unexplored chemical space of small drug-like molecules in the search of new hits for drug discovery. Here, we describe the generation of a set of complex chemotypes inspired on selected microbiota metabolites, which have been synthesized using asymmetric organocatalytic reactions. Following a primary screening in CSC models, we identified the novel compound UCM-13369 (**4b**) whose cytotoxicity was mediated by NPM1. This protein is one of the most frequent mutations of AML, and NPM1-mutated AML is recognized by the WHO as a distinct hematopoietic malignancy. UCM-13369 inhibits NPM1 expression, downregulates the pathway associated with mutant NPM1 C+, and specifically recognizes the C-end DNA-binding domain of NPM1 C+, avoiding the nucleus-cytoplasm translocation involved in the AML tumorological process. The new NPM1 inhibitor triggers apoptosis in AML cell lines and primary cells from AML patients and reduces tumor infiltration in a mouse model of AML with NPM1 C+ mutation. The disclosed phenotype-guided discovery of UCM-13369, a novel small molecule inspired on microbiota metabolites, confirms that CSC death induced by NPM1 inhibition represents a promising therapeutic opportunity for NPM1-mutated AML, a high-mortality disease.

## Introduction

The human microbiota is a complex ecosystem of symbiotic microorganisms that play an important role in human health and disease, in part through the secretion of metabolites that can regulate host proteins.^1,2^ Microbiota can synthesize, transform, and degrade a large repertoire of organic molecules that are key for maintaining homeostasis providing beneficial functions to the host. Consequently, microbiota dysbiosis, i.e., alterations in its composition that result from exposure to environmental factors, such as diet, xenobiotics, drugs, and pathogens, eventually contributes to the pathogenesis of various diseases, such as obesity, type 2 diabetes, cardiovascular disease, cancer, and neurological disorders.^3−6^ In this context, several metabolites produced by the microbiota have been shown to confer protection against disease due to their antioxidant and anti-inflammatory properties.^7−9^ For example, equol exhibits strong antiproliferative effects on breast neoplasm and hepatocellular carcinoma;^10,11^ urolithins have been suggested to target specific tumorigenesis pathways in prostate cancer;^12,13^ niacin (vitamin B3) has been shown to suppress colitis and colon cancer in mice;^14,15^ indole appears to modulate the important antihyperglycemic hormone glucagon-like peptide (GLP1), with a substantial impact on pancreatic function, insulin release, and food intake regulation;^16^ protocatechiuc acid prevents esophageal cancer;^17^ and butyrate has been reported to modulate the activity of the nervous system in mouse models.^18^ Hence, microbiota metabolism produces a wide and diverse range of bioactive small molecules that are available to the host to regulate key biological functions. The compatibility of the microbiome with healthy human systems suggests that microbiota metabolites represent drug-like molecules that are highly specific modulators with no risk of adverse effects.^8^ This supports the hypothesis that the metabolites can be considered an unexplored chemical space of small organic molecules in the search of initial hits toward the identification of novel drug candidates for the treatment of a wide range of pathological conditions. Focused on cancer, in the present work, we have designed and synthesized a set of new small molecules inspired on human microbiota metabolites and explored their potential in cancer cellular models with the ultimate goal to identify novel antitumor drug candidates. Following a primary screening in cancer stem cells (CSCs), we have identified the synthetic compound UCM-13369 that has been subsequently characterized as a nucleophosmin 1 (NPM1) inhibitor that specifically recognizes the C-end DNA-binding domain of the protein. The capacity of the new NPM1 inhibitor to trigger stem cell apoptosis has been translated to *ex vivo* and *in vivo* efficacy in models of acute myeloid leukemia (AML).^19^ Our results support phenotypic drug discovery as a valuable approach to develop active molecules in a pathologically relevant model, which together with the validation of the target protein involved in the disease, contributes to enhance innovation and deliver truly novel therapeutics for unmet medical needs.^20,21^

## Results

### Design and Synthesis of New Microbiota-Inspired Compounds

**Figure 1:**
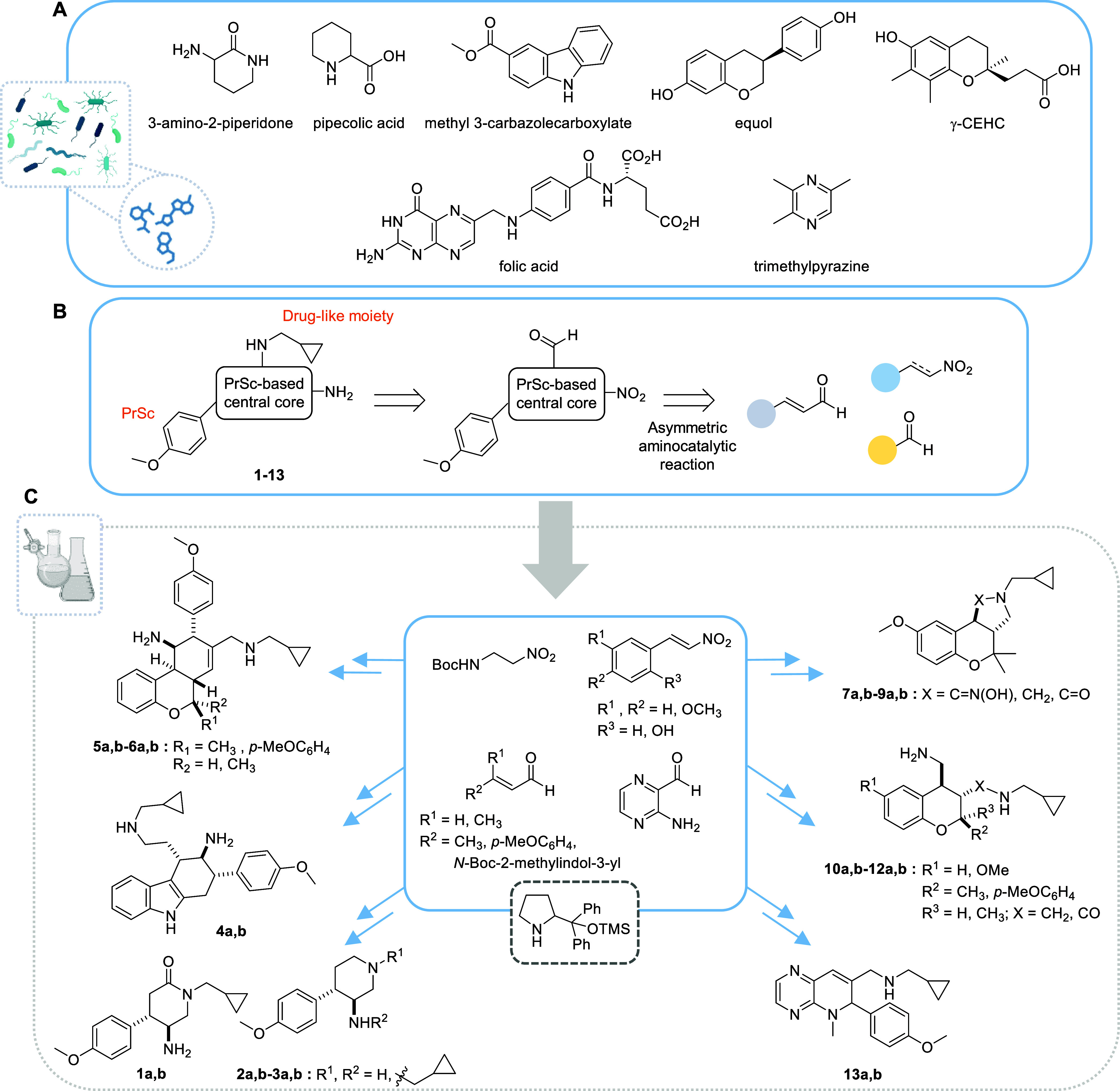
Design of new chemotypes inspired on human microbiota metabolites. (A) Selected small-molecule metabolites produced by the microbiota. (B) Synthetic approach via asymmetric aminocatalysis to generate microbiota-inspired molecules **1**–**13**. (C) Overview of the synthetic strategy based on a reaction between an α,β-unsaturated aldehyde and a nitro compound or a second aldehyde. PrSc: privileged scaffold. Untapered lines represent the relative stereochemistry; a and b are used to designate the two enantiomers of each compound.

We propose that microbiota metabolites represent an interesting starting point for the design of new compounds for drug discovery. Toward this end, we have analyzed the structures of identified small-molecule metabolites^22^ and explored the repertoire of organocatalytic reactions, to generate a set of microbiota-inspired compounds that combine metabolite structural diversity with synthetic accessibility using a key methodology that allows to obtain chemotypes with high structural complexity in a straightforward and stereocontrolled manner. This strategy offers the advantage of combining two or more reagents in a one-pot fashion to effectively yield a target chemotype. Thus, based on privileged scaffolds (PrSc) found in the metabolites selected in Figure 1A and amino catalytic reactions starting with a small group of aldehydes and nitro derivatives (Figure 1B), we designed new chemotypes that contain a PrSc-based central core linked to the common PrSc *p*-methoxyphenyl as well as to a drug-like moiety that could favor good pharmacokinetic properties. In the designed molecules **1**–**13** (Figure 1C), the central cores are piperidin-2-one, piperidine, tetrahydro-1*H*-carbazole, chromane, and dihydropyrido[2,3-*b*]pyrazine. These systems are inspired on metabolites including 3-amino-2-piperidone, pipecolic acid, methyl 3-carbazolecarboxylate, equol, γ-carboxyethylhydroxychroman (γ-CEHC) related to vitamin E, folic acid, and trimethylpyrazine shown in Figure 1A. The central cores are constructed in a single step and with high diastereoselectivities by reaction between an α,β-unsaturated aldehyde and a nitro compound or a second aldehyde promoted by diphenylprolinol trimethylsilyl ether catalyst (Figure 1C). Further transformation of aldehyde and/or nitro groups following a general sequence allows us to obtain the desired compounds **1**–**13** that contain the drug-like moiety (cyclopropylmethyl)amine and an amino group. For each chemotype, we have synthesized both enantiomers—designated as compounds **a** and **b**—with high enantioselectivity by using the corresponding catalyst.

**Figure 2:**
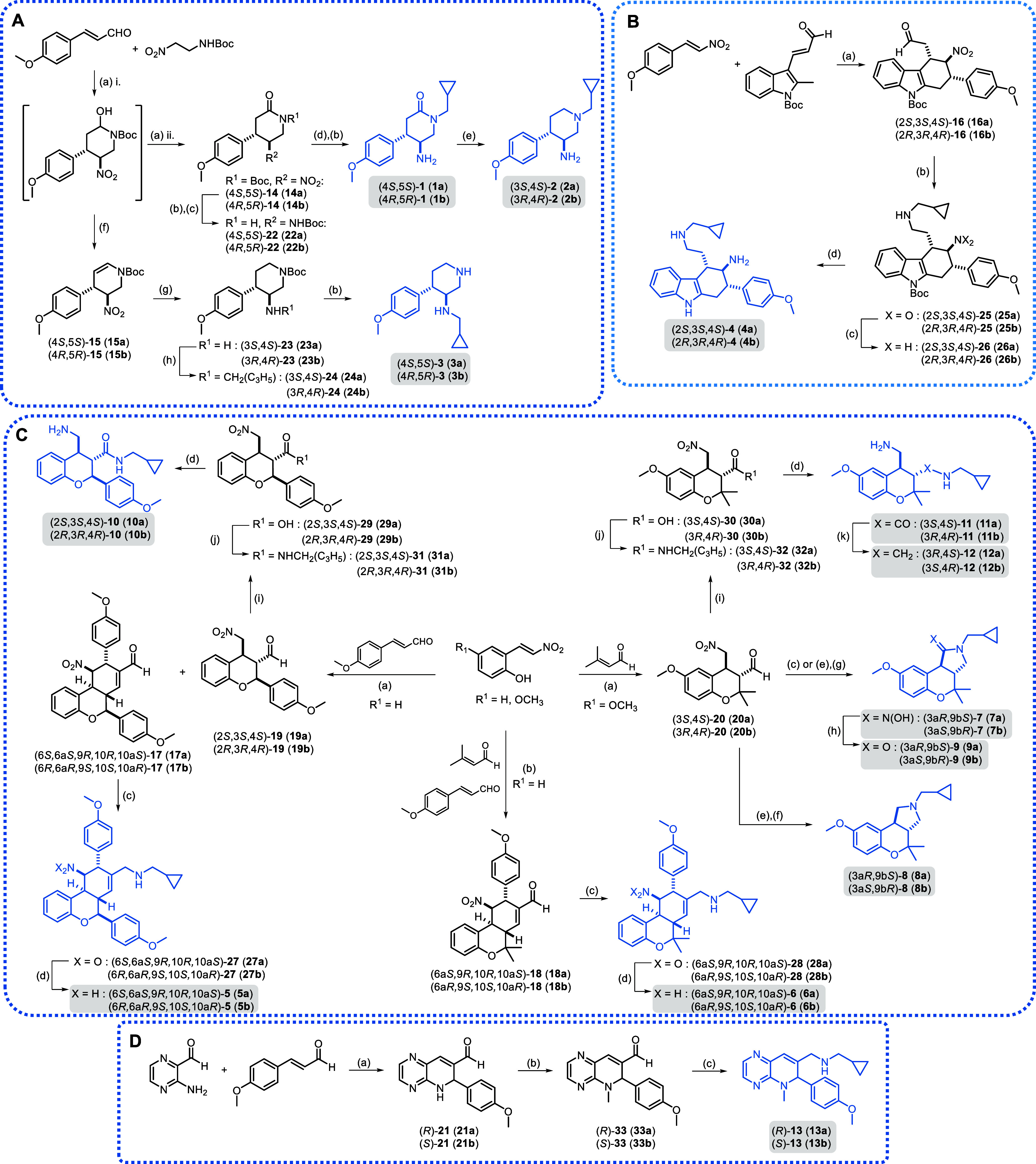
(A) Synthesis of compounds **1**–**3** containing 2-piperidinone or piperidine scaffolds. Reagents and conditions: (a) (i) 10 mol % chiral cat., 20 mol % benzoic acid, DCM, 0 °C to rt, 5 h; (ii) PDC, rt, on, 45–58%; (b) TFA, DCM, rt, 45 min-4 h, 73–93%; (c) (i) NiCl_2_·6H_2_O, NaBH_4_, MeOH, 0 °C, 1 h; (ii) Boc_2_O, rt, on, quantitative; (d) (i) NaH, DMF, 0 °C, 1 h; (ii) (bromomethyl)cyclopropane, NaI, rt, on, 36–42%; (e) LiAlH_4_, THF, rt, on, 77–85%; (f) TFA, rt, 2 h, 84–87%; (g) NiCl_2_·6H_2_O, NaBH_4_, MeOH, 0 °C to rt, on, 53–60%; (h) (i) cyclopropranecarbaldehyde, MeOH, rt, 4 h; (ii) NaBH_4_, rt, 2 h, 63–64%. (B) Synthesis of compound **4** containing a tetrahydrocarbazole scaffold. Reagents and conditions: (a) 20 mol % chiral cat., 20 mol % benzoic acid, toluene, MW, 110 °C, 5.5 h, 78%; (b) (i) (cyclopropylmethyl)amine, MeOH/DCM, rt, 3 h; (ii) NaBH_4_, rt, 2 h, 83–86%; (c) Zn, AcOH/MeOH, 2 h, quantitative; (d) HCl, MeOH, rt, 4 h, 40–50%. (C) Synthesis of compounds **5**–**12** containing chromane and tetrahydrobenzo[*c*]chromene scaffolds. Reagents and conditions: (a) 20 mol % chiral cat., 20 mol % AcOH, CHCl_3_, rt, on, 21–26% for **17**, 22–31% for **19**, 52–61% for **20**; (b) 20 mol % chiral cat., 20 mol % AcOH, CHCl_3_, rt, 1 h then 24 h, 43–48%; (c) (i) (cyclopropylmethyl)amine, MeOH/DCM, rt, 3 h; (ii) NaBH_4_, rt, 2 h, 17%-quantitative; (d) Zn, AcOH/MeOH, rt, 2 h, 41–93%; (e) (cyclopropylmethyl)amine, DCM, rt, 3 h, quantitative; (f) NaBH_4_, MeOH, rt, 3 h, 30–33%; (g) HSiCl_3_, DCM, DMF, rt, on, 80–89%; (h) NaNO_2_, AcOH, MeOH/H_2_O, rt, on, 30–40%; (i) NaClO_2_, KH_2_PO_4_, 2-methylbut-2-ene, *t*-BuOH/THF, 30 °C, on, 79%-quantitative; (j) (i) (COCl)_2_ (2 M in DCM), cat. DMF, DCM, rt, 1 h; (ii) (cyclopropylmethyl)amine, 0 °C to rt, 3–18 h, 55%-quantitative; (k) LiAlH_4_, THF, reflux, 24 h, 20–22%. (D) Synthesis of compound **13** containing a dihydropyrido[2,3-*b*]pyrazine scaffold. Reagents and conditions: (a) 20 mol % chiral cat., 20% mol AcOH, CHCl_3_, rt, 7 days, 40–45%; (b) CH_3_I, Cs_2_CO_3_, DMF, rt, 1 h, 95%-quantitative; (c) (i) (cyclopropylmethyl)amine, MeOH/DCM, rt, 3 h; (ii) NaBH_4_, 0 °C to rt, 1–2 h, 49–50%.

The synthesis of final compounds **1**–**3** (Figure 2A) containing a disubstituted piperidin-2-one or a piperidine scaffold started with the reaction between *N*-Boc-2-amino-1-nitroethane and *p*-methoxycinnamaldehyde to obtain intermediates **14** and **15**.^23^ Michael addition catalyzed by the *S* or *R* enantiomer of 2-{diphenyl[(trimethylsilyl)oxy]methyl}pyrrolidine in the presence of benzoic acid, and subsequent *in situ* oxidation of the resulting hemiaminal afforded piperidin-2-one scaffold **14** (diastereoisomeric ratio (dr) = 9:1, determined by ^1^H-nuclear magnetic resonance (NMR) analysis of the crude). Intermediate **14** was then deprotected before one-pot reduction of the nitro group and protection of the resulting amine to give *N*-Boc-5-aminopiperidin-2-one (**22**), which was alkylated at the amide nitrogen with (bromomethyl)cyclopropane and treated with trifluoroacetic acid (TFA) to obtain compound **1a** or **1b** (enantiomeric ratio (er) = 97:3 for **1a**, 96:4 for **1b**). Reduction of the carbonyl group of **1** yielded target compound **2a** or **2b** (er = 99:1). On the other hand, piperidine scaffold **15** (Figure 2A) was prepared via the same asymmetric organocatalytic Michael addition to *p*-methoxycinnamaldehyde, but this time the resulting cyclic hemiaminal was *in situ* dehydrated (dr = 8:2, determined by high-performance liquid chromatography–mass spectrometry (HPLC-MS) analysis of the crude). Subsequent reduction of both the nitro group and the double bond yielded 5-aminopiperidine **23**, which afforded final compound **3a** or **3b** via reductive amination and deprotection (er = 98:2).

For the synthesis of the tetrahydrocarbazole core present in compound **4** (Figure 2B), we applied an asymmetric Diels–Alder reaction between a β-indolyl α,β-unsaturated aldehyde and *trans-p-*methoxy-β-nitrostyrene catalyzed by the *S* or *R* form of the diphenylprolinol silyl ether.^24^ In this case, we optimized the reported thermal Diels–Alder reaction by using microwave (MW) conditions (irradiation at 110 °C for 5.5 h) to obtain cycloadduct **16** (dr = 98:2, ^1^H NMR; er = 96:4 for **16a**, 98:2 for **16b**). These new conditions increased the yield and reduced considerably the time with respect to the thermal reaction without affecting the stereoselectivity outcome. Compound **16** was further derivatized via reductive amination to introduce a (cyclopropylmethyl)amino moiety, subsequent reduction of the nitro group and Boc deprotection, to afford final compound **4a** or **4b** (er = 94:6 for **4a**, 95:5 for **4b**).

The chromane and tetrahydrobenzo[*c*]chromene scaffolds **17**–**20** (Figure 2C), precursors of compounds **5**–**12**, were synthesized via an asymmetric organocatalytic domino reaction between an *o*-nitrovinylphenol and a properly substituted α,β-unsaturated aldehyde.^25,26^ Thus, intermediates **17** and **19** were both obtained using *p*-methoxycinnamaldehyde and *o*-nitrovinylphenol through a domino *oxa*-Michael–Michael reaction and a quadruple cascade *oxa*-Michael–Michael–Michael-aldol condensation reaction, respectively. Upon optimization of the reported conditions, tetrahydrobenzo[*c*]chromene **17** (single diastereoisomer) and chromane **19** (dr 8:2, ^1^H NMR) were obtained in a 1:1 ratio. Regarding tetrahydrobenzo[*c*]chromene and chromane intermediates **18** and **20** (Figure 2C), bearing a geminal dimethyl group, the organocatalytic cascade between 3,3-dimethylacrolein and *trans*-2-hydroxy-5-methoxy-β-nitrostyrene afforded only the chromane derivative **20** (dr = 8:2, ^1^H NMR), through a domino *oxa*-Michael–Michael reaction. In this case, the corresponding tetrahydrobenzo[*c*]chromene scaffold was not formed, probably due to the steric hindrance of the geminal dimethyl group, which may prevent the second Michael addition to a third molecule of 3,3-dimethylacrolein. Tetrahydrobenzo[*c*]chromene **18** was then obtained following a two-step procedure. Initial reaction between *trans*-2-hydroxy-β-nitrostyrene and 3,3-dimethylacrolein resulted in a chromane intermediate, which was treated *in situ* with less hindered Michael acceptor *p*-methoxycinnamaldehyde to induce a subsequent Michael-aldol condensation that provided **18** as a single diastereoisomer. Next, reductive amination with (cyclopropylmethyl)amine followed by nitro reduction of tetrahydrobenzo[*c*]chromenes **17** and **18** provided final compounds **5a** or **5b** and **6a** or **6b**, respectively (er = 99:1 for **5a**, 98:2 for **5b**, 99:1 for **6a,b**) (Figure 2C). However, when the one-pot reductive amination was applied to chromane intermediate **20**, the expected amine was not formed, but oxime **7** was obtained instead. The procedure in two steps did not yield the expected product either but the cyclic amine **8** resulting from the reduction of the corresponding imine. Alternative reduction of the imine with trichlorosilane combined with *N*,*N*-dimethylformamide (DMF) as a soft Lewis base afforded again compound **7**, which was hydrolyzed to **9**. Unexpected chemotypes **7a,b-9a,b** (Figure 2C, er = 99:1 for **7a,b**, 96:4 for **8a,b**, 98:2 for **9a,b**) were considered as additional final compounds to be tested in the phenotypic assays. The synthesis of desired compounds **10**–**12** was achieved following an alternative synthetic route starting from chromanes **19** and **20** (Figure 2C). The aldehyde group was oxidized to the corresponding carboxylic acid through the Pinnick reaction, and the (cyclopropylmethyl)amine moiety was then incorporated by coupling via the acid chloride. Next, reduction of the nitro group under standard conditions afforded compounds **10a** or **10b** and **11a** or **11b**, respectively (er = 99:1 for **10a,b** and **11a,b**). Finally, the amide group in the latter was reduced to obtain analogue **12a** or **12b** (er = 99:1).

The synthesis of the dihydropyrido[2,3-*b*]pyrazine scaffold **21** (Figure 2D) was performed via an asymmetric domino *aza*-Michael-aldol reaction between 3-aminopyrazine-2-carbaldehyde and *p*-methoxycinnamaldehyde. *N*-Methylation followed by reductive amination with (cyclopropylmethyl)amine led to the final compound **13a** or **13b** (er = 98:2).

### Cancer-Stem-Cell (CSC) Phenotype Screening: Identification of UCM-13369

Once microbiota-inspired compounds **1a**–**13a** and **1b**–**13b** were synthesized, we carried out a cellular phenotypic screening in cancer and CSC models. The cytotoxic effect was determined in breast (MCF-7) and colon (HCT-116) tumor cell lines using MTT assays. Then, the formation of MCF-7-derived mamospheres and HCT-116-derived colonospheres was evaluated in the presence of a noncytotoxic concentration of the compounds, using bright-field microscopy. As shown in Table S1, compounds **4b, 5a, 5b, 6a**, and **6b** were able to completely inhibit the formation of tumorspheres, a characteristic growth in CSCs, without affecting the viability of differentiated tumor cells. To select those compounds endowed with a good balance between efficacy and safety, we subsequently determined if the compounds that are able to act on CSCs do not affect the viability of IMR90 fibroblasts, which are commonly used as control normal cells. From the data in Table S1, tetrahydrocarbazole **4b** was not toxic in nontumor cells at 5 μM, while tetrahydrobenzo[*c*]chromenes **5a, 5b, 6a**, and **6b** exhibited toxicity (20–57% cell viability @5 μM). Among them, compound **4b** (UCM-13369) induced low levels of cytotoxicity in tumor cells (96 and 70% cell viability), complete inhibition of tumorsphere formation, and no toxicity in fibroblasts (100% cell viability) (Table S1 and Figure S1A), and was selected for further studies.

### Compound UCM-13369 Targets Nucleophosmin 1 (NPM1) Protein

**Figure 3:**
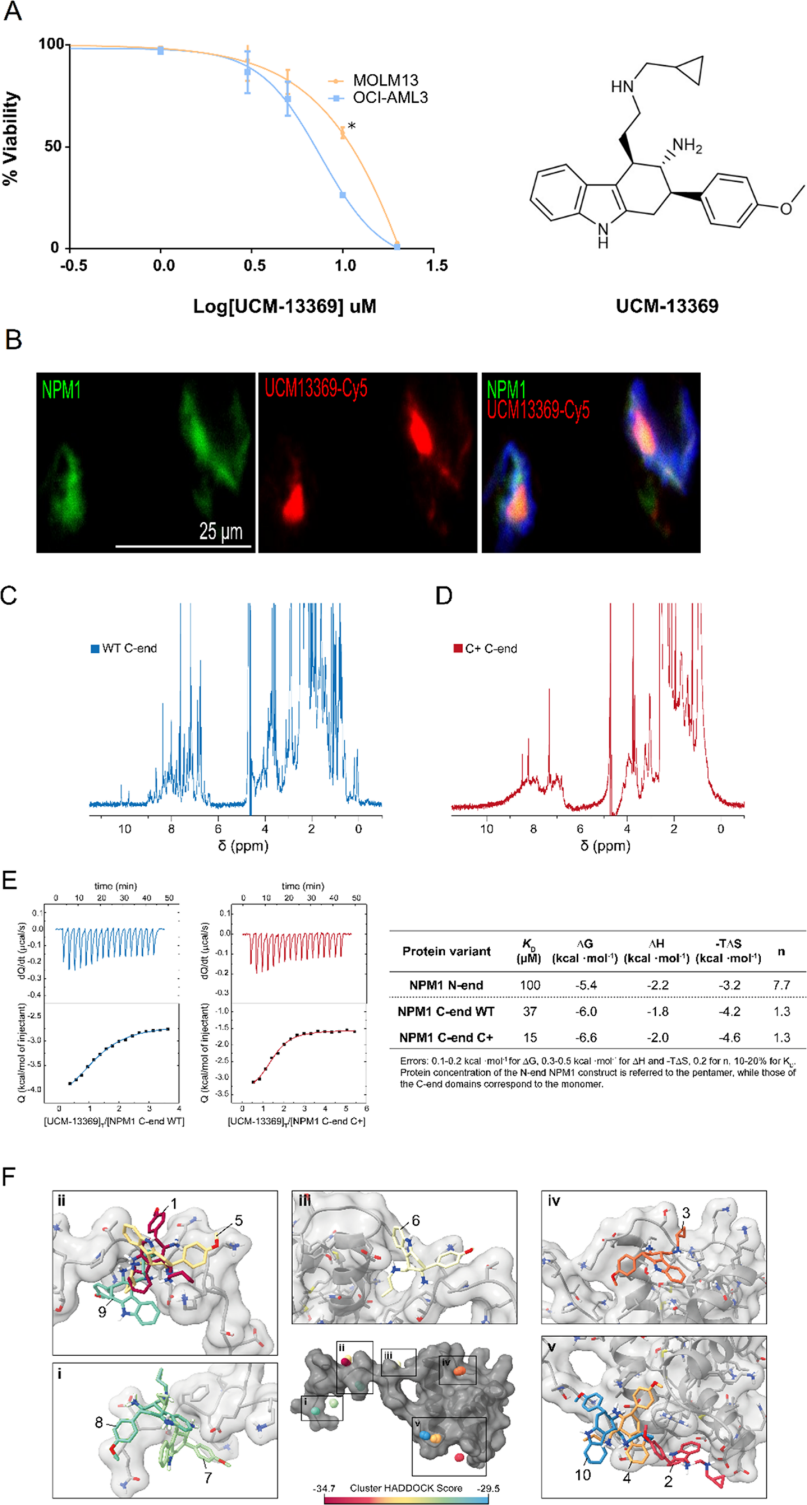
Compound UCM-13369 targets NPM1 protein, binding mainly through the C-end domain of the C+ mutant form. (A) Dose–response curves of UCM-13369 in AML cell lines MOLM13 (NPM1 WT) and OCI-AML3 (NPM1 C+). (B) Confocal microscopy images with staining of NPM1 (Alexa Fluor-488, green), UCM-13369-Cy5 (red), and nuclei (DAPI, blue) in OCI-AML3 cells. (C,D) 1D ^1^H NMR spectra of the C-end domain of NPM1 WT (C) and C+ (D). (E) ITC thermograms and binding isotherms of the interaction of UCM-13369 with the WT (blue) and C+ mutant (red) forms of NPM1. ITC-derived thermodynamic parameters of UCM-13369–NPM1 interaction. Gibbs free energy (Δ*G*), enthalpy (Δ*H*), entropic term (-TΔ*S*), equilibrium dissociation constant (*K*_D_), and binding stoichiometry (n). (F) Visualization of UCM-13369 binding interfaces to NPM1 C-end domain. The centroids of UCM-13369 in the four lowest-energy structures of each 10 clusters are plotted as balls colored according to the HADDOCK score, as indicated in the color legend. The structure of UCM-13369 of the top-ranked structure of each cluster is represented in the insets (i-v).

One of the most paradigmatic diseases with critical involvement of CSCs is AML. Among the AML stem cell population, CD34+/CD38- are the only ones capable of inducing leukemia *in vivo*.^27^ Hence, with the aim to decipher the molecular targets of UCM-13369, 1D-nano LC ESI-MSMS proteomic analysis was performed in CD34+ cells before and after 24 h of treatment with 5 μM UCM-13369. The results showed multiple differentially expressed proteins. We focused on proteins that disappeared completely after treatment with the compound and whose concentration could be quantified in untreated samples. According to this profile, 340 proteins, including targets related with stemness and cancer, were identified (see Supplementary Excel file). Among them, nucleophosmin 1 (NPM1) especially attracted our attention, as it is one of the most common mutated proteins in AML.^28^ To support that the inhibitory capacity of UCM-13369 observed in CSC cells (see Table S1) is mediated by NPM1, the compound was tested on OCI-AML3 and MOLM13 cell lines, as *in vitro* AML models bearing mutant (NPM1 C+) and wild-type (WT) protein, respectively. As shown in Figure 3A, the compound exhibits cytotoxicity against both cell lines in the μM range. In addition, different efficacies of UCM-13369 were observed between both AML models, being higher in NPM1 C+ expressing cells (OCI-AML3: IC_50_ = 7.26 μM, MOLM13: IC_50_ = 10.89 μM, *p*-value ≤0.01, Figure 3A).

To further support NPM1 as a molecular target of UCM-13369, we synthesized UCM-13369-Cy5 (Figure S2), a derivative of UCM-13369 containing the fluorescent tag Cy5, as a chemical probe for use in confocal microscopy. As shown in Figure 3B, we visualized the overlapping of UCM-13369-Cy5 (in red) with an Alexa Fluor-488-conjugated NPM1 primary antibody (in green), indicating colocalization of the compound with the protein in OCI-AML3 cells (see video in the Supporting Information).

To confirm a direct interaction of UCM-13369 with NPM1 and to address which domain of the protein would be responsible for binding the compound, pure protein samples of the pentameric N-end domain of NPM1, along with the C-terminal DNA-binding domains of WT and mutant NPM1 C+, were used in calorimetry assays. As a confirmation of its native state, analysis of the 1D ^1^H NMR spectrum of NPM1 C-end WT showed well-dispersed sharp peaks corresponding to proper protein folding (Figure 3C). In contrast, the spectrum obtained for the C+ mutant variant exhibited drastically less dispersed and broader signals, particularly in the amide region (6–10 ppm) (Figure 3D). This is indicative of a lower diversity in the chemical environment of amide protons consistent with the previously reported protein unfolding.^29^ The lower signal intensities in this region also suggest more pronounced relaxation effects, which might be related to the higher aggregation tendency of the mutant.^30^ Isothermal titration calorimetry (ITC) experiments revealed that the interaction between the NPM1 protein and UCM-13369 occurs mainly through the DNA-binding domain C-end of NPM1, with the C-end of the NPM1 C+ variant showing a 2-fold higher affinity toward UCM-13369 than the WT domain (Figure 3D). On the other hand, the pentameric construct of the NPM1 N-end, which lacks the AML-related region, yielded a much lower binding affinity (*K*_D_ ca. 100 μM range, Figure 3D). Interestingly, the interaction is entropically driven in all cases. Altogether, these data suggest that the new NPM1 inhibitor UCM-13369 binds specifically to the C-end domain of the C+ mutant protein.

To identify putative binding sites of UCM-13369 in this region of NPM1, *ab initio* docking predictions were performed using HADDOCK software.^31,32^ Docking solutions were clustered in 10 groups distributed across the surface of this domain, suggesting that UCM-13369 explores the surface of NPM1 C-end domain stochastically in the WT construct (Figure 3E, Figure S4, and Table S2). Notably among these, cluster 3 matched with a previously reported NPM1 binding site of a small molecule that would allow the conformation stabilization of the C+ mutant.^33^ In line with this finding, this cluster exhibited the lowest desolvation energy and harbored the second largest buried surface area of all analyzed docking clusters (Table S2). Further work should aim to confirm whether enhanced disorder in the NPM1 C+ species results in the unraveling of cryptic binding sites that lead to (i) a higher binding affinity to UCM-13369 and (ii) the stabilization of NPM1 conformational states.

### Unraveling the Molecular Mechanism of the New NPM1 Inhibitor UCM-13369

**Figure 4:**
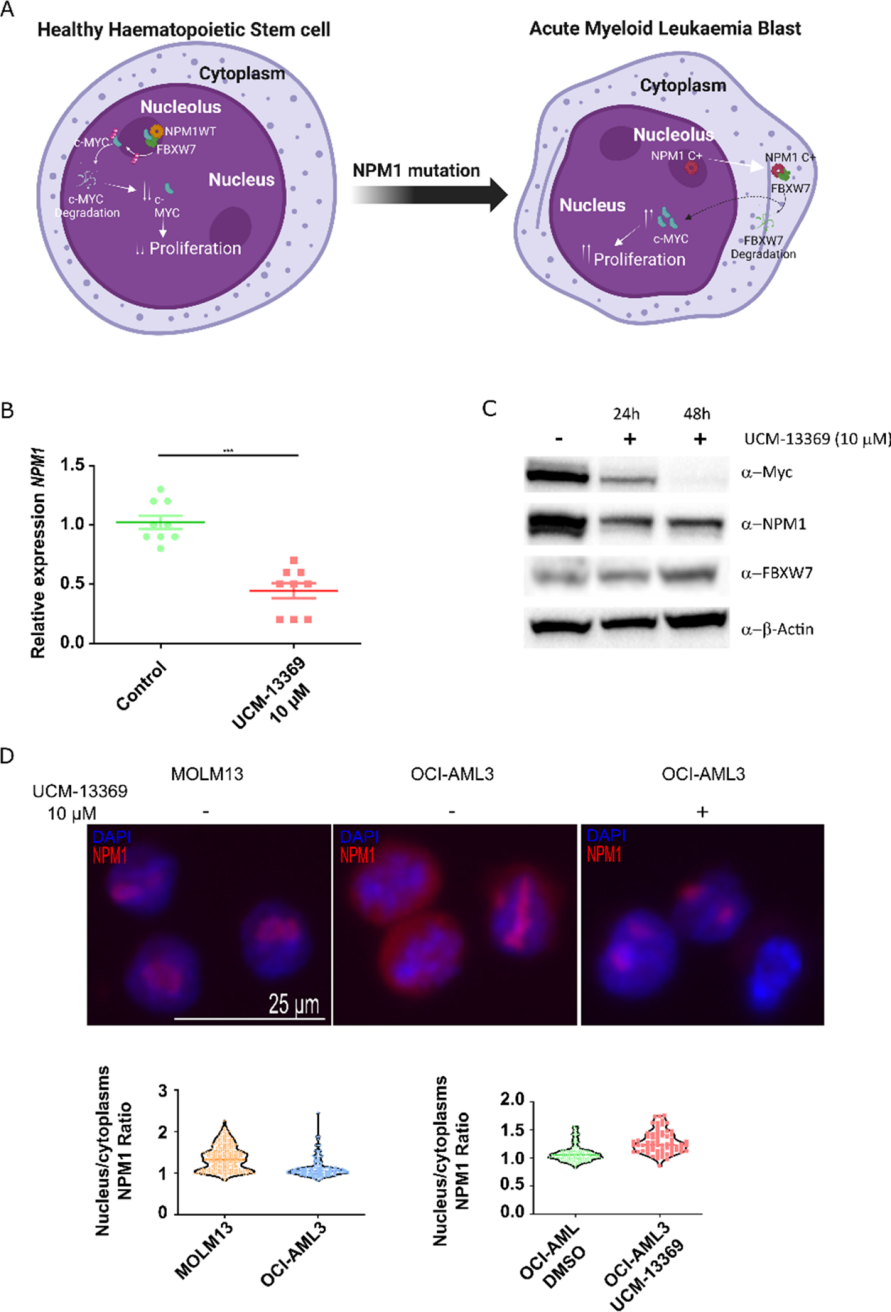
UCM-13369 inhibits gene and mutant NPM1 C+ protein expression and restores nucleolar localization. (A) Schematic representation of the molecular signaling pathways of NPM1 mutation in AML. (B) Dot plot showing decreased NPM1 expression with UCM-13369 treatment (10 μM) in OCI-AML3 cells. (C) Western blot showing decreased NPM1 and c-Myc expression and increased FBXW7 expression with UCM-13369 treatment (10 μM) in OCI-AML3 cells at 24 and 48 h. Densitometry values for NPM1: reduction of 50% and 70%, respectively. (D) Top: Confocal microscopy images with NPM1 (Alexa Fluor-647, red) and nuclei (DAPI, blue) staining in MOLM13, OCI-AML3, and UCM-13369-treated OCI-AML3 cells (10 μM). Bottom: Violin plot of NPM1 nucleus/cytoplasm ratio in MOLM13 and OCI-AML3 cell lines, and in untreated and UCM-13369-treated OCI-AML3 cells.

We next studied the impact of UCM-13369 in the genes and/or expression levels of NPM1 and NPM1 C+ pathway components (Figure 4A), including the positive regulation of c-Myc^34^ and the negative regulation of FBXW7 and the pro-apoptotic CASP8.^35^ Upon treatment with the compound in OCI-AML3 cell line, qPCR and Western blot revealed reduced levels for NPM1 and c-Myc, while FBXW7 was found to be upregulated (NPM1 *p*-value ≤0.0001, Figure 4B,C). The inhibition of NPM1 was also observed in MOLM13 cells expressing WT NPM1 (Figure S1B).

To further elucidate the mechanism of action of UCM-13369, we studied the influence of the compound on the subcellular localization of the protein by confocal microscopy, using an Alexa Fluor-647-conjugated NPM1 primary antibody (Figure 4D). In OCI-AML3 cells, we visualized that UCM-13369 induced the reduction of the cytoplasmic form of the protein, typically associated with the NPM1 mutation (NPM1 C+),^36^ restoring the nucleolar localization phenotype of the protein, more similar to the WT state of NPM1 in MOLM13 cells.

### Validation of the Therapeutic Potential of UCM-13369 in AML Patient Cells

**Figure 5:**
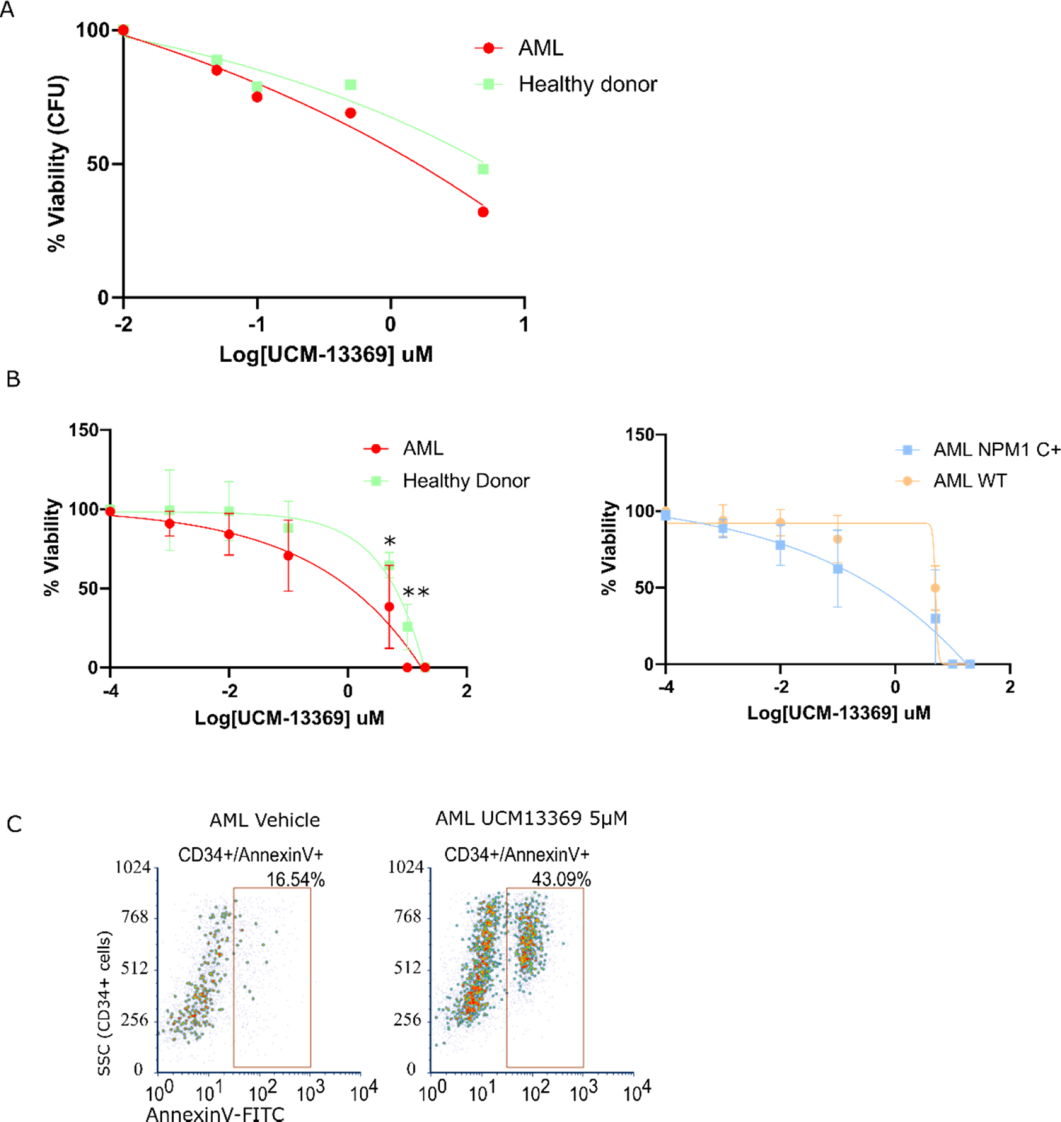
UCM-13369 has efficacy against AML cell lines and primary cells. (A) Dose–response curve of UCM-13369 in colony-forming unit (CFU) assays performed with primary HSCs bone marrow mononuclear fraction from healthy donors and AML patients. (B) Left: Dose–response curve of UCM-13369 in short-term cultures of HSCs performed with primary HSCs from bone marrow mononuclear fraction from healthy donors and AML patients. Right: Dose–response curve of the same assay with samples from AML patients with NPM1 WT vs NPM1 C+. (C) Dot plot of a representative sample from flow cytometry analysis using CD34-PE/Annexin V-FITC staining of short-term cultures of primary HSCs from AML patients performed with primary HSCs from bone marrow mononuclear fraction with and without treatment.

Considering the inhibitory capacity of UCM-13369 on AML cell lines (shown in Figure 3A), the therapeutic potential was assessed in primary CD34+ cells from AML patients. Two *ex vivo* models were used from AML patients and healthy donors: CD34+ liquid HSC (hematopoietic stem cell) culture as a short-term model and CFU-E (colony-forming unit–erythroid) as a midterm model. Primary CD34+ cells were sensitive to UCM-13369, exhibiting decreased viability, as shown in Figure 5A for CFU-E (IC_50_ = 0.30 μM for AML patients, IC_50_ = 1.73 μM for healthy donors, *p*-value ≤0.0001) and in Figure 5B for liquid HSC culture. Interestingly, the compound displayed higher efficacy against primary cells in AML patients than in healthy donors (IC_50_ = 1.877 and 6.399 μM, respectively, *p*-value ≤0.0001). Moreover, the compound showed higher efficacy in patients with NPM1 C+ AML (IC_50_ = 1.725 μM, *n* = 4) than in WT NPM1 AML patients (IC_50_ = 3.774 μM, *n* = 3, *p*-value ≤0.007) (Figure 5B).

The inhibition of NPM1, especially NPM1 C+ pathway (as shown in Figure 4), by UCM-13369 correlates with the increase of apoptosis observed in treated primary CD34+ cells upon staining with Annexin V (Figure 5C).

### Efficacy in an AML Animal Model

**Figure 6:**
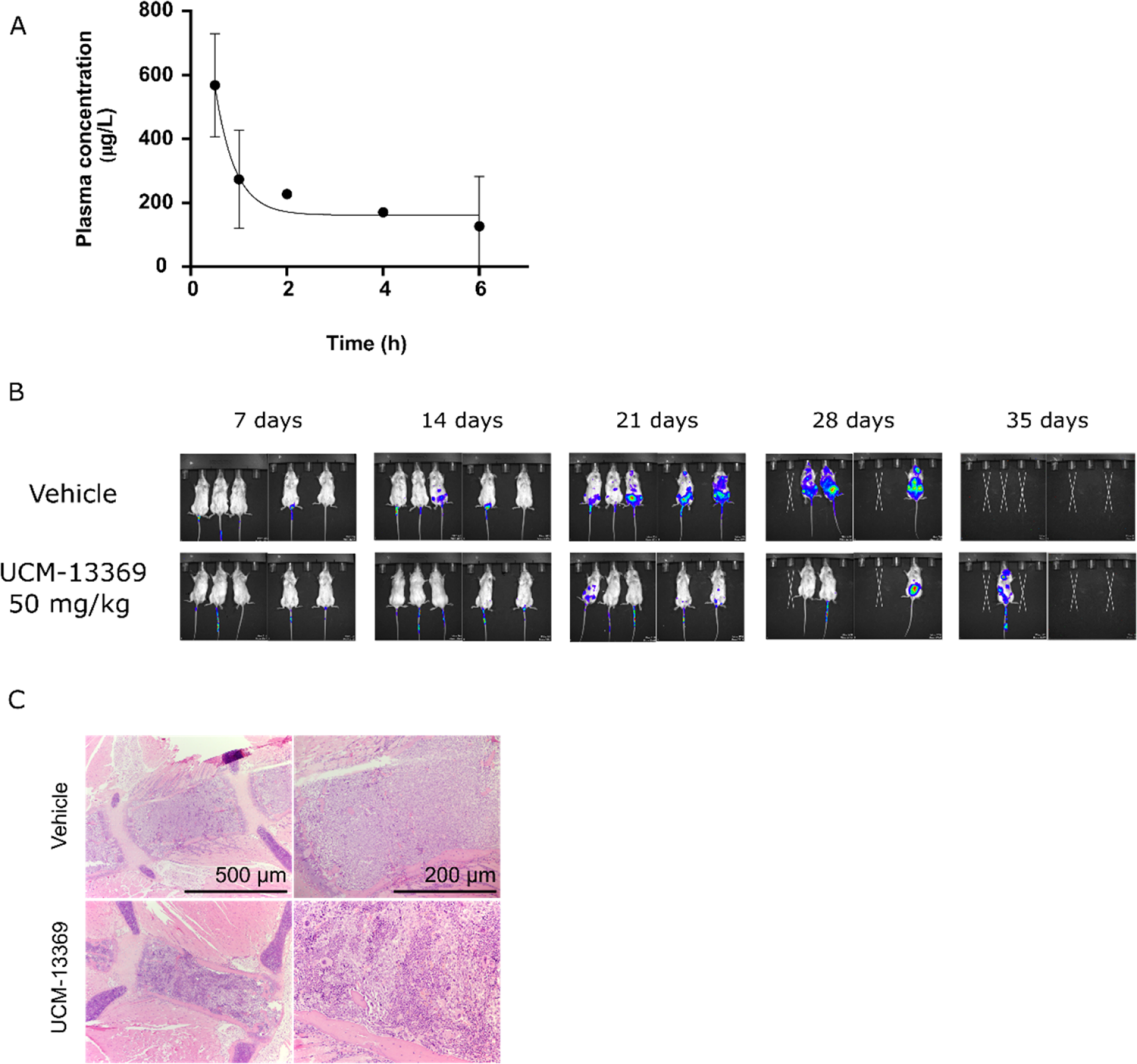
UCM-13369 shows efficacy against AML cells *in vivo*. (A) Values of UCM-13369 concentration *in vivo* at different time points after injection (25 mg/kg) analyzed by HPLC-MS. (B) Bioluminescence images acquired using an *in vivo* imaging system of NSG mice after injection of OCI-AML3 cell line with and without UCM-13369 treatment (50 mg/kg). (C) Microscopy analysis of the OCI-AML3 infiltration by H&E staining slides of bone marrow samples from NSG mice with paired time points after engraftment with and without UCM-13369 treatment (50 mg/kg).

Based on the encouraging results obtained in *ex vivo* models of AML, we further assessed the therapeutic potential of the new NPM1 inhibitor *in vivo*. Prior to the evaluation of the compound in an animal model of AML, *in vivo* pharmacokinetic studies were addressed. Following a single intraperitoneal administration of UCM-13369 at a dose of 25 mg/kg, levels of 200–600 μg/L were detected in mouse peripheral blood up to 6 h (Figure 6A). Plasma protein binding of the compound in mouse serum was measured using an equilibrium dialysis assay, indicating a bound fraction of 83 ± 1%. Next, the efficacy of the compound was evaluated in immunodeficient mice after injection of OCI-AML3-ffLuc-GFP cells as an AML animal model. Notably, as shown in Figure 6B, the UCM-13369-treated group (50 mg/kg, tail vein injection) showed a reduction of tumor infiltration compared to untreated group at end point (the reduction in tumor mass was negligible in 25-mg/kg-treated animals, data not shown). However, this effect did not result in an increase in survival as no difference was observed between treated and untreated animals (Figure S1C). Hematoxylin-eosin (H&E) staining of the bone marrow revealed less tumor cell infiltration in treated mice, supporting the efficacy of the compound against malignant cells (Figure 6C). Caspase-3 pathology analysis of treated mice revealed the absence of toxicity in bone marrow and liver (data not shown), while some caspase-3 cleavage was observed in the kidney, pointing to some toxicity in this organ, although most part of this staining was unspecific (Figure S1D). Indeed, the dose–response viability assay in human renal proximal tubule epithelial cells (hRPT Epi Cells) revealed toxicity (Figure S1E), suggesting that renal toxicity is one of the probable causes of lower-than-expected survival of treated AML animals.

## Discussion

In the past decade, the human microbiota has emerged as one of the most important topics in cancer research, as multiple associations with cancer development, progression, and response to treatment have been reported.^4,37,38^ Considering the cancer protective effect of several microbiota metabolites,^39−41^ in the present work, we describe the design, screening, and validation of novel small molecules inspired on microbiota metabolites in order to identify new drug candidates for cancer treatment. We designed a set of new chemotypes containing privileged scaffolds present in selected metabolites^22^ (Figure 1) and obtained them following synthetic routes based on asymmetric organocatalytic reactions (Figure 2). This valuable methodology provides complex chemotypes characterized by the presence of chiral carbon centers, which also address the demand for compounds with an increased number of sp^3^ carbon atoms, a parameter that has achieved great significance in successfully guiding drug research in recent years.^42^ Following a phenotypic drug discovery approach, our synthetic microbiota-inspired molecules were screened in CSC models. Among the different cells that contribute to tumor growth, CSCs have been described as a subpopulation of cells that potentially originate the cancer process and contribute to expansion, resistance, recurrence, and metastasis after therapy.^43−45^ We selected the compound UCM-13369 (**4b**) that showed capacity to inhibit tumorsphere growth of CSCs and confirmed its safety against healthy cells (Table S1). Differential proteomic profiling in CD34+ stem cells was analyzed to unravel the molecular target(s) associated with the efficacy of UCM-13369 against CSCs. Among the different stem- and cancer-related proteins that were ablated by the compound (see Supplementary Excel file), NPM1 stands out as a particularly relevant protein in AML, a hematological disease with a high mortality rate.^28^ AML affects the hematopoietic system through abnormal proliferation of myeloid cells in the bone marrow and ultimately suppresses the production of healthy blood cells. The standard treatment for AML for approximately 50 years has consisted of a combination of cytarabine (an antimetabolic agent that generates DNA damage in S-phase cells) and an anthracycline (an intercalating agent) in a program known as the “7 + 3” regimen. This intensive treatment is only effective in a small subset of AML patients and the therapeutic landscape has broadened in the last five years, driven by the development of targeted therapies focused on genetic mutations present in the disease.^46,47^ NPM1 and fms-like tyrosine kinase 3 (FLT3) are the most frequent mutations in AML, with an incidence of more than 50%,^48^ and therapies targeting specific mutations appear to have great potential to improve prognosis and treatment. Thus, the combination of the 7 + 3 regimen with mutant FLT3 inhibitors, such as midostaurin (Rydapt), improves response rates and outcomes in 20% of diagnosed patients whose leukemia has this mutation.^49,50^ Therefore, primary and secondary resistance remains a critical problem for most patients and treatment failure remains common. Although the NPM1 mutation is considered a good prognostic marker, even in this scenario, the 5-year rate of NPM1-mutated cases is below 50% overall survival and the crude numbers of deceased AML patients harbor NPM1 mutation, mainly due to the high incidence of this genetic alteration.^51^ Due to its biological and clinical characteristics, AML with NPM1 mutation was recognized in 2017 as a distinct entity in the WHO classification of hematopoietic malignancies. Unfortunately, there are no NPM1 inhibitors currently used in clinical practice, and a few candidate drugs are in clinical trials, such as NucAnt 6L and CIGB-300, but only ATRA is being studied as an adjuvant for the treatment of AML.^52,53^ Therefore, the development of new targeted therapy treatments is critical for the future of patients, to improve the dramatically low survival rate of AML.

An optimal therapeutic strategy against AML is expected to be a targeted therapy that abrogates the CSC population. Interestingly, the cytotoxicity against CSCs observed for UCM-13369 (Table S1) was mediated by NPM1, as the compound exhibited efficacy in the micromolar range in AML cell lines expressing WT and mutant NPM1 C+, especially in mutant cells (Figure 3A). Our results were validated in AML patient cells, in which UCM-13369 induced decreased viability while we observed lower cytotoxicity in healthy donor cells (Figure 5A,B), confirming the therapeutic window opportunity of the compound. In addition, greater efficacy was observed in AML patients harboring the NPM1 C+ mutation than in those lacking it (Figure 5B). This therapeutic potential was translated to a mouse model of AML with NPM1 C+ mutation, as intraperitoneal administration of UCM-13369 resulted in a reduction of tumor infiltration compared to the untreated group, although no increase in mice survival was observed (Figure 6B,C).

NPM1 is a nucleolar phosphoprotein, whereas the mutant form designated as NPM1 C+ protein translocates to the cytoplasm and confers undifferentiating properties to myeloid stem cells that transform into CSCs, being responsible for the stem cell signature characteristic of AML.^54−62^ Notably, the new compound, by interacting specifically with the C-end monomeric DNA-binding domain of NPM1 C+ form, was able to restore the nucleolar localization phenotype of the protein, reducing the presence of NPM1 in the cytoplasm of OCI-AML3 cells (Figure 4D), which is characteristic of the mutation and critical for the maintenance of the disease.^36^ In addition, upon UCM-13369 treatment, we observed downregulation of the pathway associated with the NPM1 C+ form^34,35^ (Figure 4B,C) and activation of stem cell apoptosis (Figure 5C). These results further support the mechanism of action of the compound through the mutant NPM1 pathway, which explains why the inhibitor has a higher efficacy in NPM1-mutated cells compared to NPM1-expressing cells. Moreover, cellular colocalization and ITC measurements of the compound with the protein (Figure 3B,C) confirmed the formation of the NPM1-UCM-13369 complex, validating the NPM1 protein as a molecular target of the new small-molecule targeting AML.

In summary, we disclose the phenotype-guided discovery of UCM-13369, a novel small molecule inspired on microbiota metabolites. Together, our results revealed that the activity of UCM-13369 in CSCs is linked to the regulation of the NPM1 protein and support the potential of the new synthetic inhibitor especially for the treatment of NPM1-mutated AML. The efficacy exhibited by UCM-13369 in *ex vivo* and *in vivo* AML models establishes tetrahydrocarbazole chemotype as worthy of further advancement as a promising therapeutic opportunity for the targeted treatment of leukemia.

## Methods

### Synthesis of *tert*-Butyl-2-(4-methoxyphenyl)-3-nitro-4-(2-oxoethyl)-1,2,3,4-tetrahydro-9*H*-carbazole-9-carboxylate, **16**

To a solution of (*S*)- or (*R*)-2-{diphenyl[(trimethylsilyl)oxy]methyl}pyrrolidine (0.20 equiv) in toluene (2 mL/mmol), benzoic acid (0.20 equiv) was added and the mixture was stirred at rt for 10 min under air. Then, *tert*-butyl-2-methyl-3-[(1*E*)-3-oxoprop-1-en-1-yl]-1*H*-indole-1-carboxylate (1.50 equiv) and *trans*-4-methoxy-β-nitrostyrene (1.00 equiv) were added and the reaction was heated at 110 °C under MW irradiation for 5.5 h. Afterward, the crude was flushed through a short plug of silica, using a 1:1 mixture of DCM/diethyl ether, and the solvent was evaporated under reduced pressure. The residue was purified by flash chromatography (toluene/diethyl ether, 98:2) to afford the title compound (2*S*,3*S*,4*S*)- or (2*R*,3*R*,4*R*)-**16**.

(2*S*,3*S*,4*S*)-**16** (**16a**) was obtained from *trans*-4-methoxy-β-nitrostyrene (213 mg, 1.18 mmol), using the *S* enantiomer of the catalyst (77 mg, 0.24 mmol), as a yellow solid (294 mg, 53%). The spectroscopic data were consistent with those previously reported.^24^*R*_f_: 0.30 (hexane/EtOAc 8:2). [α]_20_^D^ = −15.9 (*c* = 0.97, CHCl_3_) lit.^24^ [α]_20_^D^ = −12.01 (*c* = 0.85, CHCl_3_). ^1^H NMR (CDCl_3_): δ 1.64 (s, 9H, 3CH_3_), 2.97 (dd, *J* = 18.7, 2.2, 1H, 1/2CH_2_CHO), 3.25–3.38 (m, 2H, H_1_, 1/2CH_2_CHO), 3.52–3.62 (m, 2H, H_1_, H_2_), 3.79 (s, 3H, OCH_3_), 4.13–4.21 (m, 1H, H_4_), 5.21 (dd, *J* = 10.7, 9.1, 1H, H_3_), 6.87 (d, *J* = 8.7, 2H, H_3′_, H_5′_), 7.22 (d, *J* = 8.8, 2H, H_2′_, H_6′_), 7.21–7.33 (m, 2H, H_6_, H_7_), 7.39 (d, *J* = 7.8, 1H, H_5_), 8.14 (d, *J* = 7.8, 1H, H_8_), 9.71 (s, 1H, CHO). 1D ^1^H NMR NOE: irradiation of the signal at δ 5.21 ppm (dd, H_3_) yielded NOE on 2.97(dd, 1/2CH_2_CHO), and 7.22 (d, H_2′_, H_6′_).

(2*R*,3*R*,4*R*)-**16** (**16b**) was obtained from *trans*-4-methoxy-β-nitrostyrene (168 mg, 0.93 mmol), using the *R* enantiomer of the catalyst (61 mg, 0.19 mmol), as a yellow solid (190 mg, 45%, er = 96:4). [α]_20_^D^ = +11.8 (*c* = 1.31, CHCl_3_). Spectroscopic data were in agreement with those described for enantiomer **16a**. The er was determined after reduction of **16b** to the corresponding alcohol (2.5 equiv NaBH_4_, methanol, rt, 3 h, 92%) and chiral HPLC analysis (Chiralpak IC, 5 μm, 4.6 mm × 250 mm; 80:20 hexane/*i*-PrOH, flow rate 1.00 mL/min, λ = 254 nm, t_R_(major) = 13.22 min, t_R_(minor) = 18.42 min).

### Synthesis of *tert*-Butyl-4-{2-[(cyclopropylmethyl)amino]ethyl}-2-(4-methoxyphenyl)-3-nitro-1,2,3,4-tetrahydro-9*H*-carbazole-9-carboxylate, **25**

#### (2*S*,3*S*,4*S*)-**25** (**25a**)

Following general procedure B for reductive amination (see the Supporting Information) using **16a** (225 mg, 0.48 mmol) and (cyclopropylmethyl)amine (80 μL, 0.97 mmol), compound **25a** was obtained as an oil (218 mg, 86%), which was used in the next step without further purification. R_f_: 0.48 (DCM/ethanol 9:1). [α]_20_^D^ = −13.8 (*c* = 1.00, CHCl_3_). IR (ATR): ν 1730 (C=O), 1550 (NO_2_), 1251 (COC). ^1^H NMR (CDCl_3_): δ 0.02–0.07 (m, 2H, CH_2cpr_), 0.39–0.45 (m, 2H, CH_2cpr_), 0.80–0.93 (CH_cpr_), 1.64 (s, 9H, 3CH_3_), 1.96–2.07 (m, 1H, 1/2NHCH_2_CH_2_), 2.29–2.43 (m, 3H, 1/2NHCH_2_CH_2_, NHCH_2_CH), 2.46–2.55 (m, 1H, 1/2NHCH_2_CH_2_), 2.61–2.70 (m, 1H, 1/2NHCH_2_CH_2_), 3.18–3.28 (m, 1H, H_1_), 3.42–3.56 (m, 2H, H_1_, H_2_), 3.79 (s, 3H, OCH_3_), 3.88–3.93 (m, 1H, H_4_), 5.25–5.32 (m, 1H, H_3_), 6.87 (d, *J* = 8.7, H_3′_, H_5′_), 7.20–7.33 (m, 2H, H_6_, H_7_), 7.21 (d, *J* = 8.7, 2H, H_2′_, H_6′_), 7.59 (d, *J* = 7.1, 1H, H_5_), 8.12 (d, *J* = 7.7, 1H, H_8_). ^13^C NMR (CDCl_3_): δ 3.4 (CH_2cpr_), 11.2 (CH_cpr_), 28.4 (3CH_3_), 30.6 (NHCH_2_CH_2_), 32.9 (C_1_), 38.5 (C_4_), 45.4 (NHCH_2_CH_2_), 45.5 (C_2_), 54.9 (NHCH_2_CH), 55.4 (OCH_3_), 84.4 (C(CH_3_)_3_), 93.6 (C_3_), 114.4 (C_3′_, C_5′_), 115.3 (C_4a_), 115.8 (C_8_), 119.1 (C_5_), 123.1, 124.2 (C_6_, C_7_), 127.9 (C_8a_), 128.8 (C_2′_, C_6′_), 131.1 (C_1′_), 134.3 (C_9a_), 136.3 (C_4b_), 150.4 (NCOO), 159.3 (C_4′_). HPLC (t_R_, min): 19.58. MS (ESI, *m*/*z*, %): 520.0 ([M + H]^+^, 100).

#### (2*R*,3*R*,4*R*)-**25** (**25b**)

Following general procedure B for reductive amination (see the Supporting Information) using **16b** (200 mg, 0.43 mmol) and (cyclopropylmethyl)amine (75 μL, 0.86 mmol), compound **25b** was obtained as an oil (186 mg, 83%), which was used in the next step without further purification. [α]_20_^D^ = +12.13 (*c* = 0.75, CHCl_3_). Spectroscopic data were in agreement with those described for enantiomer **25a**.

### Synthesis of *tert*-Butyl-3-amino-4-{2-[(cyclopropylmethyl)amino]ethyl}-2-(4-methoxy-phenyl)-1,2,3,4-tetrahydro-9*H*-carbazole-9-carboxylate, **26**

#### (2*S*,3*S*,4*S*)-**26** (**26a**)

Following general procedure C for nitro group reduction (see the Supporting Information) using **25a** (218 mg, 0.42 mmol), compound **26a** was obtained as an oil (220 mg, quantitative), which was used in the next step without further purification. R_f_: 0.34 (DCM/methanol/NH_3_ 9:1:0.1). [α]_20_^D^ = −15.1 (*c* = 1.00, CHCl_3_). IR (ATR): ν 1727 (C=O), 1363 (C–N), 1249 (COC). ^1^H NMR (CDCl_3_): δ 0.03–0.08 (m, 2H, CH_2cpr_), 0.40–0.46 (m, 2H, CH_2cpr_), 0.84–0.98 (m, 1H, CH_cpr_), 1.62 (s, 9H, 3CH_3_), 2.17–2.27 (m, 1H, 1/2NHCH_2_CH_2_), 2.31–2.36 (m, 1H, 1/2NHCH_2_CH_2_), 2.41 (d, *J* = 6.9, 2H, NHCH_2_CH), 2.53–2.62 (m, 1H, 1/2NHCH_2_CH_2_), 2.64–2.82 (m, 2H, H_2_, 1/2NHCH_2_CH_2_), 2.92–2.97 (m, 1H, H_4_), 3.09 (ddd, *J* = 18.0, 11.3, 2.6, 1H, H_1_), 3.19 (dd, *J* = 10.5, 8.5, 1H, H_3_), 3.35 (dd, *J* = 17.9, 4.3, 1H, H_1_), 3.82 (s, 3H, OCH_3_) 6.91 (d, *J* = 8.7, 2H, H_3′_, H_5′_), 7.19–7.28 (m, 2H, H_6_, H_7_), 7.22 (d, *J* = 8.4, 2H, H_2′_, H_6′_), 7.57 (dd, *J* = 6.8, 2.1, 1H, H_5_), 8.11 (dd, *J* = 7.1, 1.9, 1H, H_8_). ^13^C NMR (CDCl_3_): δ 3.5 (2CH_2cpr_), 11.1 (CH_cpr_), 28.4 (3CH_3_), 32.1 (NHCH_2_CH_2_), 33.7 (C_1_), 41.4 (C_4_), 46.9 (NHCH_2_CH_2_), 49.7 (C_2_), 55.1 (NHCH_2_CH), 55.4 (OCH_3_), 56.5 (C_3_), 83.8 (C(CH_3_)_3_), 114.4 (C_3′_, C_5′_), 115.6 (C_8_), 118.4 (C_4a_), 119.0 (C_5_), 122.7 (C_6_), 123.5 (C_7_), 128.9 (C_4b_), 129.1 (C_2′_, C_6′_), 135.2 (C_9a_), 135.4 (C_1′_), 136.4 (C_8a_), 150.6 (NCOO), 158.7 (C_4′_). HPLC (t_R_, min): 13.78. MS (ESI, *m*/*z*, %): 490.1 ([M + H]^+^, 100).

#### (2*R*,3*R*,4*R*)-**26** (**26b**)

Following general procedure C for nitro group reduction (see the Supporting Information) using **25b** (160 mg, 0.31 mmol), compound **26b** was obtained as an oil (152 mg, quantitative), which was used in the next step without further purification. [α]_20_^D^= +17.8 (*c* = 1.03, CHCl_3_). Spectroscopic data were in agreement with those described for enantiomer **26a**.

### Synthesis of 4-{2-[(Cyclopropylmethyl)amino]ethyl}-2-(4-methoxyphenyl)-2,3,4,9-tetrahydro-1*H*-carbazol-3-amine, **4**

To a solution of enantiomer **26a** or **26b** (1.00 equiv) in methanol (2.5 mL/mmol), HCl (35.0 equiv, 37% aq. solution) was added and the reaction mixture was stirred at rt for 4 h. Then, solvents were evaporated under reduced pressure and the resulting solid was washed with anhydrous diethyl ether and dried under vacuum to afford the corresponding hydrochloride salt of (2*S*,3*S*,4*S*) or (2*R*,3*R*,4*R*)-**4**.

(2*S*,3*S*,4*S*)-**4** (**4a**) was obtained from **26a** (85 mg, 0.17 mmol) as hydrochloride salt (42 mg, 50%, er = 96:4). R_f_: 0.30 (DCM/methanol/NH_3_ 9:1:0.1). [α]_20_^D^ = −5.4 (*c* = 0.24, methanol). Chiral HPLC (method J, t_R_, min): 4.74. IR (ATR): ν 3375 (NH), 1458 (C–N). ^1^H NMR (500 MHz, methanol-*d4*): δ 0.26–0.36 (m, 2H, CH_2cpr_), 0.55–0.65 (m, 2H, 2CH_2cpr_), 0.93–1.02 (m, 1H, CH_cpr_), 2.21–2.43 (m, 2H, NHCH_2_CH_2_), 2.64–2.82 (m, 3H, NHCH_2_CH, 1/2NHCH_2_CH_2_), 3.01–3.24 (m, 3H, 2H_1_, 1/2NHCH_2_CH_2_), 3.29–3.38 (m, 1H, H_2_), 3.48–3.55 (m, H_4_), 3.79 (s, 3H, CH_3_), 3.92–3.97 (m, 1H, H_3_), 6.96 (d, *J* = 8.3, 2H, H_3′_, H_5′_), 7.01–7.11 (m, 2H, H_6_, H_7_), 7.32 (d, *J* = 7.7, 1H, H_8_), 7.37 (d, *J* = 8.5, 2H, H_2′_, H_6′_), 7.56 (d, *J* = 7.7, 1H, H_5_). ^13^C NMR (125 MHz, methanol-*d4*): δ 4.4 (CH_2cpr_), 4.6 (CH_2cpr_), 8.13 (CH_cpr_), 27.8 (NHCH_2_CH_2_), 29.4 (C_1_), 37.6 (C_4_), 44.4 (C_2_), 45.1 (NHCH_2_CH_2_), 53.7 (NHCH_2_CH), 55.8 (CH_3_), 56.1 (C_3_), 106.7 (C_4a_), 112.3 (C_5_), 115.8 (C_3′_, C_5′_), 118.8 (C_8_), 120.4 (C_6_), 122.5 (C_7_), 127.4 (C_4b_), 130.3 (C_2′_, C_6′_), 132.7 (C_1′_), 134.8 (C_9a_), 138.3 (C_8a_), 161.0 (C_4′_). HPLC (t_R_, min): 11.45. MS (ESI, *m*/*z*, %): 390.1 ([M + H]^+^, 100). Elemental analysis calculated for C_25_H_31_N_3_O·2HCl·2H_2_O: %C 60.24, %H 7.98, %N 8.43; experimental: %C 60.98, %H 7.74, %N 8.11.

(2*R*,3*R*,4*R*)-**4** (**4b, UCM-13369**) was obtained from **26b** (100 mg, 0,2 mmol) as hydrochloride salt (40 mg, 40%, er = 95:5). [α]_20_^D^= +6.8 (*c* = 0.30, methanol). Chiral HPLC (method J, t_R_, min): 6.42. HRMS (ESI, *m*/*z*): calculated for C_25_H_32_N_3_O [M + H]^+^: 390.2540, found: 390.2526. Elemental analysis calculated for C_25_H_31_N_3_O·2HCl·2H_2_O: %C 60.24, %H 7.98, %N 8.43; experimental: %C 60.63, %H 7.68, %N 8.15. Spectroscopic data were in agreement with those described for enantiomer **4a**.

### Cell Viability Assays

The impact of the novel compound UCM-13369 on cell viability was assessed in cell lines, MCF-7, HCT-116, IMR90, OCI-AML3, and MOLM13; mamospheres and colonospheres; bone marrow mononuclear primary cells; and colony-forming unit assays. Cells were seeded at 3 × 10^4^ cells/mL in 96-well plates. The test compound was diluted in PBS. Cell lines were treated for 48 h with 4–6 different concentrations of UCM-13369 (10 μM, 5 μM, 1 μM, 0,5 μM, 0,1 μM) or vehicle (PBS). Viability was evaluated by MTT or WST-1 and dose/response curves were performed.

### Flow Cytometry

CFU-E and CD34+ primary cells with and without UCM-13369 treatment were analyzed. The following monoclonal antibodies were used: CD34-PE, Annexin V-FITC (BD Biosciences). The cells were incubated with antibodies in PBS on ice for 15 min in the dark. The analysis of CD34+ and Annexin V+ subpopulation was carried out in a flow cytometer FACSCalibur (Becton Dickinson, Citometría y Microscopía de Fluorescencia core facility, UCM) and the data were processed using FlowJo_V10 software.

### Proteomic Analysis

CD34+ from healthy donors (*n* = 3) were cultured for 24 h with and without 5 μM UCM-13369. Control and treated samples were harvested and pooled and protein extracts were obtained after cell lysis with urea-containing CHAPS buffer and sonication at 4 °C. Proteins were precipitated with methanol/chloroform and protein concentration was measured using Pierce 660 nm Protein Assay Reagent. For tryptic digestion, 10 μg of each depleted protein sample was individually dissolved in 8 M urea and 25 mM ammonium bicarbonate, reduced, and alkylated with iodoacetamide, according to a method previously described.^63^ Urea concentration was reduced to 2 M with 25 mM ammonium bicarbonate and the samples were digested overnight at 37 °C with trypsin (Sigma-Aldrich), with a 25:1 ratio. After digestion, the samples were desalted using ZipTip (Merck) as described. For liquid chromatography and mass spectrometer analysis, a 2 μg aliquot of each digested sample was subjected to 1D-nano LC ESI-MSMS analysis using a nano liquid chromatography system (Eksigent Technologies nano LC Ultra 1D plus, SCIEX, Foster City, CA) coupled to a high-speed Triple TOF 5600 mass spectrometer (SCIEX, Foster City, CA) with a Nanospray III source. The analytical column used was a silica-based reversed-phase Acquity UPLC M-Class Peptide BEH C18 Column, 75 μm × 150 mm, 1.7 μm particle size and 130 Å pore size (Waters). The trap column was a C18 Acclaim PepMap TM 100 (Thermo Scientific), 100 μm × 2 cm, 5 μm particle diameter, 100 Å pore size, switched online with the analytical column. The loading pump delivered a solution of 0.1% formic acid in water at 2 μL/min. The nanopump provided a flow rate of 250 nL/min and was operated under gradient elution conditions. Peptides were separated using a 250 min gradient ranging from 2% to 90% mobile phase B (mobile phase A: 2% ACN, 0.1% formic acid; mobile phase B: 100% ACN, 0.1% formic acid). The injection volume was 5 μL. The data acquisition was performed using a Triple TOF 5600 System (SCIEX, Foster City, CA). Data were acquired using an ionspray voltage floating (ISVF) 2300 V, curtain gas (CUR) 35, interface heater temperature (IHT) 150, ion source gas 1 (GS1) 25, declustering potential (DP) 100 V. All data were acquired using information-dependent acquisition (IDA) mode with Analyst TF 1.7 software (SCIEX). For IDA parameters, 0.25 s MS survey scans (mass range of 350–1250 Da) were followed by 15 MS/MS scans of 100 ms (mass range of 100–1800, total cycle time was 2 s). Switching criteria were: ion *m*/*z* greater than 350 and smaller than 1250, with a charge state of 2–5 and an abundance threshold of more than 90 counts (cps). Former target ions were excluded for 15 s. IDA rolling collision energy (CE) parameters script was used for automatically controlling the CE. The mass spectrometry data analysis and quantification were processed using PeakView 2.2 Software (SCIEX, Foster City, CA) and exported as mgf files. Protein data searches were performed either using Mascot Server v.2.6.1 or Peaks Studio 7.5 search engines against a target/decoy database built from sequences in the *Homo sapiens* reference proteome at Uniprot Knowledgebase. Search parameters were set as follows: trypsin enzyme; 2 missed cleavages were allowed; carbamidomethyl (C) as fixed modification and acetyl (Protein N-term) and oxidation (M) as variable modifications. Peptide mass tolerance was set to ±25 ppm for precursors and 0.02 Da for fragment masses. Spectral counting was used to perform a semiquantitative analysis (see Venn diagram in the Supporting Information and Supplementary Excel file).

### Confocal Microscopy

Cells were plated onto glass coverslips or Greiner Bio-One μClear black 96-well plate (7.5 × 10^4^ cells per well) and treated with compound UCM-13369 for 48 h. Then, cells were fixed with 4% paraformaldehyde for 15 min, permeabilized with 0.1% Triton X-100 for 30 min, and blocked in 5% bovine serum albumin for 1 h at rt. Incubation with primary antibodies against NPM1 (7H10B9, NOVUS Biologicals) was carried out overnight at 4 °C. Alexa Fluor-488-conjugated antimouse or antirabbit antibodies (Molecular Probes) were used for detection of the reported proteins by confocal microscopy. Nuclei were counterstained with 4′,6-diamidino-2-phenylindole (DAPI) bath. A total of 35 fields per well were acquired with ×40 magnification lens for those samples run in the Opera HCS system (PerkinElmer). Images were segmented using the DAPI staining and the coefficient of variation of NPM1 intensity per nucleus/cytoplasm was calculated using Definiens Developer XD software.

### Isothermal Titration Calorimetry

ITC experiments were performed in a Nano ITC Low Volume (TA Instruments) at 25 °C. The experiments consisted of 17 injections of 2.9 μL of 1 mM UCM-13369 in 20 mM sodium phosphate buffer (pH 7.2) with 150 mM NaCl and 1 mM TCEP into the sample cell, initially filled with 161 μL of 20 μM pentameric WT NPM1 N-end or 100 μM monomeric WT or C+ NPM1 C-end proteins. The injection interval was set to 180 s, allowing the thermal power signal to return to the baseline before the next injection. Homogeneous mixing was achieved by maintaining stirring speed at 300 rpm. The reference cell was filled with degassed ultrapure water. All samples were degassed prior to the experiments. Injection heats normalized per mole of injectant vs molar ratio data were fitted to an independent binding site model using Origin 7.0 (OriginLab) for the analysis.

### *In Vivo* Experiments

The animal studies were performed according to the guidelines of the Institutional Animal Care and Use Committees of the Comunidad de Madrid, Spain, under approved protocol (PROEX 023/17).

For the pharmacokinetic study of UCM-13369, after injecting 25 mg/kg dose of the compound, samples of blood were withdrawn at different times (30, 60, 120, 240, 360 min), and the presence of tested compound was analyzed by HPLC-MS. The area of the molecular peak allowed us to calculate the concentration of the compound present in mice serum at each time.

An AML xenograft mouse model was used for the *in vivo* study of efficacy. Female 6- to 8-week-old NOD.Cg-Prkdcscid Il2rgtm1Wjl/SzJ (NSG) mice (Vivotecnia, Madrid, Spain) were inoculated intravenously by tail vein injection with OCI-AML3-ffLuc-GFP cells (1 × 10^6^). Four days later, the mice were randomized into two groups of 10 animals: the first group received intraperitoneal injections of vehicle (0.9% saline solution) 5 days a week; the second group received intraperitoneal injections of 25 mg/kg or 50 mg/kg UCM-13369 five times weekly for 3 weeks. The mice were monitored for tumor burden, distribution, and engraftment every week by IVIS whole-body bioluminescence imaging. Bioimaging of MM burden *in vivo* was performed by IVIS Imaging system (Caliper Life Science). A solution of luciferin (D-luciferin monosodium salt, Thermo Fisher) in PBS was injected (150 mg/kg, ip), and luminescence signal was determined after 10 min. The data were analyzed using the Living Image 4.2 (Caliper Life Science) software.

Statistical analysis was performed using GraphPad Software, Inc., CA (version 6.0). All data are presented as mean ± SEM and are representative of three independent experimental replicates (*n* = 3). Normally distributed data were analyzed by unpaired Student’s *t* test or one-way ANOVA. For nonparametric data, Mann–Whitney test was used. Overall survival (OS) and PFS were analyzed by Kaplan–Meier curves. *p*-Values < .05 were considered statistically significant.

## Supporting Information Available

Additional results of *in vitro* and *in vivo* experiments (Figure S1), synthesis of probe UCM-13369-Cy5 (Figure S2), purification of NPM1 species and validation of the peptide sequence of NPM1 C-end C+ (Figure S3), statistical analysis of *ab initio* UCM-13369 binding site prediction in WT NPM1 C-end domain (Figure S4), phenotypic screening data of compounds **1**–**13** (Table S1), statistical analysis of HADDOCK data after clustering the solutions for the NPM1 C-end WT and UCM-13369 (Table S2); detailed description of the methods of all of the experiments performed: synthesis and characterization data for all compounds, cellular assays, NPM1 protein studies, and plasma protein binding (PPB) assay; NMR spectra for final compounds **1**–**13** and probe UCM-13369-Cy5 and HPLC traces (PDF)
Proteomic data (XLSX)
Colocalization of compound UCM-13369 with NPM1 protein (AVI)

## Supplementary Material

au3c00682_si_002.xls

au3c00682_si_003.avi
