# Supplementary material for: Cancer-Stem-Cell Phenotype-Guided Discovery of a Microbiota-Inspired Synthetic Compound Targeting NPM1 for Leukemia
Source: JACS Au. 2024 Feb 9;4(5):1786–800. doi: 10.1021/jacsau.3c00682 (PMC11134387; doi:10.1021/jacsau.3c00682)
Supplement: Supplementary file 1 — au3c00682_si_001.pdf [file au3c00682_si_001.pdf]

Supporting Information

Cancer-stem-cell phenotype-guided discovery of a microbiota-inspired synthetic compound targeting NPM1 for leukemia

Sergio Algar,[1,†] Henar Vázquez-Villa,[1,†] Pedro Aguilar-Garrido,[2,3] Miguel Ángel Navarro-Aguadero,[2,3] María Velasco-Estévez,[2,3] Anabel Sánchez-Merino,[1] Iván Arribas-Álvarez,[1] Alberto Paradela,[4] Rafael L. Giner-Arroyo,[5] Joaquín Tamargo-Azpilicueta,[5] Irene Díaz-Moreno,[5] Joaquín Martínez-López,[2,3] Miguel Gallardo,[2,3,] María L. López-Rodríguez[1,*], and Bellinda Benhamú[1,*]*

[1]Dep. of Organic Chemistry, Faculty of Chemistry, Universidad Complutense de Madrid; E-28040 Madrid, Spain

[2]Dep. of Haematology, Hospital Universitario 12 de Octubre, Instituto de Investigación Sanitaria Hospital 12 de Octubre (imas12); E-28041 Madrid, Spain

[3]H12O-CNIO Haematological Malignancies Clinical Research Unit, Spanish National Cancer Research Centre; E-28029 Madrid, Spain

[4]Proteomics Laboratory, CNB, CSIC; E-28049 Madrid, Spain

[5]Institute for Chemical Research, cicCartuja, University of Seville, CSIC; E-41092 Sevilla, Spain

Corresponding authors' emails: miguelgallardodelgado@gmail.com, mluzlr@ucm.es, bellinda.benhamu@quim.ucm.es

Table of Contents

1. Figures S1-S4 and Tables S1, S2 S2
2. Experimental methods S8
   2.1. Synthesis S8
   2.2. Cellular assays S46
   2.3. NPM1 protein studies S48
   2.4. Plasma protein binding (PPB) assay S50
3. NMR Spectra S52
4. HPLC Traces S66
5. References S78

1. Figures S1-S4 and Tables S1, S2

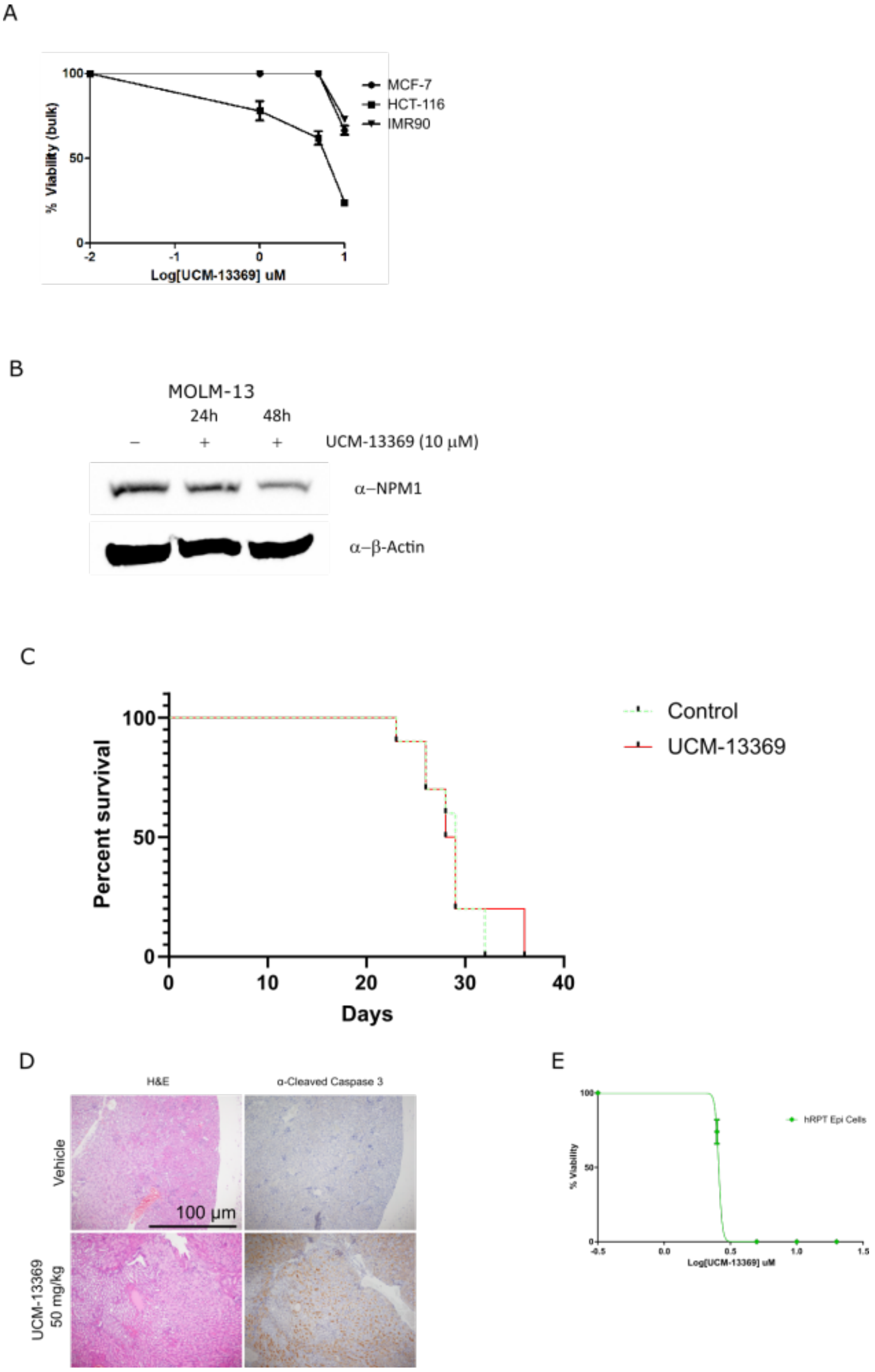

Figure S1. (A) Dose-response curve of UCM-13369 in MCF-7, HCT-116 and IMR90 cells. (B) Western blot showed downexpression of NPM1 with UCM-13369 treatment (10 μM) in MOLM13 cells at 24 and 48 h. Densitometry values: reduction of 5% and 44%, respectively. (C) Kaplan-Meir survival curve showed no significant differences between NSG mice injected with OCI-AML3 cells, with and without UCM-13369 treatment (50 mg/kg). (D) IHC analysis of apoptotic cells in a representative kidney sample from OCI-AML3 xenograft mice treated with DMSO (vehicle, top) vs. UCM-13369 treated mice (50 mg/kg, bottom) mice: H&E (left) and cleaved caspase-3 (right). Scale bar: 100 μm. (E) Dose-response curve of UCM-13369 in human renal proximal tubule epithelial cells (hRPT Epi Cells) showed toxicity in kidney cells.

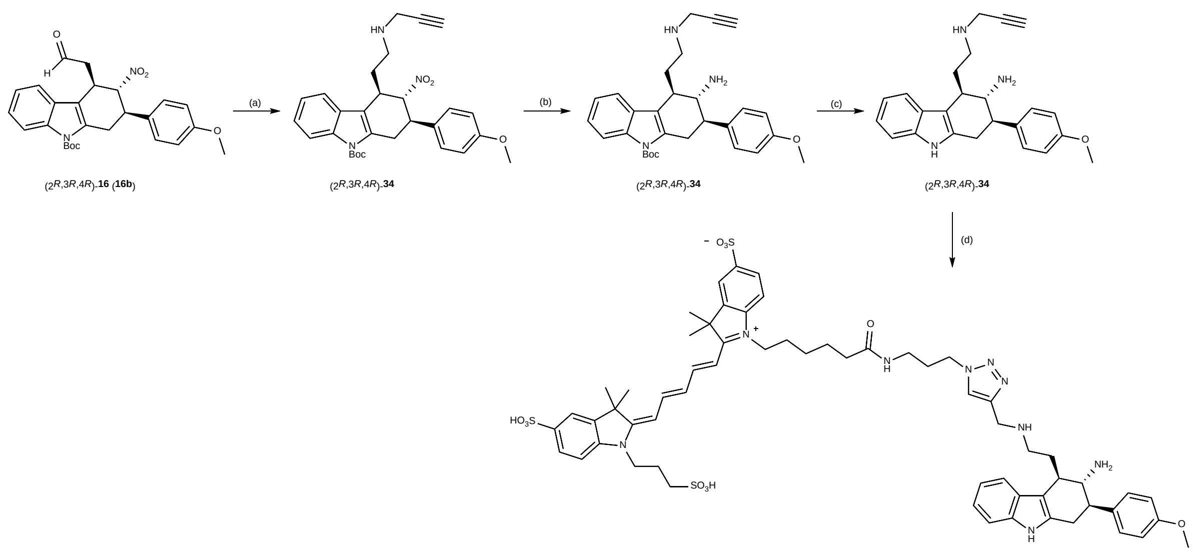

Figure S2. Synthesis of UCM-13369-Cy5 probe. Reagents and conditions: (a) i. prop-2-yn-1-amine, MeOH/DCM, rt, 3 h; ii. $NaBH_4$, rt, 2 h, 57%; (b) Zn, AcOH/MeOH, 2 h, 58%; (c) HCl, MeOH, rt, 4 h, 67%; (d) sulfo-cyanine 5 azide, $CuSO_4 \cdot 5H_2O$, sodium ascorbate, DMF/H_2O, rt, 36 h, 67%.

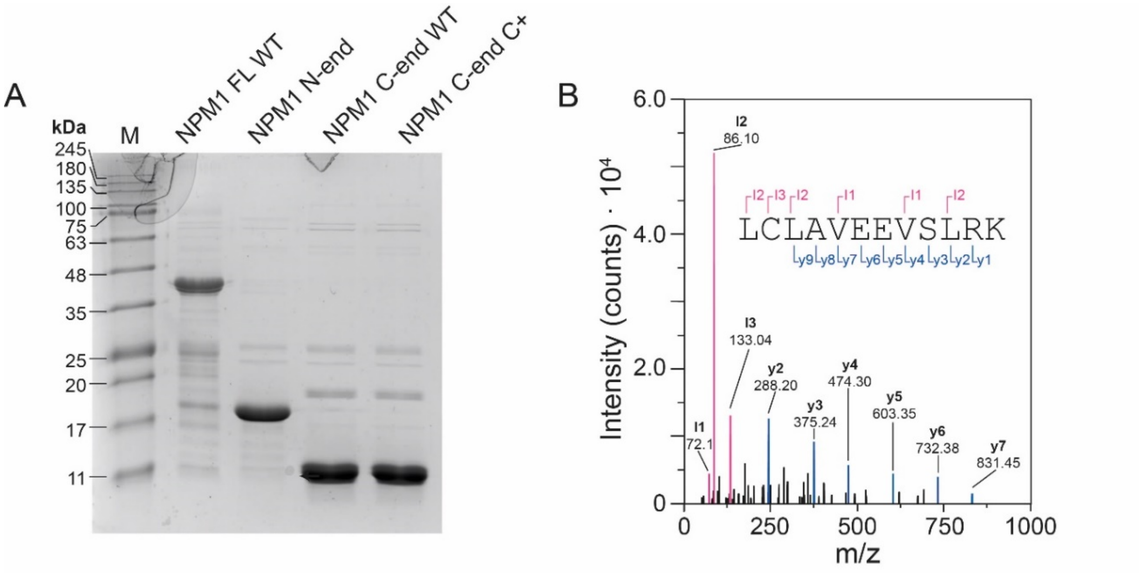

Figure S3. Purification of NPM1 species and validation of the peptide sequence of NPM1 C-end C+. (A) Coomassie Blue-stained 15% SDS-PAGE gel of NPM1 N-end domain and WT and C+ mutant C-end domains. 5 µg of protein was loaded. A molecular weight marker was loaded in the first lane. As reference, a sample of NPM1 full-length WT has been included **(B)** Identification of the LCLAVEEVSLRK peptide arising from the frameshift mutation in the gene encoding the NPM1 C-end C+ DNA binding domain by LC/MS. This peptide has been identified after tryptic digestion of the more intense protein band appearing in the SDS-PAGE (panel A, lane #5). Arrows indicate selected fragment ions used for identification of the peptide (l1, l2, l3, y2, y3, y4, y5, y6, y7).

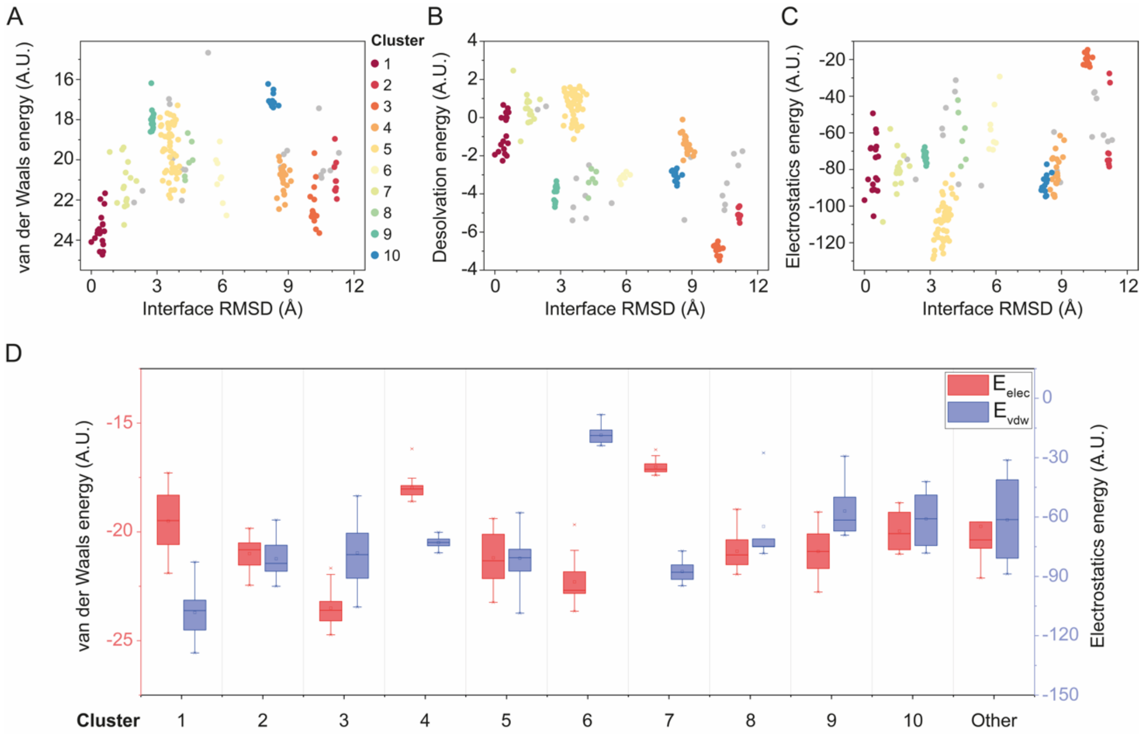

Figure S4. Statistical analysis of *ab initio* UCM-13369 binding site prediction in WT NPM1 C-end domain. (A-C) van der Waals, desolvation and electrostatic energy *vs*. interface RMSD from the lowest energy structure obtained for each complex obtained by HADDOCK. Dots were colored according to their assigned cluster, as indicated in the legend of panel A. (D) van der Waals and electrostatic contributions for complex formation in all clusters analyzed.

Table S1. Phenotypic screening data of compounds **1-13** in breast (MCF-7) and colon (HCT-116) cancer cell lines, and viability of active compounds in non-tumor IMR90 fibroblast cell line.[a]

Compound	Viability (%)		Inhibition of tumorsphere formation (%)		Viability (%)
	**MCF-7** (@10 µM)	**HCT-116** (@10 µM)	**MCF-7** (@10 µM)	**HCT-116** (@10 µM)	**IMR90** (@5 µM)[e]
**1a**	91 ± 7	70 ± 2	0	0	-
**1b**	80 ± 2	97 ± 2	0	0	-
**2a**	96 ± 3	90 ± 1	0	0	-
**2b**	97 ± 3	94 ± 3	0	0	-
**3a**	98 ± 2	95 ± 1	0	0	-
**3b**	95 ± 4	94 ± 3	0	0	-
**4a**	92 ± 7[b]	85 ± 2[c]	0[b]	0[c]	-
**4b**	96 ± 2[d]	70 ± 3[d]	100[d]	100[d]	100
**5a**	99 ± 1[b]	87 ± 1[b]	100[b]	100[b]	20 ± 5<br>83 ± 6[b]
**5b**	87 ± 1[b]	94 ± 6[b]	100[b]	100[b]	28 ± 7<br>98 ± 2[b]
**6a**	94 ± 11[d]	81 ± 6[b]	100[d]	100[b]	53 ± 12<br>95 ± 5[b]
**6b**	94 ± 5[d]	78 ± 9[b]	100[d]	100[b]	57 ± 13<br>0[b]
**7a**	98 ± 1	92 ± 1	0	0	-
**7b**	96 ± 2	95 ± 2	0	0	-
**8a**	65 ± 1	94 ± 6	0	0	-
**8b**	98 ± 1	93 ± 2	0	0	-
**9a**	73 ± 5	94 ± 2	0	0	-
**9b**	65 ± 2	90 ± 3	0	0	-
**10a**	89 ± 6	61 ± 8	4 ± 2	2 ± 1	-
**10b**	94 ± 5	70 ± 1	0	0	-
**11a**	92 ± 2	94 ± 2	0	0	-
**11b**	99 ± 1	94 ± 2	0	0	-
**12a**	90 ± 2	90 ± 4	0	0	-
**12b**	73 ± 3	92 ± 3	0	0	-
**13a**	77 ± 8	85 ± 7	0	0	-
**13b**	89 ± 6	90 ± 3	0	0	-

[a] Data from two to three independent experiments performed in triplicate; [b] @1 µM; [c] @0.1 µM; [d] @5 µM; [e] determined for compounds inhibiting CSC tumorsphere formation.

Table S2. Statistical analysis of HADDOCK data after clustering the solutions for the NPM1 C-end WT and UCM-13369

Cluster	RMSD (Å)	S	E_{desolv} (A.U.)	E_{vw} (A.U.)	E_{elec} (A.U.)	Buried Surface Area (Å^2)	HADDOCK score (A.U.)
1	0.3±0.2	18	-1.9±0.2	-23.4±0.8	-94.7±7.4	471.2±12.0	-34.7±0.6
2	4.5±0.0	9	-5.0±0.2	-21.4±0.2	-76.3±1.4	509.7±9.1	-34.1±0.3
3	3.5±0.0	14	-7.0±0.2	-23.0±0.4	-20.1±2.3	521.0±2.5	-32.0±0.4
4	3.5±0.0	22	-1.5±0.4	-21.9±0.2	-85.6±3.4	407.6±10.7	-31.9±0.3
5	0.5±0.1	48	0.1±0.9	-20.3±1.6	-116.7±6.9	449.0±22.8	-31.9±0.7
6	1.7±0.0	8	-3.1±0.2	-21.1±0.8	-63.6±5.0	579.2±9.7	-30.5±0.6
7	0.8±0.0	15	0.0±0.3	-22.1±0.1	-79.4±2.2	484.5±9.9	-30.1±0.3
8	1.4±0.0	6	-3.4±0.5	-20.5±0.4	-57.5±12.7	454.1±16.9	-29.7±0.4
9	1.7±0.0	16	-4.1±0.4	-18.4±0.2	-71.5±3.2	414.6±6.8	-29.6±0.2
10	3.5±0.0	12	-3.0±0.2	-17.2±0.1	-92.4±2.4	407.7±3.6	-29.5±0.3

S stands for cluster size. E_{desolv}, E_{vw} and E_{elec} stand for desolvation, van der Waals and electrostatic energy terms, respectively. A.U. stands for arbitrary units.

2. Experimental methods

No unexpected or unusually high safety hazards were encountered.

2.1. Synthesis

The starting materials, reagents, and solvents were purchased as high-grade commercial products from Sigma-Aldrich (Merck), Acros, ABCR, Fluorochem, and Scharlab. Dichloromethane (DCM), tetrahydrofuran (THF) and diethyl ether were dried using a Pure Solv^{TM} Micro 100 Liter solvent purification system. Chloroform was dried over P_2O_5 and distilled before using. All reactions were carried out under an argon atmosphere in oven-dried glassware unless otherwise stated. MW-assisted reactions were performed in a Biotage Initiator 2.5 reactor.

Analytical thin-layer chromatography (TLC) was run on Merck silica gel plates (Kieselgel 60 F-254), with detection by UV light (λ = 254 nm), 5% ninhydrin solution in ethanol or 10% phosphomolybdic acid solution in ethanol. Unless otherwise stated, products were purified in a Varian 971-FP or Biotage Selekt system with cartridges of silica gel (Varian 50 µM, or Biotage Sfär 60 µM).

All compounds were obtained as oils, except for those whose melting points (mp) are indicated, which were solids. Mp (uncorrected) were determined on a Stuart Scientific electrothermal apparatus. Infrared (IR) spectra were measured on a Bruker Tensor 27 instrument equipped with a Specac ATR accessory of 5200-650 cm^{-1} transmission range; frequencies (ν) are expressed in cm^{-1}. ^{1}H- and ^{13}C-NMR spectra were recorded on a Bruker Avance III 700 MHz (^{1}H, 700 MHz) or Bruker DPX 300 MHz (^{1}H, 300 MHz; ^{13}C, 75 MHz) instrument at rt at the Universidad Complutense de Madrid (UCM) NMR core facility. Bruker DPX 300 MHz equipment was used unless otherwise stated. Chemical shifts (δ) are expressed in parts per million relative to the residual solvent peak for ^{1}H and ^{13}C nucleus ($CDCl_3$: δ_H = 7.26, δ_C = 77.16; methanol-*d4*: δ_H = 3.31, δ_C = 49.00); coupling constants (*J*) are in hertz (Hz). The following abbreviations are used to describe peak patterns when appropriate: s (singlet), d (doublet), t (triplet), m (multiplet), and br (broad). 2D NMR experiments (H,H-COSY, HMQC and HMBC)– of representative compounds were acquired to assign protons and carbons of new structures, and the following abbreviations have been used for the peak assignment: Ar (aryl), carbz (carbazole), cpr (cyclopropane). The relative configuration of the compounds was confirmed by 1D ^{1}H-NMR NOE experiments, in which the signal of interest was irradiated with a selective pulse and NOE interactions with this signal were observed.

Numbered chemical structures for NMR assignation of compounds **16a**, **25a**, **26a**, and **4a** described in the Methods section are shown in Figure S5.

High-resolution mass spectrometry (HRMS) was carried out on a FTMS Bruker APEX Q IV spectrometer in electrospray ionization (ESI) mode at UCM's mass spectrometry facilities.

For all final compounds, a purity of at least 95% was determined by HPLC-MS using an Agilent 1200LC-MSD VL instrument. LC separation was achieved with a Zorbax SB-C3 column (5 μm, 2.1 mm x 50 mm) or an Eclipse XDB-C18 column (5 μm, 4.6 mm x 150 mm), together with a guard column (5 μm, 4.6 mm x 12.5 mm). The mobile phase consisted of A (95:5 water/acetonitrile (ACN)) and B (5:95 water/ACN) with 0.1% ammonium hydroxide and 0.1% formic acid as solvent modifiers, and the gradient is indicated in Table S3. MS analysis was performed with an electrospray irradiation source. The capillary voltage was set to 3.0 kV and the fragmentor voltage to 72 or 35 eV. The drying gas temperature was 350 ºC, the drying gas flow was 10 L/min, and the nebulizer pressure was 20 psi. Spectra were acquired in positive or negative ionization mode from 80 to 800 *m/z* and in UV-mode at four different wavelengths (210, 230, 254, and 280 nm).

Optical rotation [α] was measured on an Anton Paar MCP 100 modular circular polarimeter using a sodium lamp (λ = 589 nm) with a 1 dm path length; concentrations (c) are given as g/100 mL. The er was determined by HPLC using a chiral column (Daicel Chiralpak® IA or IC, 5 μm, 4.6 mm x 150 mm) in reversed- or normal-phase chromatography (detailed conditions are described in Tables S4 and S5). HPLC traces were compared to racemic samples obtained by mixing the enantiomeric compounds independently obtained.

Final compounds **1-3**, **5, 6**, **8**, and **10-13** were characterized (α, R_f, IR, NMR, HPLC-MS) and subsequently transformed into the corresponding hydrochloride salts. Thus, a commercial solution of 2 M HCl(g) in diethyl ether (3 mL/mmol) was added to a solution of the free base in anhydrous DCM or methanol (6 mL/mmol). The resulting salt was isolated by filtration or evaporation of the solvents, washed with anhydrous diethyl ether and dried under vacuum. A purity of at least 95% for the salts was determined by HPLC-MS and elemental chemical analysis (C, H, N, S) using a LECO CHNS-932 instrument at the UCM Microanálisis Elemental core facility.

IUPAC rules have been followed to name all organic compounds, except for (diethoxymethoxy)ethane, *p*-methylbenzenesulfonic acid, *N*,*N*-dimethylpyridin-4-amine, (2*E*)-3-(*p*-methoxyphenyl)prop-2-enal, 1-methoxy-4-[(*E*)-2-nitrovinyl]benzene, 3-

methylbut-2-enal, (*E*)-2-(2-nitrovinyl)phenol, and 4-methoxy-2-[(*E*)-2-nitrovinyl]phenol, whose common names triethyl orthoformate, *p*-toluenesulfonic acid, 4-dimethylaminopyridine (DMAP), *p*-methoxycinnamaldehyde, *trans*-4-methoxy-*β*-nitrostyrene, 3,3-dimethylacrolein, *trans*-2-hydroxy-*β*-nitrostyrene, and *trans*-2-hydroxy-5-methoxy-*β*-nitrostyrene, respectively, have been employed for simplicity.

The following compounds were synthesized as previously described and their spectroscopic data correspond with those reported: *tert*-butyl (2-nitroethyl)carbamate, *tert*-butyl 2-methyl-3-[(1*E*)-3-oxoprop-1-en-1-yl]-1*H*-indole-1-carboxylate, 4-methoxy-2-[(*E*)-2-nitroethenyl]phenol, and 3-aminopyrazine-2-carbaldehyde.

Table S3. Gradient of the mobile phase used in the HPLC-MS analysis of synthetized compounds.

**t (min)**	**%B** [a]
0	0
2	0
10	50
20	100
25	100
30	0

[a] B: 5:95 water/ACN mixture

Table S4. Reversed-phase chiral HPLC conditions.

**Method**	**A**	**B**	**C**	**D**	**E**	**F**	**G**
**Column**	IA	IA	IA	IA	IC	IC	IC
**Eluent** (20 mM NH_4HCO_3 pH 9/ ACN)	40:60	60:40	50:50	40:60	70:30	85:15	30:70
**Flow** (mL/min)	0.6	0.5	0.8	0.8	0.6	0.5	0.6

Table S5. Normal-phase chiral HPLC conditions.

**Method**	**H**	**I**	**J**
**Column**	IA	IA	IC
**Eluent** (hexane/*i*-PrOH)	40:60	30:70 (0.1% EDA)[a]	30:70 (0.1% EDA)[a]
**Flow** (mL/min)	0.6	0.8	0.8

[a] EDA: ethylenediamine

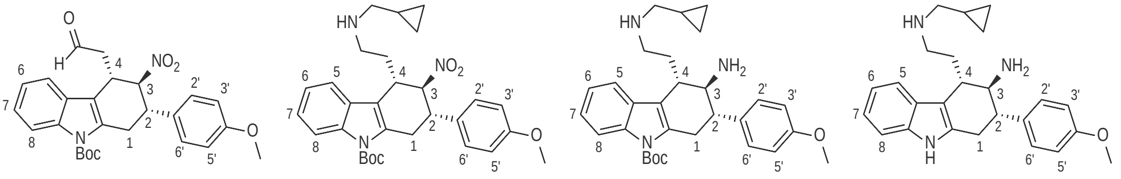

16a 25a 26a 4a

Figure S5. Numbered chemical structures for NMR assignation of compounds **16a, 25a**, **26a**, and **4a**.

General procedure A: Boc deprotection. To a solution of the corresponding *N*-Boc-protected amide or amine (1.00 eq) in anhydrous DCM (5 mL/mmol), TFA (10.0 eq) was added and the reaction was stirred at rt until TLC showed complete consumption of starting material. The corresponding free amide or amine was isolated following the appropriate work-up:

- For amide derivatives (work-up A): The mixture was concentrated and the residue was dissolved in EtOAc and washed with a sat. $NaHCO_3$ solution (x2). The organic layer was dried over Na_2SO_4, filtered and concentrated under reduced pressure to afford the corresponding free amide, which was used in the next step without further purification.
- For amine derivatives (work-up B): The mixture was diluted with DCM and extracted with water (x2). The combined aqueous layers were basified (pH > 10) with a sat. $NaHCO_3$ solution, and extracted with EtOAc (x2). The organic layers were washed with brine, dried over Na_2SO_4 and filtered, and the solvent was evaporated under reduced pressure to afford the corresponding free amine, which was used in the next step without further purification.

General procedure B: one-pot reductive amination. To a solution of the corresponding aldehyde (1.00-1.50 eq) in anhydrous methanol (5 mL/mmol) and DCM (2 mL/mmol; only in those cases where the aldehyde is not soluble in methanol), the appropriate amine (1.00-2.00 eq) was added and the reaction mixture was stirred at rt for 2-4 h to form the corresponding imine (confirmed by ^{1}H-NMR analysis of an aliquot). Then, $NaBH_4$ (2.00 eq) was added at 0 ºC and the mixture was allowed to react at rt for 3 h. The reaction was quenched with a sat. $NaHCO_3$ solution and the solvent was evaporated under reduced pressure. The residue was suspended in water and extracted with EtOAc (x2). The organic layers were washed with brine, dried over Na_2SO_4, filtered and evaporated under reduced pressure. The resulting amine was purified by flash chromatography or used in the next step without further purification.

General procedure C: nitro group reduction. To a solution of the corresponding nitro derivative (1.00 eq) in a 1:1 mixture of glacial acetic acid and anhydrous methanol (4 mL/mol), Zn powder (10.0 eq) was added and the reaction was stirred until complete conversion of starting material (1-3 h). Then, the mixture was filtered and washed with methanol, and the filtrate was evaporated. The residue was suspended in a sat. $NaHCO_3$ solution and extracted with DCM (x3). The combined organic layers were washed with brine, dried over Na_2SO_4, filtered and concentrated under reduced pressure. The resulting amine was purified by flash chromatography.

General procedure D: Pinnick oxidation. To a solution of the corresponding aldehyde (1.00 eq) in *tert*-butanol (5 mL/mmol) and anhydrous THF (1 mL/mmol), 2-methylbut-2-ene (1.20 eq), sodium chlorite (1.20 eq) and a sat. solution of K_2HPO_4 (0.4 mL/mmol) were added successively. The mixture was stirred at 30 ºC overnight. The reaction was then quenched with water, and a sat. $NaHCO_3$ solution was added until pH 9. After stirring the mixture for 30 min, the organic layer was discarded. The aqueous phase was treated with 1 M HCl until pH 2, stirred for 30 min and extracted with EtOAc (x2). The combined organic layers were washed with brine, dried over Na_2SO_4, filtered and concentrated under vacuum to afford the desired carboxylic acid, which was used in the next step without further purification.

General procedure E: one-pot synthesis of amides via acid chloride. To a solution of the corresponding carboxylic acid (1.00 eq) in anhydrous DCM (15 mL/mmol) at 0 ºC, oxalyl chloride (1.00 eq, 2 M in DCM) and DMF (cat.) were added, and the solution was stirred at rt for 1 h. Then, (cyclopropylmethyl)amine (1.50 eq) was added at 0 ºC, and the reaction was allowed to warm up to rt and stirred until complete conversion of starting material (3-18 h). Next, the mixture was washed with a sat. $NaHCO_3$ solution (x2) and a 1:1 mixture of water/brine (x4). The organic layer was dried over Na_2SO_4 and filtered, and concentrated under vacuum to afford the desired amide, which was used in the next step without further purification.

Synthesis of scaffolds 14, 15, 17-21 and structural data

***tert*-Butyl 4-(4-methoxyphenyl)-5-nitro-2-oxopiperidine-1-carboxylate, 14.** A previously reported procedure[1] was applied for the synthesis of enantiomers (4*S*,5*S*) and (4*R*,5*R*) of compound **14,** using the appropriate starting materials and the (*S*) or (*R*) enantiomer of 2-{diphenyl[(trimethylsilyl)oxy]methyl}pyrrolidine as catalyst.

(4*S*,5*S*)-**14** (**14a**) was obtained from *p*-methoxycinnamaldehyde (212 mg, 1.31 mmol) and *tert*-butyl (2-nitroethyl)carbamate (374 mg, 1.96 mmol), using the *S* enantiomer of the catalyst (40 mg, 0.13 mmol), as an oil (205 mg, 45%, dr = 9:1). Chromatography: hexane to hexane/EtOAc 85:15.

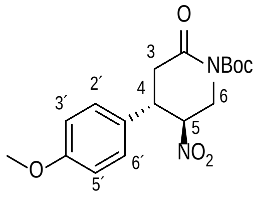

R_f: 0.37 (hexane/EtOAc 7:3). IR (ATR): ν 1775 (C=O), 1720 (C=O), 1252 (COC). ^{1}H-NMR ($CDCl_3$, major diastereoisomer): δ 1.55 (s, 9H, $3CH_3$), 2.76 (dd, *J* = 16.7, 10.6, 1H, H_3), 2.89 (dd, *J* = 16.7, 5.8, 1H, H_3), 3.79-3.82 (m, 1H, H_4), 3.80 (s, 3H, OCH_3), 4.00 (dd, *J* = 14.5, 4.9, 1H, H_6), 4.56 (dd, *J* = 14.5, 5.1, 1H, H_6), 4.89 (dt, *J* = 7.5, 5.0, 1H, H_5), 6.90 (d, *J* = 8.7, 2H, $H_{3'}$, $H_{5'}$), 7.13 (d, *J* = 8.7, 2H, $H_{2'}$, $H_{6'}$). ^{13}C-NMR ($CDCl_3$, major diastereoisomer): δ 28.1 ($3CH_3$), 39.6 (C_3), 41.3 (C_4), 46.1 (C_6), 55.7 (OCH_3), 84.5 ($\underline{C}(CH_3)_3$), 86.0 (C_5), 114.9 ($C_{3'}$, $C_{5'}$), 128.1 ($C_{2'}$, $C_{6'}$), 130.6 ($C_{1'}$), 151.5 (NCOO), 159.6 ($C_{4'}$), 168.4 (C_2). 1D ^{1}H-NMR NOE: irradiation of the signal at δ 4.00 ppm (dd, H_6) yielded NOE on 3.77-3.86 (m, H_4); and irradiation of the signal at δ 4.56 ppm (dd, H_6) yielded NOE on 4.89 (dt, H_5), and 7.13 (d, $H_{2'}$, $H_{6'}$). HPLC (t_R, min): 17.34. MS (ESI, *m/z*, %): 351.2 ($[M+H]^+$, 100).

(4*R*,5*R*)-**14** (**14b**) was obtained from 4-methoxycinnamaldehyde (240 mg, 1.48 mmol) and *tert*-butyl (2-nitroethyl)carbamate (422 mg, 2.22 mmol), using the *R* enantiomer of the catalyst (46 mg, 0.15 mmol), as an oil (217 mg, 58%, dr = 9:1). Chromatography: hexane to hexane/EtOAc 85:15.

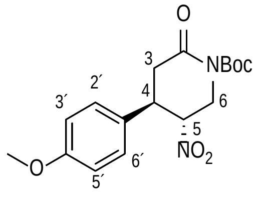

Spectroscopic data were in agreement with those described for enantiomer **14a**.

***tert*-Butyl 4-(4-methoxyphenyl)-3-nitro-3,4-dihydropyridine-1(2*H*)-carboxylate, 15.** A previously reported procedure[1] was applied for the synthesis of enantiomers (4*S*,5*S*)- and (4*R*,5*R*) of compound **15**, using the appropriate starting materials and the (*S*) or (*R*) enantiomer of 2-{diphenyl[(trimethylsilyl)oxy]methyl}pyrrolidine as catalyst.

(3*S*,4*S*)-**15** (**15a**) was obtained from 4-methoxycinnamaldehyde (150 mg, 0.92 mmol) and *tert*-butyl (2-nitroethyl)carbamate (262 mg, 1.38 mmol), using the *S* enantiomer of the catalyst (30 mg, 0.09 mmol), as an oil (270 mg, 87%, dr = 8:2). Chromatography: hexane.

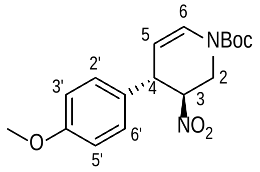

R_f: 0.60 (hexane/EtOAc 8:2). IR (ATR): ν 1709 (C=O), 1550 (NO_2), 1253 (COC). ^{1}H-NMR ($CDCl_3$, major diastereoisomer): δ 1.52 (s, 9H, $3CH_3$), 3.79 (s, 3H, OCH_3), 3.89-4.17 (m, 3H, $2H_2$, H_4), 4.61 (br s, H_3), 4.83-4.95 (m, H_5), 6.83 and 7.08 (d, *J* = 8.9, and d, *J* = 8.7, 1H, H_6, amide rotamers), 6.87 (d, *J* = 8.7, 2H, $H_{3'}$, $H_{5'}$), 7.15 (d, *J* = 8.7, 2H, $H_{2'}$, $H_{6'}$). ^{13}C-NMR ($CDCl_3$, major diastereoisomer): δ 28.3 ($3CH_3$), 41.7 (C_2, C_4), 55.34 (OCH_3), 82.4 ($\underline{C}(CH_3)_3$), 85.3 (C_3), 104.6 (C_5), 128.2 and 130.34 (C_6 amide rotamers), 114.5 ($C_{3'}$, $C_{5'}$), 129.2 ($C_{2'}$, $C_{6'}$), 131.8 ($C_{1'}$), 149.4 (NCOO), 159.7 ($C_{4'}$). HPLC (t_R, min): 23.6. MS (ESI, *m/z*, %): 235.1 ([M-Boc+2H]$^+$, 100).

(3*R*,4*R*)-**15** (**15b**) was obtained from 4-methoxycinnamaldehyde (130 mg, 0.80 mmol) and *tert*-butyl (2-nitroethyl)carbamate (228 mg, 1.20 mmol), using the *R* enantiomer of the catalyst (26 mg, 0.08 mmol), as an oil (226 mg, 84%, dr = 8:2). Chromatography: hexane.

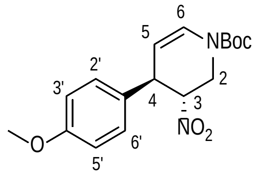

Spectroscopic data were in agreement with those described for enantiomer **15a**.

6,9-bis(4-Methoxyphenyl)-10-nitro-6a,9,10,10a-tetrahydro-6*H*-benzo[*c*]chromene-8-carbaldehyde, 17 and 2-(4-methoxyphenyl)-4-(nitromethyl)-3,4-dihydro-2*H*-1-benzopyran-3-carbaldehyde, 19. To a solution of (*S*)- or (*R*)-2-{diphenyl[(trimethylsilyl)oxy]methyl}pyrrolidine (0.20 eq), *p*-methoxycinnamaldehyde (1.20 eq) and acetic acid (0.2 eq) in anhydrous chloroform (16.6 mL/mmol), *trans*-2-hydroxy-*β*-nitrostyrene (1 eq) was added and the resulting solution was stirred at 25 ºC overnight. Then, the solvent was evaporated under reduced pressure and the residue was purified by flash chromatography (hexane to hexane/EtOAc 8:2) to afford (6*S*,6a*S*,9*R*,10*R*,10a*S*)- or (6*R*,6a*R*,9*S*,10*S*,10a*R*)-**17** and (2*S*,3*S*,4*S*)- or (2*R*,3*R*,4*R*)-**19**, respectively.

(6*S*,6a*S*,9*R*,10*R*,10a*S*)-**17** (**17a**) and (2*S*,3*S*,4*S*)-**19** (**19a**) and were obtained from *trans*-2-hydroxy-*β*-nitrostyrene (50 mg, 0.30 mmol), using the *S* enantiomer of the catalyst (19.7 mg, 0.06 mmol), as yellow solids in 21% (30 mg) and 31% (31 mg) yields, respectively.

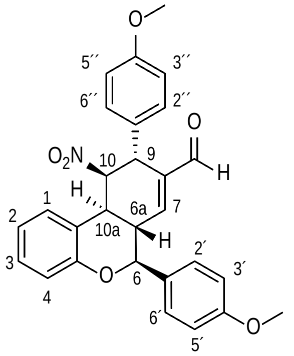

17a: M.p.: 175-177 ºC (lit.[2]) 176-179 ºC). R_f: 0.42 (hexane/EtOAc 7:3). $[\alpha]^D_{20}$ = +12.0 (c = 0.29, $CHCl_3$) (lit.[2] $[\alpha]^D_{25}$: +17.5 (c = 0.30, $CHCl_3$)). ^{1}H-NMR ($CDCl_3$): δ 3.33-3.44 (m, 1H, H_{6a}), 3.58 (dd, *J* = 11.4, 2.2, 1H, H_{10a}), 3.80 (s, 3H, CH_3), 3.89 (s, 3H, CH_3), 4.60 (s, 1H, H_9), 5.07 (d, *J* = 10.1, 1H, H_6), 5.45 (d, *J* = 2.2, 1H, H_{10}), 6.61 (d, *J* = 1.4, 1H, H_7), 6.87-6.92 (m, 4H, $H_{3''}$, $H_{5''}$, H_2, H_4), 7.06 (d, *J* = 8.7, 2H, $H_{3'}$, $H_{5'}$), 7.13-7.16 (m, 4H, $H_{2''}$, $H_{6''}$, H_1, H_3), 7.48 (d, *J* = 8.7, 2H, $H_{2'}$, $H_{6'}$), 9.37 (s, 1H, CHO). 1D ^{1}H-NMR NOE: irradiation of the signal at δ 5.07 ppm (d, H_6) yielded NOE on 3.58 (dd, H_{10a}); irradiation of the signal at δ 3.58 ppm (dd, H_{10a}) yielded NOE on 5.07 (d, H_6), 5.45 (d, H_{10}), and 7.13-7.16 (m, $H_{2''}$, $H_{6''}$); irradiation of the signal at δ 5.45 ppm (dd, H_{10}) yielded NOE on 3.58 (dd, H_{10a}), and 7.13-7.16 (m, $H_{2''}$, $H_{6''}$); and irradiation of the signal at δ 3.33-3.44 ppm (m, H_{6a}) yielded NOE on 7.48 (d, $H_{2'}$, $H_{6'}$). HPLC (t_R, min): 23.48. MS (ESI, *m/z*, %): 472.1 ($[M+H]^+$, 100). The spectroscopic data were consistent with those previously reported.[2]

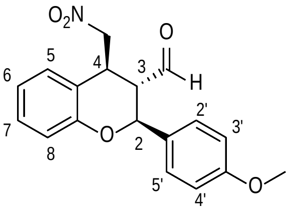

19a: M.p.: 110-112 ºC. R_f: 0.67 (hexane/EtOAc 7:3). $[\alpha]^D_{20}$ = -32.20 (c = 1.06, $CHCl_3$). IR (ATR): ν 1724 (C=O), 1585 (NO_2), 1247 (COC). ^{1}H-NMR ($CDCl_3$): δ 3.58 (td, *J* = 8.1, 1.0, 1H, H_3), 3.83 (s, 3H, CH_3), 4.20-4.30 (m, 1H, H_4), 4.50 (dd, *J* = 13.1, 6.9, 1H, ½CH_2NO_2), 4.59 (dd, *J* = 13.1, 4.6, 1H, ½CH_2NO_2), 5.07 (d, *J* = 8.1, 1H, H_2), 6.94-7.04 (m, 2H, H_6, H_8), 6.96 (d, *J* = 8.7, 2H, $H_{3'}$, $H_{5'}$), 7.22 (t, *J* = 7.5, 2H, H_5, H_7), 7.35 (d, *J* = 8.7, 2H, $H_{2'}$, $H_{6'}$), 9.49 (d, *J* = 1.0, 1H, CHO). ^{13}C-NMR ($CDCl_3$): δ 33.7 (C_4), 54.2 (C_3),

55.5 (CH_3), 76.7 (C_2), 77.8 (CH_2NO_2), 114.8 ($C_{3'}$, $C_{5'}$), 118.1 (C_8), 119.1 (C_{4a}), 122.3 (C_6), 127.9 (C_5), 128.1 ($C_{2'}$, $C_{6'}$), 129.26 (C_7), 129.30 ($C_{1'}$), 154.9 (C_{8a}), 160.3 ($C_{4'}$), 199.9 (CHO). 1D ^{1}H-NMR NOE: irradiation of the signal at δ 4.20-4.30 ppm (m, H_4) yielded NOE on 5.07 (d, H_2), 7.22 (d, H_5), and 9.49 (d, CHO). HPLC (t_R, min): 19.86. MS (ESI, *m/z*, %): 328.1 ($[M+H]^+$, 100).

(6*R*,6a*R*,9*S*,10*S*,10a*R*)-**17** (**17b**) and (2*R*,3*R*,4*R*)-**19** (**19b**) were obtained from *trans*-2-hydroxy-*β*-nitrostyrene (50 mg, 0.30 mmol), using the *R* enantiomer of the catalyst (19.7 mg, 0.06 mmol), as yellow solids in 26% (37 mg) and 22% (22 mg) yields, respectively.

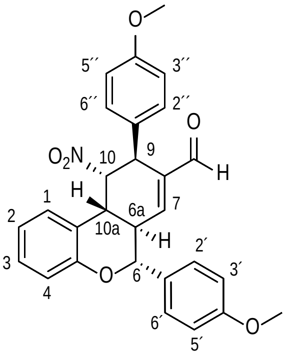

17b: $[\alpha]^D_{20}$= -10.1 (c = 0.28, $CHCl_3$). Spectroscopic data were in agreement with those described for enantiomer **17a**.

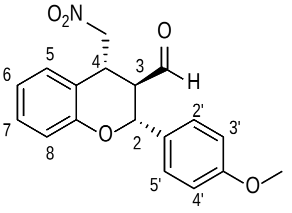

19b: $[\alpha]^D_{20}$= +34.60 (c = 1.13, $CHCl_3$). Spectroscopic data were in agreement with those described for enantiomer **19a**.

9-(4-Methoxyphenyl)-6,6-dimethyl-10-nitro-6a,9,10,10a-tetrahydro-6*H*-dibenzo[*b*,*d*]pyran-8-carbaldehyde, 18. A previously reported procedure[2] was applied for the synthesis of enantiomers (6a*S*,9*R*,10*R*,10a*S*) and (6a*R*,9*S*,10*S*,10a*R*) of compound **18**, using the appropriate starting materials and the (*S*) or (*R*) enantiomer of 2-{diphenyl[(trimethylsilyl)oxy]methyl}pyrrolidine as catalyst.

(6a*S*,9*R*,10*R*,10a*S*)-**18** (**18a**) was obtained from 3,3-dimethylacrolein (42 µL, 0.44 mmol), *trans*-2-hydroxy-β-nitrostyrene (100 mg, 0.40 mmol) and *p*-methoxycinnamaldehyde (78 mg, 0.48 mmol), using the *S* enantiomer of the catalyst (22 mg, 0.08 mmol), as a yellow oil (80 mg, 48%). Chromatography: hexane to hexane/EtOAc 85:15.

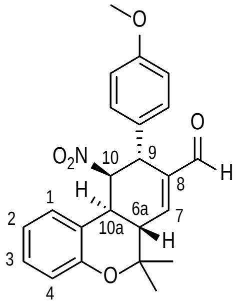

R_f: 0.35 (hexane/EtOAc 7:3). $[\alpha]^D_{20}$ = -86.8 (c = 2.00, $CHCl_3$) (lit.[2] $[\alpha]^D_{20}$ = -83.1 (c = 2.00, $CHCl_3$). IR (ATR): ν 1691 (C=O), 1546 (NO_2), 1489 (C-N), 1253 (COC). ^{1}H-NMR ($CDCl_3$): δ 1.31 (s, 3H, CH_3), 1.76 (s, 3H, CH_3), 3.06 (d, *J* = 12.2, 1H, H_{6a}), 3.35 (dd, *J* = 12.2, 1H, H_{10a}), 3.79 (s, 3H, OCH_3), 4.60 (s, 1H, H_9), 5.47 (s, 1H, H_{10}), 6.81-6.90 (m, 4H, H_7, $3CH_{Ar}$), 7.11-7.17 (m, 5H, $5CH_{Ar}$), 9.60 (s, 1H, CHO). The spectroscopic data were consistent with those previously reported.[2]

(6a*R*,9*S*,10*S*,10a*R*)-**18** (**18b**) was obtained from 3,3-dimethylacrolein (76 µL, 0.79 mmol), *trans*-2-hydroxy-β-nitrostyrene (180 mg, 0.72 mmol) and *p*-methoxycinnamaldehyde (140 mg, 0.86 mmol), using the *R* enantiomer of the catalyst (47 mg, 0.14 mmol), as a yellow oil (122 mg, 43%). Chromatography: hexane to hexane/EtOAc 85:15. $[\alpha]^D_{20}$= +86.1 (c = 1.98, $CHCl_3$).

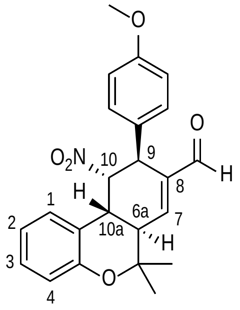

Spectroscopic data were in agreement with those described for enantiomer **18a**.

6-Methoxy-2,2-dimethyl-4-(nitromethyl)-3,4-dihydro-2*H*-1-benzopyran-3-carbaldehyde, 20. A previously reported procedure[3] was applied for the synthesis of enantiomers (3*S*,4*S*) and (3*R*,4*R*) of compound **20**, using the appropriate starting materials and the (*S*) or (*R*) enantiomer of 2-{diphenyl[(trimethylsilyl)oxy]methyl}pyrrolidine as catalyst.

(3*S*,4*S*)-**20** (**20a**) was obtained from 3,3-dimethylacrolein (0.14 mL, 1.50 mmol) and 4-methoxy-2-[(*E*)-2-nitroethenyl]phenol (196 mg, 1.00 mmol), using the *S* enantiomer of the catalyst (65 mg, 0.20 mmol), as a yellow oil (170 mg, 61%, dr = 8:2). Chromatography: hexane to hexane/EtOAc 9:1.

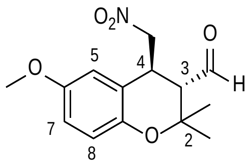

R_f: 0.49 (hexane/EtOAc 7:3). IR (ATR): ν 1720 (C=O), 1551 (NO_2), 1238 (COC). ^{1}H-NMR ($CDCl_3$, major diastereoisomer): δ 1.12 (CH_3), 1.67 (CH_3), 3.21 (dd, *J* = 10.4, 1.3, 1H, H_3), 3.75 (OCH_3), 3.98 (dt, *J* = 10.3, 5.1, 1H, H_4), 4.65 (dd, *J* = 13.3, 4.6, 1H, ½CH_2NO_2), 4.72 (dd, *J* = 13.3, 6.0, 1H, ½CH_2NO_2), 6.72-6.73 (m, 1H, H_5), 6.76-6.81 (m, 2H, H_7, H_8), 9.89 (d, *J* = 1.6, 1H, CHO). ^{13}C-NMR ($CDCl_3$, major diastereoisomer): δ 21.3 (CH_3), 28.5 (CH_3), 31.7 (C_4), 55.8 (OCH_3), 57.7 (C_3), 74.4 (C_2), 78.1 (CH_2NO_2), 111.9 (C_5), 115.1, 119.2 (C_7, C_8), 119.7 (C_{4a}), 146.7 (C_{8a}), 154.2 (C_6), 200.3 (CHO). 1D ^{1}H-NMR NOE: irradiation of the signal at δ 3.21 ppm (dd, H_3) yielded NOE on 1.67 (s, CH_3), 4.65 (dd, ½CH_2NO_2), and 4.72 (dd, ½CH_2NO_2); and irradiation of the signal at δ 3.98 ppm (dt, H_4) yielded NOE on 1.12 (s, CH_3). HPLC (t_R, min): 18.80. MS (ESI, *m/z*, %): 297.2 ($[M+NH_4]^+$, 100).

(3*R*,4*R*)-**20** (**20b**) was obtained from 3,3-dimethylacrolein (0.24 mL, 2.51 mmol) and 4-methoxy-2-[(*E*)-2-nitroethenyl]phenol (325 mg, 1.67 mmol), using the *R* enantiomer of the catalyst (108 mg, 0.33 mmol), as a yellow oil (242 mg, 52%, dr = 8:2). Chromatography: hexane to hexane/EtOAc 9:1.

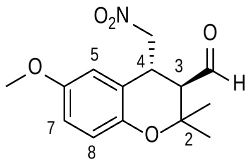

Spectroscopic data were in agreement with those described for enantiomer **20a**.

6-(4-Methoxyphenyl)-5,6-dihydropyrido[2,3-*b*]pyrazine-7-carbaldehyde, 21. To a solution of (*S*)- or (*R*)-2-{diphenyl[(trimethylsilyl)oxy]methyl}pyrrolidine (0.20 eq), *p*-methoxycinnamaldehyde (1.50 eq), and acetic acid (0.20 eq) in anhydrous chloroform (1mL/mmol), 3-aminopyrazine-2-carbaldehyde (1.00 eq) was added and the reaction was stirred at rt for 7 days. Next, the solvent was evaporated under reduced pressure and the residue was purified by flash chromatography (hexane to hexane/EtOAc 1:1) affording intermediate (*R*)- or (*S*)-**21**.

(*R*)-**21** (**21a**) was obtained from 3-aminopyrazine-2-carbaldehyde (50 mg, 0.41 mmol), using the *S* enantiomer of the catalyst (26 mg, 0.08 mmol), as a yellow oil (44 mg, 40%).

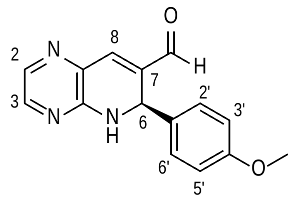

R_f: 0.25 (hexane/EtOAc 4:6). $[\alpha]^D{}_{20}$ = -255.3 (c = 0.53, $CHCl_3$). IR (ATR): ν 3236 (NH), 1671 (C=O), 1246 (COC). ^{1}H-NMR ($CDCl_3$): δ 3.76 (s, 3H, CH_3), 5.80 (s, 1H, H_6), 5.97 (br s, 1H, NH), 6.82 (d, *J* = 8.7, 2H, $H_{3'}$, $H_{5'}$), 7.26 (d, *J* = 8.7, 2H, $H_{2'}$, $H_{6'}$), 7.32 (s, 1H, H_8), 7.80 (d, *J* = 2.7, 1H, H_3), 7.85 (d, *J* = 2.6, 1H, H_2), 9.56 (s, 1H, CHO). ^{13}C-NMR ($CDCl_3$): δ 54.5 (C_6), 55.4 (CH_3), 114.3 ($C_{3'}$, $C_{5'}$), 127.8 ($C_{2'}$, $C_{6'}$), 133.4 (C_{8a}), 135.0 (C_2), 135.4 ($C_{1'}$), 138.4 (C_7), 140.8 (C_8), 144.8 (C_3), 153.2 (C_{4a}), 159.8 ($C_{4'}$), 190.4 (CHO).

(*S*)-**21** (**21b**) was obtained from 3-aminopyrazine-2-carbaldehyde (50 mg, 0.41 mmol), using the *R* enantiomer of the catalyst (26 mg, 0.08 mmol), as a yellow oil (49 mg, 45%).

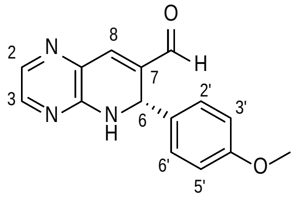

$[\alpha]^D{}_{20}$= +260.9 (c = 0.55, $CHCl_3$). Spectroscopic data were in agreement with those described for enantiomer **21a**.

Synthesis of final compounds 1-3, 5-13 and UCM-13369-Cy5, and structural data

***tert*-Butyl [4-(4-methoxyphenyl)-6-oxopiperidin-3-yl]carbamate, 22.** (a) Deprotection of the amido group: Following the general procedure A (work-up A), the corresponding enantiomer of **14** was transformed into (4*S*,5*S*)- or (4*R*,5R)-4-(4-methoxyphenyl)-5-nitropiperidin-2-one which was directly used in the next reaction.

(4*S*,5*S*)-4-(4-Methoxyphenyl)-5-nitropiperidin-2-one was obtained from **14a** (112 mg, 0.32 mmol) as an oil (68 mg, 85%, dr = 85:15).

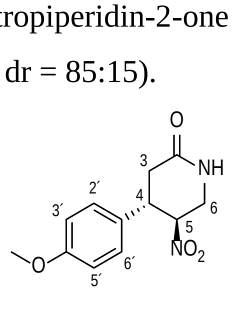

R_f: 0.17 (EtOAc). IR (ATR): ν 3226 (NH), 1672 (C=O), 1522 (NO_2), 1252 (COC). ^{1}H-NMR ($CDCl_3$, major diastereoisomer): δ 2.67 (dd, *J* = 18.1, 8.5, 1H, H_3), 2.82 (dd, *J* = 18.1, 6.3, 1H, H_3), 3.72 (ddd, *J* = 12.9, 4.9, 2.7, 1H, H_6), 3.80 (s, 3H, CH_3), 3.82-3.92 (m,

1H, H_4), 3.95 (ddd, J = 12.8, 6.9, 1.9, 1H, H_6), 4.87 (ddd, J = 8.1, 6.9, 4.9, 1H, H_5), 6.56 (s, 1H, NH), 6.89 (d, J = 8.7, 2H, $H_{3'}$, $H_{5'}$), 7.16 (d, J = 8.7, 2H, $H_{2'}$, $H_{6'}$). ^{13}C-NMR ($CDCl_3$, major diastereoisomer): δ 35.3 (C_3), 41.5 (C_4), 43.1 (C_6), 55.5 (CH_3), 84.6 (C_5), 114.9 ($C_{3'}$, $C_{5'}$), 128.3 ($C_{2'}$, $C_{6'}$), 130.0 ($C_{1'}$), 159.7 ($C_{4'}$), 170.2 (C_2). 1D ^{1}H-NMR NOE: irradiation of the signal at δ 4.87 ppm (ddd, H_5) yielded NOE on 7.16 (d, $H_{2'}$, $H_{6'}$). HPLC (t_R, min): 14.25. MS (ESI, *m/z*, %): 251.1 ($[M+H]^+$, 100).

(4*R*,5*R*)-4-(4-Methoxyphenyl)-5-nitropiperidin-2-one was obtained from **14b** (65 mg, 0.19 mmol) as an oil (43 mg, 93%, dr = 85:15).

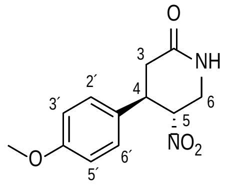

Spectroscopic data were in agreement with those described for the (4*S*,5*S*) enantiomer.

(b) Reduction of the nitro group and *in situ* protection: To a solution of the corresponding enantiomer of 4-(4-methoxyphenyl)-5-nitropiperidin-2-one described above (1.00 eq) in anhydrous methanol (7 mL/mmol) at 0 ºC, nickel chloride hexahydrate (0.05 eq) was added and the reaction was stirred at this temperature for 5 min. $NaBH_4$ (4.00 eq) was added portionwise over 30 min and the reaction was stirred at 0 ºC for 30 min. Then, di-*tert*-butyl dicarbonate (1.20 eq) was added and the reaction was allowed to warm up to rt and stirred overnight. Next, the reaction was quenched with a sat. NH_4Cl solution, the mixture was filtered through celite, and the filtrate was evaporated. The residue was dissolved in EtOAc and washed with water, a sat. $NaHCO_3$ solution and brine. The organic layer was dried over Na_2SO_4, filtered, and evaporated under reduced pressure to afford title compound (3*S*,4*S*)- or (3*R*,4*R*)-**22**, which was used in the next step without further purification.

(3*S*,4*S*)-22 (22a) was obtained from (4*S*,5*S*)-4-(4-methoxyphenyl)-5-nitropiperidin-2-one (102 mg, 0.27 mmol) as an oil (130 mg, quantitative, dr = 85:15).

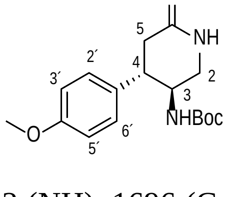

R_f: 0.14 (EtOAc). IR (ATR): ν 3292 (NH), 1696 (C=O), 1656 (C=O), 1212 (COC). ^{1}H-NMR ($CDCl_3$, major diastereoisomer): δ 1.36 (s, 9H, $3CH_3$), 2.59 (dd, J = 18.0, 9.1, 1H, H_5), 2.74 (dd, J = 18.1, 5.9, 1H, H_5), 3.07-3.17 (m, 2H, H_2, H_4), 3.58-3.63 (m, 1H, H_2),

3.80 (s, 3H, OCH_3), 4.02 (br s, 1H, H_3), 4.56 (d, *J* = 7.2, 1H, NHBoc), 6.14 (br s, 1H, NH), 6.88 (d, *J* = 8.6, 2H, $H_{3'}$, $H_{5'}$), 7.14 (d, *J* = 8.5, 2H, $H_{2'}$, $H_{6'}$). ^{13}C-NMR ($CDCl_3$, major diastereoisomer): δ 28.4 ($3CH_3$), 37.3 (C_5), 42.7 (C_4), 45.8 (C_2), 55.4 (OCH_3), 80.2 ($C(CH_3)_3$), 114.5 ($C_{3'}$, $C_{5'}$), 128.5 ($C_{2'}$, $C_{6'}$), 132.3 ($C_{1'}$), 155.4 (NHCOO), 159.0 ($C_{4'}$), C_3 and C_6 not observed. HPLC (t_R, min): 14.95. MS (ESI, *m/z*, %): 265.1 ($[M-O(CH_3)_3+NH_4]^+$, 100).

(3*R*,4*R*)-22 (22b) was obtained from (4*R*,5*R*)-4-(4-methoxyphenyl)-5-nitropiperidin-2-one (43 mg, 0.17 mmol) as an oil (54 mg, quantitative, dr = 85:15).

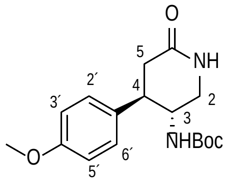

Spectroscopic data were in agreement with those described for enantiomer **22a**.

5-Amino-1-(cyclopropylmethyl)-4-(4-methoxyphenyl)piperidin-2-one, 1. (a) Alkylation of the amido group: To a solution of enantiomer **22a** or **22b** (1.00 eq) in anhydrous DMF (8 mL/mmol) at 0 ºC, NaH (1.50 eq) was added and the reaction was stirred at this temperature for 1 h. Then, (bromomethyl)cyclopropane (2.00 eq) and NaI (2.00 eq) were added and the mixture was stirred at rt overnight. Next, the mixture was diluted with EtOAc, and washed with water (x2) and a 1:1 mixture of water/brine (x3). The organic layer was dried over Na_2SO_4, filtered and the solvent was evaporated under reduced pressure. The residue was purified by flash chromatography (hexane to hexane/EtOAc 1:1) to yield title compound (3*S*,4*S*)- or (3*R*,4*R*)-*tert*-butyl [1-(cyclopropylmethyl)4-(4-methoxyphenyl)-6-oxopiperidin-3-yl]carbamate, as a single diastereoisomer.

(3*S*,4*S*)-*tert*-Butyl [1-(cyclopropylmethyl)4-(4-methoxyphenyl)-6-oxopiperidin-3-yl]carbamate was obtained from **22a** (160 mg, 0.50 mmol) as an oil (78 mg, 42%).

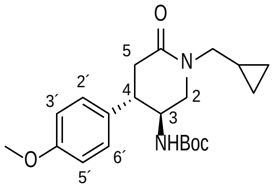

R_f: 0.28 (hexane/EtOAc 4:6). $[\alpha]^D_{20}$ = -10.8 (c = 0.52, $CHCl_3$). IR (ATR): ν 1709 (C=O), 1630 (C=O), 1252 (COC). ^{1}H-NMR ($CDCl_3$): δ 0.20-0.31 (m, 2H, CH_{2cpr}), 0.46-0.57 (m, 2H, CH_{2cpr}), 0.93-1.06 (m, 1H, CH_{cpr}), 1.36 (s, 9H, $3CH_3$), 2.60 (dd, *J* = 17.9, 9.5, 1H, H_5), 2.76 (dd, *J* = 17.9, 5.9, 1H, H_5), 3.06 (td, *J* = 9.3, 6.0, 1H, H_4), 3.17-3.23 (m, 2H, H_2,

½NCH_2), 3.37 (dd, *J* = 13.9, 6.9, 1H, ½NCH_2), 3.67 (dd, *J* = 12.2, 4.9, 1H, H_2), 3.79 (s, 3H, OCH_3), 4.05 (br s, 1H, H_3), 4.49 (br s, NH), 6.87 (d, *J* = 8.7, 2H, $H_{3'}$, $H_{5'}$), 7.12 (d, *J* = 8.7, 2H, $H_{2'}$, $H_{6'}$). ^{13}C-NMR ($CDCl_3$): δ 3.5 (CH_{2cpr}), 3.8 (CH_{2cpr}), 9.2 (CH_{cpr}), 28.4 ($3CH_3$), 38.1 (C_5), 43.5 (C_4), 51.25, 51.28 (C_2, NCH_2), 55.4 (OCH_3), 80.1 ($\underline{C}(CH_3)_3$), 114.5 ($C_{3'}$, $C_{5'}$), 128.5 ($C_{2'}$, $C_{6'}$), 132.4 ($C_{1'}$), 155.4 (NHCOO), 159.0 ($C_{4'}$), 168.4 (C_6), C_3 not observed. 1D ^{1}H-NMR NOE: irradiation of the signal at δ 4.05 ppm (br s, H_3) yielded NOE on 7.12 (d, $H_{2'}$, $H_{6'}$). HPLC (t_R, min): 18.50. MS (ESI, *m/z*, %): 375.2 ($[M+H]^+$, 100).

(3*R*,4*R*)-*tert*-Butyl [1-(cyclopropylmethyl)4-(4-methoxyphenyl)-6-oxopiperidin-3-yl]carbamate was obtained from **22b** (120 mg, 0.37 mmol) as an oil (50 mg, 36%).

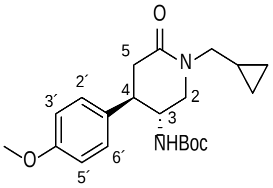

$[\alpha]^D_{20}$= +12.2 (c = 0.44, $CHCl_3$). Spectroscopic data were in agreement with those described for the (3*S*,4*S*) enantiomer.

(b) Deprotection of the amino group: Following the general procedure A (work-up B), the corresponding enantiomer of *tert*-butyl [1-(cyclopropylmethyl)4-(4-methoxyphenyl)-6-oxopiperidin-3-yl]carbamate described above was transformed into final compound (4*S*,5*S*)- or (4*R*,5R)-**1**.

(4*S*,5*S*)-1 (1a) was obtained from (3*S*,4*S*)-*tert*-butyl [1-(cyclopropylmethyl)4-(4-methoxyphenyl)-6-oxopiperidin-3-yl]carbamate (52 mg, 0.16 mmol) as an oil (28 mg, 87%, er = 97:3).

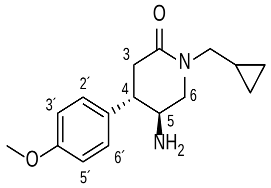

R_f: 0.28 (DCM/methanol 8:2). $[\alpha]^D_{20}$ = -9.1 (c = 0.11, $CHCl_3$). Chiral HPLC (method A, t_R, min): 7.55. IR (ATR): ν 3416 (NH), 1633 (C=O), 1252 (COC). ^{1}H-NMR ($CDCl_3$): δ 0.24-0.29 (m, 2H, CH_{2cpr}), 0.50-0.56 (m, 2H, CH_{2cpr}), 0.94-1.06 (m, 1H, CH_{cpr}), 2.55 (dd, *J* = 17.5, 11.4, 1H, H_3), 2.74 (dd, *J* = 17.4, 5.4, 1H, H_3), 2.75-2.86 (m, 1H, H_4), 3.18-3.34 (m, 2H, H_5, H_6), 3.21 (dd, *J* = 14.0, 7.2, 1H, ½NCH_2), 3.41 (dd, *J* = 13.9, 7.0, 1H, ½NCH_2), 3.54-3.60 (m, 1H, H_6), 3.80 (s, 3H, CH_3), 6.90 (d, *J* = 8.7, 2H, $H_{3'}$, $H_{5'}$), 7.14 (d, *J* = 8.7, 2H, $H_{2'}$, $H_{6'}$). ^{13}C-NMR ($CDCl_3$): δ 3.6 (CH_{2cpr}), 3.8 (CH_{2cpr}), 9.3 (CH_{cpr}), 38.8

(C_3), 47.0 (br s, C_4), 51.2 (NCH_2), 51.4 (C_5), 54.3 (br s, C_6), 55.5 (CH_3), 114.7 ($C_{3'}$, $C_{5'}$), 128.7 ($C_{2'}$, $C_{6'}$), 132.8 ($C_{1'}$), 159.0 ($C_{4'}$), 168.9 (C_2). HPLC (t_R, min): 3.70. MS (ESI, *m/z*, %): 275.2 ($[M+H]^+$, 100). Elemental analysis calculated for $C_{16}H_{22}N_2O_2$·HCl: %C 61.83, %H 7.46, %N 9.01; experimental: %C 61.51, %H 7.16, %N 8.89.

(4*R*,5*R*)-1 (1b) was obtained from (3*R*,4*R*)-*tert*-butyl [1-(cyclopropylmethyl)4-(4-methoxyphenyl)-6-oxopiperidin-3-yl]carbamate (39 mg, 0.09 mmol) as an oil (21 mg, 73%, er = 96:4).

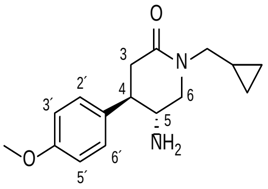

$[\alpha]^D_{20}$= +8.50 (c = 0.11, $CHCl_3$). Chiral HPLC (method A, t_R, min): 5.40. Elemental analysis calculated for $C_{16}H_{22}N_2O_2$·HCl: %C 61.83, %H 7.46, %N 9.01; experimental: %C 61.65, %H 7.26, %N 8.72. Spectroscopic data were in agreement with those described for enantiomer **1a**.

1-(Cyclopropyl)-4-(4-methoxyphenyl)piperidin-3-amine, 2. To a solution of enantiomer **1a** or **1b** (1.00 eq) in anhydrous THF (2 mL/mmol), $LiAlH_4$ (4.00 eq) was added at 0 ºC, and the reaction was warmed up to rt and stirred overnight. Then, a sat. NaOH solution and a sat. Rochelle salt solution were successively added. The mixture was stirred for 20 min, and extracted with EtOAc (x2). The combined organic layers were washed with brine, dried over Na_2SO_4, filtered and the solvent was evaporated. The residue was purified by flash chromatography (EtOAc to EtOAc/methanol 8:2) to afford (3*S*,4*S*)- or (3*R*,4*R*)-**2**.

(3*S*,4*S*)-2 (2a) was obtained from **1a** (37 mg, 0.14 mmol) as an oil (27 mg, 77%, er = 99:1).

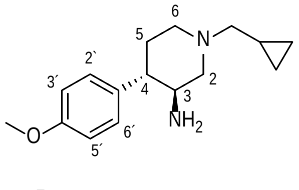

R_f: 0.28 (EtOAc/methanol 8:2). $[\alpha]^D_{20}$ = -20.8 (c = 1.00, $CHCl_3$). Chiral HPLC (method A, t_R, min): 7.25. IR (ATR): ν 2925 (NH), 1250 (COC). ^{1}H-NMR ($CDCl_3$): δ 0.13-0.18 (m, 2H, CH_{2cpr}), 0.53-0.59 (m, 2H, CH_{2cpr}), 0.89-1.02 (m, 1H, CH_{cpr}), 1.78-1.95 (m, 5H, NH_2, H_2, $2H_5$), 2.07-2.22 (m, 2H, H_4, H_6), 2.32-2.44 (m, 2H, NCH_2), 3.10 (td, *J* = 10.2, 4.0, 1H, H_3), 3.19 (d, *J* = 11.6, 1H, H_6), 3.34 (ddd, *J* = 11.0, 4.0, 1.1, 1H, H_2), 3.79 (s, 3H,

CH_3), 6.87 (d, *J* = 8.7, 2H, $H_{3'}$, $H_{5'}$), 7.16 (d, *J* = 8.7, 2H, $H_{2'}$, $H_{6'}$). ^{13}C-NMR ($CDCl_3$): δ 4.1 (CH_{2cpr}), 4.3 (CH_{2cpr}), 8.2 (CH_{cpr}), 33.0 (C_5), 51.3 (C_4), 53.0 (C_3), 54.1 (C_6), 55.4 (CH_3), 61.6 (C_2), 63.8 (NCH_2), 114.3 ($C_{3'}$, $C_{5'}$), 128.9 ($C_{2'}$, $C_{6'}$), 135.3 ($C_{1'}$), 158.6 ($C_{4'}$). 1D ^{1}H-NMR NOE: irradiation of the signal at δ 7.16 ppm (d, $H_{2'}$, $H_{6'}$) yielded NOE on 3.12 (td, H_3). HPLC (t_R, min): 2.90. MS (ESI, *m/z*, %): 261.2 ([M+H]$^+$, 100). Elemental analysis calculated for $C_{16}H_{24}N_2O{\cdot}2HCl{\cdot}H_2O$: %C 54.70, %H 8.03, %N 7.97; experimental: %C 54.39, %H 7.84, %N 7.67.

(3*R*,4*R*)-2 (2b) was obtained from **1b** (35 mg, 0.16 mmol) as an oil (28 mg, 85%, er = 99:1).

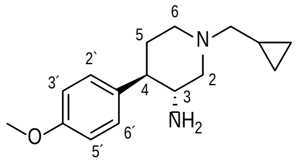

$[\alpha]^D_{20}$= +22.9 (c = 1.00, $CHCl_3$). Chiral HPLC (method A, t_R, min): 8.75. Elemental analysis calculated for $C_{16}H_{24}N_2O{\cdot}2HCl{\cdot}H_2O$: %C 54.70, %H 8.03, %N 7.97; experimental: %C 54.38, %H 7.64, %N 7.77. Spectroscopic data were in agreement with those described for enantiomer **2a**.

***tert*-Butyl 3-amino-4-(4-methoxyphenyl)piperidine-1-carboxylate, 23.** To a solution of enantiomer **15a** or **15b** (1.00 eq) in anhydrous methanol (10 mL/mmol) at 0 ºC, nickel chloride hexahydrate (2.00 eq) and $NaBH_4$ (10.00 eq) were added portionwise. The resulting black suspension was stirred at this temperature for 5 min and then warmed to rt and stirred for 3 h. An extra portion of $NaBH_4$ (5.00 eq) was added and stirring continued at rt overnight. Then, the reaction was quenched with a sat. NH_4Cl solution, diluted with water and extracted with DCM (x3). The combined organic layers were washed with brine, dried over Na_2SO_4 and filtered, and the solvent was evaporated under reduced pressure. The residue was purified by flash chromatography (DCM to DCM/ethanol 95:5) to yield title compound (3*S*,4*S*)- or (3*R*,4*R*)-**23**, as a single diasteroisomer.

(3*S*,4*S*)-23 (23a) was obtained from **15a** (100 mg, 0.30 mmol) as an oil (55 mg, 60%).

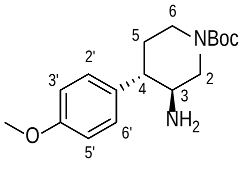

R_f: 0.44 (DCM/ethanol 9:1). $[\alpha]^D_{20}$ = +0.62 (c = 0.80, $CHCl_3$). IR (ATR): ν 3368 (NH), 1690 (C=O), 1245 (COC). ^{1}H-NMR ($CDCl_3$): δ 1.48 (s, 9H, $3CH_3$), 1.59-1.77 (m, 2H, $2H_5$), 2.29 (td, *J* = 11.2, 4.2, 1H, H_4), 2.50 (t, *J* = 11.6, 1H, H_2), 2.72-2.87 (m, 2H, H_3, H_6), 3.79 (s, 3H, OCH_3), 4.09-4.29 (m, 2H, H_2, H_6), 6.87 (d, *J* = 8.6, 2H, $H_{3'}$, $H_{5'}$), 7.14 (d, *J* = 8.6, 2H, $H_{2'}$, $H_{6'}$). ^{13}C-NMR ($CDCl_3$): δ 28.6 ($3CH_3$), 33.2 (C_5), 44.2 (br s, C_6), 51.1 (br s, C_2, C_4), 52.9 (C_3), 55.4 (OCH_3), 79.8 ($\underline{C}(CH_3)_3$), 114.3 ($C_{3'}$, $C_{5'}$), 128.8 ($C_{2'}$, $C_{6'}$), 134.7 ($C_{1'}$), 154.8 (NCOO), 158.7 ($C_{4'}$). 1D ^{1}H-NMR NOE: irradiation of the signal at δ 7.14 ppm (d, $H_{2'}$, $H_{6'}$) yielded NOE on 2.72-2.87 (m, H_3). HPLC (t_R, min): 13.1. MS (ESI, *m/z*, %): 307.2 ($[M+H]^+$, 100).

(3*R*,4*R*)-23 (23b) was obtained from **15b** (310 mg, 0.93 mmol) as an oil (130 mg, 53%).

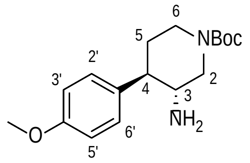

$[\alpha]^D_{20}$= -1.08 (c = 1.02, $CHCl_3$). Spectroscopic data were in agreement with those described for enantiomer **23a**.

***tert*-Butyl 3-[(cyclopropylmethyl)amino]-4-(4-methoxyphenyl)piperidine-1-carboxylate, 24.**

(3*S*,4*S*)-24 (24a). Following general procedure B using **23a** (33 mg, 0.11 mmol) and cyclopropanecarbaldehyde (12 µL, 0.16 mmol), compound **24a** was obtained as an oil (25 mg, 63%). Chromatography: hexane to hexane/EtOAc 1:1.

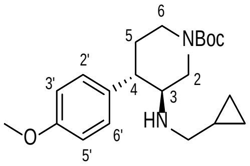

R_f: 0.25 (hexane/EtOAc 4:6). $[\alpha]^D_{20}$ = -11.5 (c = 0.90, $CHCl_3$). IR (ATR): ν 1691 (C=O), 1241 (COC). ^{1}H-NMR ($CDCl_3$): δ -0.17-(-0.01) (m, 2H, CH_{2cpr}), 0.28-0.38 (m, 2H, CH_{2cpr}), 0.66-0.78 (m, 1H, CH_{cpr}), 1.48 (s, 9H, $3CH_3$), 1.63-1.76 (m, 2H, $2H_5$), 2.10-2.16 (m, 1H, ½$NHC\underline{H}_2$), 2.39-2.50 (m, 2H, H_4, H_2), 2.55 (dd, *J* = 12.2, 6.5, 1H, ½$NHC\underline{H}_2$), 2.65 (td, *J* = 10.4, 4.1, 1H, H_3), 2.79 (t, *J* = 11.6, 1H, H_6), 3.79 (s, 3H, OCH_3), 4.15 (br s, 1H, H_6), 4.43 (br s, 1H, H_2), 6.86 (d, *J* = 8.7, 2H, $H_{3'}$, $H_{5'}$), 7.14 (d, *J* = 8.6, 2H, $H_{2'}$, $H_{6'}$). ^{13}C-NMR ($CDCl_3$): δ 3.2 (CH_{2cpr}), 3.6 (CH_{2cpr}), 11.3 (CH_{cpr}), 28.6 ($3CH_3$), 33.8 (C_5), 44.4 (br s, C_6), 48.7 (C_4), 49.1 (br s, C_2), 52.8 ($NHCH_2$), 55.4 (OCH_3), 59.0 (C_3), 79.7 ($\underline{C}(CH_3)_3$), 114.3 ($C_{3'}$, $C_{5'}$), 128.7 ($C_{2'}$, $C_{6'}$), 134.4 ($C_{1'}$), 154.8 (NCOO), 158.7 ($C_{4'}$). 1D

^{1}H-NMR NOE: irradiation of the signal at δ 7.14 ppm (d, $H_{2'}$, $H_{6'}$) yielded NOE on 2.65 (td, H_3). HPLC (t_R, min): 14.6. MS (ESI, *m/z*, %): 361.2 ($[M+H]^+$, 100).

(3*R*,4*R*)-24 (24b). Following general procedure B using **23b** (124 mg, 0.40 mmol) and cyclopropanecarbaldehyde (50 µL, 0.61 mmol), compound **24b** was obtained as an oil (93 mg, 64%). Chromatography: hexane to hexane/EtOAc 1:1.

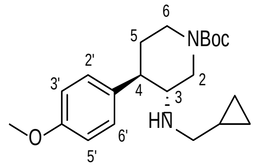

$[\alpha]^D_{20}$= +12.2 (c = 1.03, $CHCl_3$). Spectroscopic data were in agreement with those described for enantiomer **24a**.

***N*-(Cyclopropylmethyl)-4-(4-methoxyphenyl)piperidin-3-amine, 3.**

(3*S*,4*S*)-3 (3a). Following general procedure A (work-up B) using **24a** (35 mg, 0.10 mmol), compound **3a** was obtained as an oil (21 mg, 80%, er = 98:2).

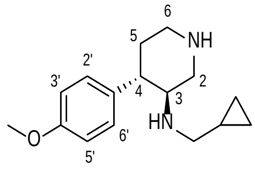

R_f: 0.20 (DCM/ethanol 9:1). $[\alpha]^D_{20}$ = -24.6 (c = 1.00, $CHCl_3$). Chiral HPLC (method G, t_R, min): 8.45. IR (ATR): ν 1248 (COC). ^{1}H-NMR ($CDCl_3$): δ -0.21-(-0.04) (m, 2H, CH_{2cpr}), 0.27-0.37 (m, 2H, CH_{2cpr}), 0.69-0.79 (m, 1H, CH_{cpr}), 1.68-1.81 (m, 2H, $2H_5$), 2.10 (dd, *J* = 12.0, 7.4, 1H, ½NHC<u>H</u>$_2$), 2.25 (br s, 2H, 2NH), 2.40-2.54 (m, 3H, H_2, H_4, ½NHC<u>H</u>$_2$), 2.68-2.81 (m, 2H, H_3, H_6), 3.15 (d, *J* = 11.8, 1H, H_6), 3.44 (dd, *J* = 11.6, 3.7, 1H, H_2), 3.80 (s, 3H, CH_3), 6.87 (d, *J* = 8.6, 2H, $H_{3'}$, $H_{5'}$), 7.18 (d, *J* = 8.6, 2H, $H_{2'}$, $H_{6'}$). ^{13}C-NMR ($CDCl_3$): δ 3.2 (CH_{2cpr}), 3.5 (CH_{2cpr}), 11.4 (CH_{cpr}), 34.7 (C_5), 46.8 (C_6), 48.8 (C_4), 52.1 (C_2), 53.0 (NCH_2), 55.4 (CH_3), 60.0 (C_3), 114.3 ($C_{3'}$, $C_{5'}$), 128.8 ($C_{2'}$, $C_{6'}$), 134.9 ($C_{1'}$), 158.6 ($C_{4'}$). 1D ^{1}H-NMR NOE: irradiation of the signal at δ 7.18 ppm (d, $H_{2'}$, $H_{6'}$) yielded NOE on 2.68-2.81 (m, H_3). HPLC (t_R, min): 3.39. MS (ESI, *m/z*, %): 261.2 ($[M+H]^+$, 100). Elemental analysis calculated for $C_{16}H_{24}N_2O\cdot 2HCl\cdot H_2O$: %C 54.70, %H 8.03, %N 7.97; experimental: %C 54.84, %H 7.71, %N 7.72.

(3*R*,4*R*)-3 (3b). Following general procedure A (work-up B) using **24b** (70 mg, 0.19 mmol), compound **3b** was obtained as an oil (36 mg, 85%, er = 98:2).

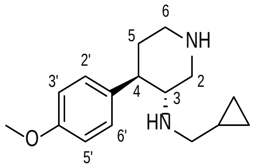

$[\alpha]^D_{20}$= +26.7 (c = 1.00, $CHCl_3$). Chiral HPLC (method G, t_R, min): 9.35. Elemental analysis calculated for $C_{16}H_{24}N_2O{\cdot}2HCl{\cdot}H_2O$: %C 54.70, %H 8.03, %N 7.97; experimental: %C 55.01, %H 7.80, %N 7.82. Spectroscopic data were in agreement with those described for enantiomer **3a**.

1-[6,9-Bis(4-methoxyphenyl)-10-nitro-6a,9,10,10a-tetrahydro-6*H*-benzo-[*c*]chromen-8-yl]-*N*-(cyclopropylmethyl)methanamine, 27.

(6*S*,6a*S*,9*R*,10*R*,10a*S*)-27 (27a). Following general procedure B using **17a** (73 mg, 0.16 mmol) and (cyclopropylmethyl)amine (28 µL, 0.32 mmol), compound **27a** was obtained as a yellow oil (83 mg, quantitative), which was used in the next step without further purification.

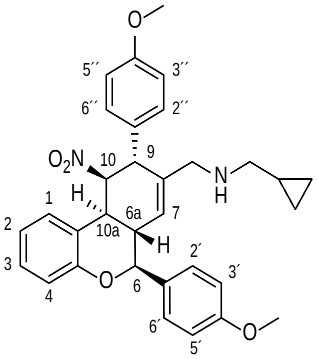

R_f: 0.61 (DCM/methanol 9:1). $[\alpha]^D_{20}$ = +25.7 (c = 0.61, $CHCl_3$). IR (ATR): ν 1610 (C=C), 1548 (NO_2), 1250 (COC). ^{1}H-NMR ($CDCl_3$): δ 0.07-0.17 (m, 3H, ½CH_{2cpr}, CH_{2cpr}), 0.26-0.35 (m, 1H, ½CH_{2cpr}), 0.80-0.93 (m, 1H, CH_{cpr}), 2.29 (dd, *J* = 12.7, 7.9, 1H, ½NHC$\underline{H}_2$CH), 2.72 (dd, *J* = 12.4, 6.8, 1H, ½NHC$\underline{H}_2$CH), 2.96 (t, *J* = 11.0, 1H, H_{6a}), 3.13 (d, *J* = 13.7, 1H, ½NHC$\underline{H}_2$), 3.35 (d, *J* = 13.7, 1H, ½NHC$\underline{H}_2$), 3.50 (d, *J* = 11.6, 1H, H_{10a}), 3.79 (s, 3H, CH_3), 3.86 (s, 3H, CH_3), 4.81 (s, 1H, H_9), 4.93 (d, *J* = 10.4, 1H, H_6), 5.36 (s, 1H, H_{10}), 5.60 (s, 1H, H_7), 6.83-6.91 (m, 2H, H_2, H_4), 6.89 (d, *J* = 8.7, 2H, $H_{3''}$, $H_{5''}$), 7.00 (d, *J* = 8.7, 2H, $H_{3'}$, $H_{5'}$), 7.11-7.19 (m, 2H, H_1, H_3), 7.28 (d, *J* = 8.7, 2H, $H_{2''}$, $H_{6''}$), 7.40 (d, *J* = 8.7, 2H, $H_{2'}$, $H_{6'}$). ^{13}C-NMR ($CDCl_3$): δ 4.1 (CH_{2cpr}), 4.4 (CH_{2cpr}), 8.1 (CH_{cpr}), 35.6 (C_{10a}), 38.6 (C_{6a}), 44.8 (C_9), 50.9 ($NHCH_2$), 51.1 (NH$\underline{C}H_2$CH), 55.51 (CH_3), 55.53 (CH_3), 82.5 (C_6), 85.8 (C_{10}), 114.5 ($C_{3'}$, $C_{5'}$), 114.8 ($C_{3''}$, $C_{5''}$), 117.2 (C_4), 119.2 (C_{10b}), 120.6 (C_2), 125.2 (C_1), 128.9 (C_3), 129.1 ($C_{2'}$, $C_{6'}$), 129.8 ($C_{1''}$), 130.2 ($C_{1'}$), 130.4 (C_7, $C_{2''}$, $C_{6''}$), 131.0 (C_8), 155.1 (C_{4a}), 159.6 ($C_{4''}$), 160.4 ($C_{4'}$). 1D ^{1}H-NMR NOE: irradiation of the

signal at δ 2.96 ppm (t, H_{6a}) yielded NOE on 4.81 (s, H_9), and 7.40 (d, $H_{2'}$, $H_{6'}$); irradiation of the signal at δ 3.50 ppm (d, H_{10a}) yielded NOE on 4.93 (d, H_6), 5.36 (s, H_{10}), and 7.28 (d, $H_{2''}$, $H_{6''}$); irradiation of the signal at δ 4.93 ppm (d, H_6) yielded NOE on 3.50 (d, H_{10a}); and irradiation at δ 5.36 ppm (s, H_{10}) yielded NOE on 3.50 (d, H_{10a}), and 7.28 (d, $H_{2''}$, $H_{6''}$). HPLC (t_R, min): 12.3. MS (ESI, *m/z*, %): 419.1 ($[M+H]^+$, 100).

(6*R*,6a*R*,9*S*,10*S*,10a*R*)-27 (27b). Following general procedure B using **17b** (87 mg, 0.18 mmol) and (cyclopropylmethyl)amine (31 µL, 0.36 mmol), compound **27b** was obtained as a yellow oil (96 mg, quantitative), which was used in the next step without further purification.

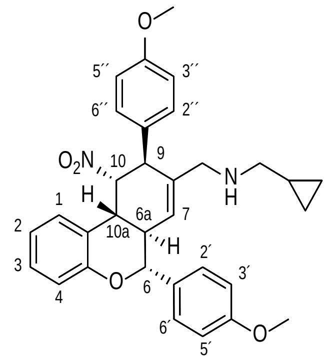

$[\alpha]^D_{20}$= -22.5 (c = 0.47, $CHCl_3$). Spectroscopic data were in agreement with those described for enantiomer **27a**.

1-Cyclopropyl-*N*-{[9-(4-methoxyphenyl)-6,6-dimethyl-10-nitro-6a,9,10,10a-tetrahydro-6*H*-benzo[*c*]chromen-8-yl]methyl}methanamine, 28

(6a*S*,9*R*,10*R*,10a*S*)-28 (28a). Following general procedure B using **18a** (105 mg, 0.27 mmol) and (cyclopropylmethyl)amine (47 µL, 0.54 mmol), compound **28a** was obtained as a yellow oil (108 mg, 90%), which was used in the next step without further purification.

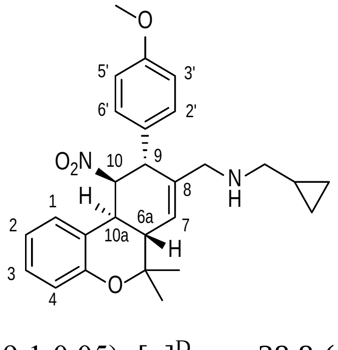

R_f: 0.64 (DCM/methanol/NH_3 9:1:0.05). $[\alpha]^D_{20}$ = -38.8 (c = 1.06, $CHCl_3$). IR (ATR): ν 1609 (C=C), 1546 (NO_2), 1252 (COC). ^{1}H-NMR ($CDCl_3$): δ 0.06-0.10 (m, 2H, CH_{2cpr}), 0.41-0.47 (m, 2H, CH_{2cpr}), 0.84-0.93 (m, 1H, CH_{cpr}), 1.25 (s, 3H, CH_3), 1.64 (s, 3H, CH_3), 2.35 (dd, *J* = 12.1, 6.8, 1H, ½NHC<u>H</u>$_2$CH), 2.44 (dd, *J* = 12.1, 6.9, 1H, ½NHC<u>H</u>$_2$CH),

2.78 (d, J = 12.2, 1H, H_{6a}), 3.16 (s, 2H, NHC$\underline{H}_2$), 3.24 (dd, J = 12.2, 2.7, 1H, H_{10a}), 3.80 (s, 3H, OCH_3), 4.32 (s, 1H, H_9), 5.38 (d, J = 2.7, 1H, H_{10}), 6.02 (s, 1H, H_7), 6.77-6.83 (m, 2H, H_2, H_4), 6.91 (d, J = 8.7, 2H, $H_{3'}$, $H_{5'}$), 7.07-7.13 (m, 2H, H_1, H_3), 7.23 (d, J = 8.7, 2H, $H_{2'}$, $H_{6'}$). ^{13}C-NMR ($CDCl_3$): δ 3.5 (CH_{2cpr}), 3.6 (CH_{2cpr}), 11.3 (CH_{cpr}), 21.7 (CH_3), 28.2 (CH_3), 31.8 (C_{10a}), 41.0 (C_{6a}), 46.0 (C_9), 53.6 ($NHCH_2$), 54.2 (NH$\underline{C}H_2$CH), 55.5 (OCH_3), 77.9 (C_6), 86.9 (C_{10}), 114.8 ($C_{3'}$, $C_{5'}$), 117.6 (C_4), 119.1 (C_{10b}), 119.8 (C_2), 123.8 (C_7), 124.8 (C_1), 128.6 (C_3), 129.9 ($C_{2'}$, $C_{6'}$), 131.5 ($C_{1'}$), 136.8 (C_8), 153.9 (C_{4a}), 159.4 ($C_{4'}$). HPLC (t_R, min): 16.7. MS (ESI, *m/z*, %): 499.0 ($[M+H]^+$, 100).

(6a*R*,9*S*,10*S*,10a*R*)-28 (28b). Following general procedure B using **18b** (120 mg, 0.31 mmol) and (cyclopropylmethyl)amine (54 µL, 0.62 mmol), compound **28b** was obtained as a yellow oil (120 mg, 88%), which was used in the next step without further purification.

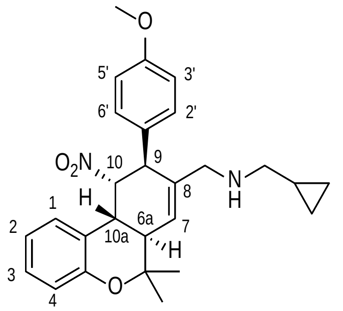

$[\alpha]^D_{20}$= +36.1 (c = 1.02, $CHCl_3$). Spectroscopic data were in agreement with those described for enantiomer **28a**.

8-{[(Cyclopropylmethyl)amino]methyl}-6,9-bis(4-methoxyphenyl)-6a,9,10,10a-tetrahydro-6*H*-benzo[*c*]chromen-10-amine, 5

(6*S*,6a*S*,9*R*,10*R*,10a*S*)-5 (5a). Following general procedure C using **27a** (83 mg, 0.16 mmol), compound **5a** was obtained as an oil (33 mg, 42%, er 99:1). Chromatography: DCM to DCM/methanol 9:1.

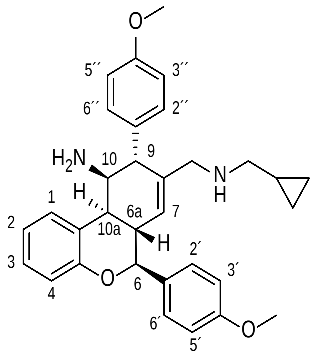

R_f: 0.32 (DCM/methanol 9:1). $[\alpha]^D_{20}$ = +25.4 (c = 0.90, $CHCl_3$). Chiral HPLC (method C, t_R, min): 29.27. IR (ATR): ν 1610 (C=C), 1245 (COC). ^{1}H-NMR ($CDCl_3$): δ -0.05-(-

0.02) (m, 2H, CH_{2cpr}), 0.34-0.42 (m, 2H, CH_{2cpr}), 0.77-0.90 (m, 1H, CH_{cpr}), 2.26-2.36 (m, 5H, NH, NH_2, NHC<u>H</u>$_2$CH), 2.80 (t, J = 10.8, 1H, H_{6a}), 3.04 (s, 2H, NHC<u>H$_2$</u>), 3.08 (d, J = 11.8, 1H, H_{10a}), 3.62 (s, 1H, H_9), 3.74 (s, 1H, H_{10}), 3.80 (s, 3H, CH_3), 3.86 (s, 3H, CH_3), 4.99 (d, J = 10.8, 1H, H_6), 5.43 (s, 1H, H_7), 6.81-6.91 (m, 2H, H_2, H_4), 6.88 (d, J = 8.7, 2H, $H_{3''}$, $H_{5''}$), 6.99 (d, J = 8.7, 2H, $H_{3'}$, $H_{5'}$), 7.04 (d, J = 7.9, 1H, H_1), 7.10 (t, J = 7.8, 1H, H_3), 7.20 (d, J = 8.7, 2H, $H_{2''}$, $H_{6''}$), 7.43 (d, J = 8.7, 2H, $H_{2'}$, $H_{6'}$). ^{13}C-NMR ($CDCl_3$): δ 3.4 (CH_{2cpr}), 3.6 (CH_{2cpr}), 10.9 (CH_{cpr}), 36.2 (C_{10a}), 37.3 (C_{6a}), 50.4 (C_9), 52.9 (C_{10}), 53.8 ($NHCH_2$), 54.4 (NH<u>C</u>H$_2$CH), 55.4 (CH_3), 55.5 (CH_3), 83.0 (C_6), 114.17 ($C_{3''}$, $C_{5''}$), 114.25 ($C_{3'}$, $C_{5'}$), 116.9 (C_4), 120.4 (C_2), 122.4 (C_7), 123.1 (C_{10b}), 124.5 (C_1), 128.0 (C_3), 129.2 ($C_{2'}$, $C_{6'}$), 129.7 ($C_{2''}$, $C_{6''}$), 131.7 ($C_{1'}$), 134.2 ($C_{1''}$), 156.1 (C_{4a}), 158.6 ($C_{4''}$), 160.0 ($C_{4'}$), C_8 not observed. 1D ^{1}H-NMR NOE: irradiation of the signal at δ 3.74 ppm (s, H_{10}) yielded NOE on 3.08 (d, H_{10a}), and 7.20 (d, $H_{2''}$, $H_{6''}$); irradiation of the signal at δ 3.50 ppm (d, H_{10a}) yielded NOE on 4.93 (d, H_6), 5.36 (s, H_{10}), and 7.28 (d, $H_{2''}$, $H_{6''}$); irradiation of the signal at δ 4.93 ppm (d, H_6) yielded NOE on 3.50 (d, H_{10a}); and irradiation of the signal at δ 5.36 ppm (s, H_{10}) yielded NOE on 3.50 (d, H_{10a}), and 7.28 (d, $H_{2''}$, $H_{6''}$). HPLC (t_R, min): 13.71. MS (ESI, *m/z*, %): 497.3 ([M+H]$^+$, 100). Elemental analysis calculated for $C_{32}H_{36}N_2O_3 \cdot 2HCl \cdot H_2O$: %C 65.41, %H 6.86, %N 4.77; experimental: %C 65.47, %H 6.55, %N 4.59.

(6*R*,6a*R*,9*S*,10*S*,10a*R*)-5 (5b). Following general procedure C using **27b** (81 mg, 0.15 mmol), compound **5b** was obtained as an oil (31 mg, 41%, er 98:2). Chromatography: DCM to DCM/methanol 9:1.

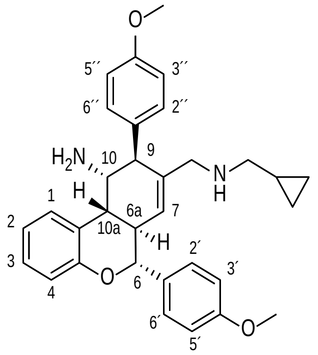

$[\alpha]^D_{20}$= -27.1 (c = 0.86, $CHCl_3$). Chiral HPLC (method C, t_R, min): 23.09. Elemental analysis calculated for $C_{32}H_{36}N_2O_3 \cdot 2HCl \cdot H_2O$: %C 65.41, %H 6.86, %N 4.77; experimental: %C 65.74, %H 6.54, %N 4.56. Spectroscopic data were in agreement with those described for enantiomer **5a**.

8-{[(Cyclopropylmethyl)amino]methyl}-9-(4-methoxyphenyl)-6,6-dimethyl-6a,9,10,10a-tetrahydro-6*H*-benzo[*c*]chromen-10-amine, 6

(6a*S*,9*R*,10*R*,10a*S*)-6 (6a). Following general procedure C using **28a** (108 mg, 0.24 mmol), compound **6a** was obtained as a yellow oil (60 mg, 60%, er 99:1). Chromatography: DCM to DCM/methanol/NH_3 95:5:0.05.

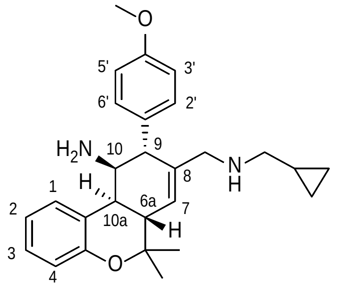

R_f: 0.32 (DCM/methanol/NH_3 9:1:0.05). $[\alpha]^D_{20}$ = -72.4 (c = 0.78, $CHCl_3$). Chiral HPLC (method D, t_R, min): 9.18. IR (ATR): ν 2925 (NH), 1609 (C=C), 1248 (COC). ^{1}H-NMR ($CDCl_3$): δ 0.05-0.10 (m, 2H, CH_{2cpr}), 0.42-0.48 (m, 2H, CH_{2cpr}), 0.83-0.95 (m, 1H, CH_{cpr}), 1.27 (s, 3H, CH_3), 1.63 (s, 3H, CH_3), 2.36 (dd, *J* = 12.0, 6.8, 1H, ½ $NHC\underline{H}_2CH$), 2.42-2.49 (m, 1H, ½$NHC\underline{H}_2CH$), 2.49-2.54(m, 1H, H_{6a}), 2.88 (d, *J* = 12.2, 1H, H_{10a}), 3.14 (AB system, *J* = 14.6, 2H, $NHC\underline{H}_2$), 3.52 (s, 1H, H_9), 3.75 (s, 1H, H_{10}), 3.78 (s, 3H, OCH_3), 5.95 (s, 1H, H_7), 6.76-6.83 (m, 2H, H_2, H_4), 6.86 (d, *J* = 8.7, 2H, $H_{3'}$, $H_{5'}$), 7.00 (d, *J* = 7.7 , 1H, H_1), 7.07 (t, *J* = 7.7, 1H, H_3), 7.19 (d, *J* = 8.6, 2H, $H_{2'}$, $H_{6'}$). ^{13}C-NMR ($CDCl_3$): δ 3.5 (CH_{2cpr}), 3.6 (CH_{2cpr}), 11.3 (CH_{cpr}), 22.4 (CH_3), 28.5 (CH_3), 31.6 (C_{10a}), 40.1 (C_{6a}), 50.6 (C_9), 53.4 (C_{10}), 54.2 ($NHCH_2$), 54.8 ($NH\underline{C}H_2CH$), 55.4 (OCH_3), 77.9 (C_6), 114.2 ($C_{3'}$, $C_{5'}$), 117.3 (C_4), 119.8 (C_2), 122.1 (C_7, C_{10b}), 124.6 (C_1), 127.8 (C_3), 129.7 ($C_{2'}$, $C_{6'}$), 134.5 ($C_{1'}$), 138.3 (C_8), 155.0 (C_{4a}), 158.6 ($C_{4'}$). 1D ^{1}H-NMR NOE: irradiation of the signal at δ 2.88 ppm (dd, H_{10a}) yielded NOE on 1.27 (s, CH_3), 3.75 (s, H_{10}), and 7.19 (d, $H_{2'}$, $H_{6'}$). HPLC (t_R, min): 12.3. MS (ESI, *m/z*, %): 419.1 ($[M+H]^+$, 100). Elemental analysis calculated for $C_{27}H_{34}N_2O_2$·2HCl: %C 65.98, %H 7.38, %N 5.70; experimental: %C 66.34, %H 7.42, %N 5.42.

(6a*R*,9*S*,10*S*,10a*R*)-6 (6b). Following general procedure B using **28b** (120 mg, 0.27 mmol), compound **6b** was obtained as a yellow oil (104 mg, 93%, er 99:1). Chromatography: DCM to DCM/methanol/NH_3 95:5:0.05.

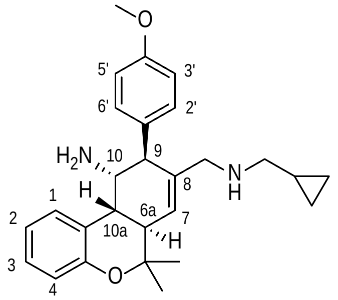

$[\alpha]^D{}_{20}$= +69.5 (c = 0.99, $CHCl_3$). Chiral HPLC (method D, t_R, min): 10.31. Elemental analysis calculated for $C_{27}H_{34}N_2O_2 \cdot 2HCl$: %C 65.98, %H 7.38, %N 5.70; experimental: %C 65.61, %H 7.23, %N 5.48. Spectroscopic data were in agreement with those described for enantiomer **6a**.

2-(Cyclopropylmethyl)-*N*-hydroxy-8-methoxy-4,4-dimethyl-2,3,3a,9b-tetrahydrochromeno [3,4-*c*]pyrrol-1(4*H*)-imine, 7

(3a*R*,9b*S*)-7 (7a). Method A: Following general procedure B using **20a** (95 mg, 0.36 mmol) and (cyclopropylmethyl)amine (62 µL, 0.72 mmol), compound **7a** was obtained as a yellow oil (20 mg, 17%). Chromatography: hexane to hexane/EtOAc 9:1.

Method B: To a solution of **20a** (160 mg, 0.57 mmol) in anhydrous DCM (5 mL/mmol), (cyclopropylmethyl)amine (75 µL, 0.85 mmol) was added and the reaction was stirred at rt for 2 h. Then, the solution was evaporated under reduced pressure, and the resulting imine was dissolved in anhydrous DCM (5 mL/mmol) and DMF (0.4 mL, 5.10 mmol). After stirring for 5 min, a 1 M solution of trichlorosilane in DCM (3.58 mL, 3.58 mmol) was added dropwise and the reaction was stirred at rt overnight. Next, a sat. $NaHCO_3$ solution was added, and the mixture was extracted with EtOAc. The organic layer was washed with water (x2), and a 1:1 mixture of water/brine (x2), dried over Na_2SO_4, filtered, and evaporated under reduced pressure. The residue was purified by column chromatography (hexane to hexane/EtOAc 9:1) to yield **7a** as an oil (144 mg, 89%, er 99:1).

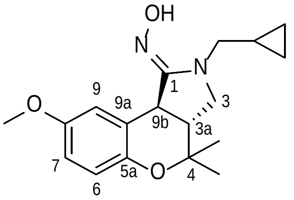

R_f: 0.42 (hexane/EtOAc 7:3). $[\alpha]^D{}_{20}$ = -27.3 (c = 1.05, $CHCl_3$). Chiral HPLC (method H, t_R, min): 6.45. IR (ATR): ν 3295 (OH), 1660 (CNOH), 1215 (COC). ^{1}H-NMR ($CDCl_3$): δ 0.21-0.32 (m, 2H, CH_{2cpr}), 0.47-0.57 (m, 2H, CH_{2cpr}), 1.08-1.18 (m, 1H, CH_{cpr}), 1.28 (CH_3), 1.43 (CH_3), 2.34 (ddd, *J* = 13.6, 11.0, 6.6, 1H, H_{3a}), 3.19 (dd, *J* = 10.9, 8.5, 1H, H_3), 3.31 (dd, *J* = 8.3, 6.7, 1H, H_3), 3.47-3.54 (m, 2H, ½NCH_2, H_{9b}), 3.68 (dd, *J* = 14.4, 7.1, 1H, ½NCH_2), 3.76 (s, 3H, OCH_3), 6.15 (br s, 1H, OH), 6.71-6.72 (m, 2H, H_6, H_7), 7.56-7.58 (m, 1H, H_9). ^{13}C-NMR ($CDCl_3$): δ 3.2 (CH_{2cpr}), 3.4 (CH_{2cpr}), 10.3 (CH_{cpr}), 21.5 (CH_3), 28.9 (CH_3), 40.4 (C_{9b}), 47.5 (C_{3a}), 51.9 (C_3), 54.6 (NCH_2), 55.9 (OCH_3), 76.4 (C_4), 112.3 (C_9), 114.8 (C_7), 117.4 (C_6), 121.5 (C_{9a}), 147.6 (C_{5a}), 153.0 (C_1), 153.2 (C_8). HPLC (t_R, min): 16.9. MS (ESI, *m/z*, %): 317.2 ($[M+H]^+$, 100).

(3a*S*,9b*R*)-7 (7b). Following method B described above using **20b** (160 mg, 0.57 mmol) and (cyclopropylmethyl)amine (98 µL, 1.14 mmol), compound **7b** was obtained as an oil (129 mg, 80%, er 99:1).

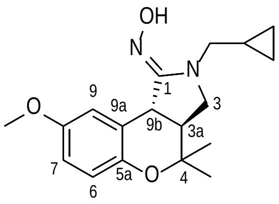

$[\alpha]^D_{20}$= +29.3 (c = 1.08, $CHCl_3$). Chiral HPLC (method H, t_R, min): 12.17. Spectroscopic data were in agreement with those described for enantiomer **20a**.

2-(Cyclopropylmethyl)-8-methoxy-4,4-dimethyl-1,2,3,3a,4,9b-hexahydro-chromeno[3,4-*c*]pyrrole, 8. To a solution of enantiomer **20a** or **20b** (1.00 eq) in anhydrous DCM (5 mL/mmol), (cyclopropylmethyl)amine (1.50 eq) was added and the reaction mixture was stirred at rt for 2 h. Then, the solvent was evaporated under reduced pressure, and the corresponding imine was dissolved in anhydrous methanol (5 mL/mmol) and cooled to 0ºC. $NaBH_4$ (2.00 eq) was then added portionwise and the reaction mixture was stirred at 0 ºC for 1 h and at rt for 1 h. Then, a sat. $NaHCO_3$ solution was added and the mixture was extracted with EtOAc (x2). The organic layers were washed with brine, dried over Na_2SO_4 and filtered, and the solvent was evaporated under reduced pressure. The residue was purified by glass column chromatography (DCM to DCM/ethanol 95:5) to yield (3a*R*,9b*S*)- or (3a*S*,9b*R*)-**8**.

(3a*R*,9b*S*)-8 (8a) was obtained from **20a** (150 mg, 0.54 mmol) as an oil (47 mg, 30%, er 96:4).

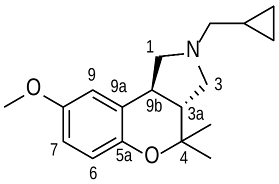

R_f: 0.36 (DCM/ethanol 9:1). $[\alpha]^D_{20}$ = +40.5 (c = 0.75, $CHCl_3$). Chiral HPLC (method E, t_R, min): 33.49. IR (ATR): ν 1488 (CN), 1216 (COC). ^{1}H-NMR ($CDCl_3$): δ 0.19-0.24 (m, 2H, CH_{2cpr}), 0.55-0.61 (m, 2H, CH_{2cpr}), 0.93-1.02 (m, 1H, CH_{cpr}), 1.27 (s, 3H, CH_3), 1.40 (s, 3H, CH_3), 2.11 (ddd, *J* = 12.7, 11.2, 8.2, 1H, H_{3a}), 2.58 (dd, *J* = 12.4, 6.8, 1H, ½NCH_2), 2.68 (dd, *J* = 12.4, 6.8, 1H, ½NCH_2), 2.75 (dd, *J* = 10.6, 8.9, 1H, H_1), 2.88 (t, *J* = 10.4, 1H, H_3), 2.98 (t, *J* = 8.9, 1H, H_3), 3.07-3.17 (m, 1H, H_{9b}), 3.64 (dd, *J* = 8.7, 6.9, 1H, H_1), 3.74 (s, 3H, OCH_3), 6.51 (d, *J* = 2.6, 1H, H_9), 6.68-6.76 (m, 2H, H_6, H_7). ^{13}C-NMR ($CDCl_3$): δ 3.9 (CH_{2cpr}), 4.2 (CH_{2cpr}), 9.8 (CH_{cpr}), 21.2 (CH_3), 29.4 (CH_3), 37.9 (C_{9b}), 49.9

(C_{3a}), 53.7 (C_3), 55.9 (OCH_3), 56.6 (C_1), 62.1 (NCH_2), 77.4 (C_4), 111.8 (C_9), 113.6 (C_7), 117.3 (C_6), 124.0 (C_{9a}), 147.8 (C_{5a}), 153.0 (C_8). 1D ^{1}H-NMR NOE: irradiation of the signal at δ 2.11 ppm (ddd, H_{3a}) yielded NOE on 1.40 (s, CH_3); and irradiation of the signal at δ 3.07-3.17 ppm (m, H_{9b}) yielded NOE on 1.27 (s, CH_3). HPLC (t_R, min): 13.4. MS (ESI, *m/z*, %): 288.2 ($[M+H]^+$, 100). Elemental analysis calculated for $C_{18}H_{25}NO_2$·HCl: %C 66.76, %H 8.09, %N 4.33; experimental: %C 67.11, %H 8.00, %N 4.08.

(3a*S*,9b*R*)-8 (8b) was obtained from **20b** (240 mg, 0.86 mmol) as an oil (81 mg, 33%, er 96:4).

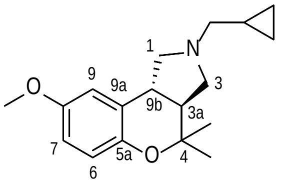

$[\alpha]^D_{20}$= -47.15 (c = 0.97, $CHCl_3$). Chiral HPLC (method E, t_R, min): 35.31. Elemental analysis calculated for $C_{18}H_{25}NO_2$·HCl: %C 66.76, %H 8.09, %N 4.33; experimental: %C 66.39, %H 7.90, %N 4.31. Spectroscopic data were in agreement with those described for enantiomer **8a**.

2-(Cyclopropylmethyl)-8-methoxy-4,4-dimethyl-2,3,3a,9b-tetrahydrochromeno[3,4-*c*]pyrrol-1(4*H*)-one, 9. To a solution of enantiomer **7a** or **7b** (1.0 eq) in methanol (21 mL/mmol) and water (50 mL/mmol), acetic acid (4.00 eq) and $NaNO_2$ (3.00 eq) were added and the mixture was stired at rt overnight. Then, the solvent was evaporated under reduced pressure and the residue was suspended in water and extracted with EtOAc (x2). The organic layers were washed with brine, dried over Na_2SO_4, filtered and evaporated under reduced pressure. The crude was purified by flash chromatography (hexane to hexane/EtOAc 85:15) to afford (3a*R*,9b*S*)- or (3a*S*,9b*R*)-**9**.

(3a*R*,9b*S*)-9 (9a) was obtained from **7a** (60 mg, 0.19 mmol) as an oil (22 mg, 40%, er 98:2).

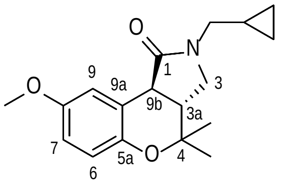

R_f: 0.27 (hexane/EtOAc 7:3). $[\alpha]^D_{20}$ = +28.6 (c = 0.74, $CHCl_3$). Chiral HPLC (method H, t_R, min): 8.24. IR (ATR): ν 1691 (C=O), 1487 (CN), 1221 (COC). ^{1}H-NMR ($CDCl_3$): δ 0.22-0.27 (m, 2H, CH_{2cpr}), 0.52-0.58 (m, 2H, CH_{2cpr}), 0.85-0.98 (m, 1H, CH_{cpr}), 1.32 (s, 3H, CH_3), 1.47 (s, 3H, CH_3), 2.39 (ddd, J = 13.8, 11.0, 6.8, 1H, H_{3a}), 3.11 (dd, J = 14.1,

7.2, 1H, ½NCH_2), 3.24-3.35 (m, 3H, ½NCH_2, H_3, H_{9b}), 3.43 (dd, J = 9.1, 6.8, 1H, H_3), 3.79 (s, 3H, OCH_3), 6.70-6.76 (m, 2H, H_6, H_7), 7.67 (dd, J = 2.9, 1.6, H_9). ^{13}C-NMR ($CDCl_3$): δ 3.66 (CH_{2cpr}), 3.68 (CH_{2cpr}), 9.4 (CH_{cpr}), 22.1 (CH_3), 28.5 (CH_3), 38.8 (C_{9b}), 46.7 (C_3), 46.9 (C_{3a}), 47.4 (NCH_2), 55.9 (OCH_3), 76.8 (C_4), 109.6 (C_9), 115.3 (C_7), 117.4 (C_6), 120.6 (C_{9a}), 147.5 (C_{5a}), 153.1 (C_8), 173.3 (C_1). HPLC (t_R, min): 20.8. MS (ESI, *m/z*, %): 302.2 ($[M+H]^+$, 100).

(3a*S*,9b*R*)-9 (9b) was obtained from **7b** (50 mg, 0.16 mmol) as an oil (12 mg, 30%, er 98:2).

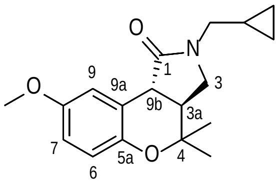

$[\alpha]^D_{20}$= -27.1 (c = 0.17, $CHCl_3$). Chiral HPLC (method H, t_R, min): 9.84. Spectroscopic data were in agreement with those described for enantiomer **9a**.

2-(4-Methoxyphenyl)-4-(nitromethyl)-3,4-dihydro-2*H*-chromene-3-carboxylic acid, 29.

(2*S*,3*S*,4*S*)-29 (29a). Following general procedure D using **19a** (40 mg, 0.12 mmol), compound **29a** was obtained as a yellow solid (42 mg, quantitative).

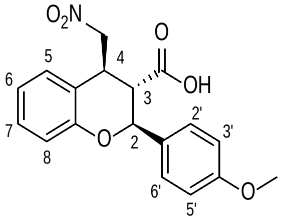

M.p.: 112-113 ºC. R_f: 0.31 (EtAOc). $[\alpha]^D_{20}$ = -11.6 (c = 0.29, methanol). IR (ATR): ν 1719 (C=O), 1554 (NO_2), 1248 (COC). ^{1}H-NMR ($CDCl_3$): δ 3.36 (t, J = 9.0, 1H, H_3), 3.82 (s, 3H, CH_3), 4.16-4.24 (m, 1H, H_4), 4.65 (d, J = 5.5, 2H, CH_2NO_2), 5.12 (d, J = 9.0, 1H, H_2), 6.92 (d, J = 8.7, 2H, $H_{3'}$, $H_{5'}$), 6.94-7.04 (m, 2H, H_6, H_8), 7.17-7.24 (m, 2H, H_5, H_7), 7.34 (d, J = 8.7, 2H, $H_{2'}$, $H_{6'}$). ^{13}C-NMR ($CDCl_3$): δ 37.1 (C_4), 49.6 (C_3), 55.5 (CH_3), 77.6 (CH_2NO_2), 78.2 (C_2), 114.3 ($C_{3'}$, $C_{5'}$), 118.1 (C_8), 119.3 (C_{4a}), 122.2 (C_6), 127.2 (C_5), 128.5 ($C_{2'}$, $C_{6'}$), 129.2 (C_7), 129.6 ($C_{1'}$), 154.9 (C_{8a}), 160.2 ($C_{4'}$), 174.9 (CO_2H). 1D ^{1}H-NMR NOE: irradiation of the signal at δ 4.16-4.24 ppm (m, H_4) yielded NOE on 5.12 (d, H_2); and irradiation of the signal at δ 4.65 ppm (d, CH_2NO_2) yielded NOE on 3.36 (t, H_3). HPLC (t_R, min): 18.44. MS (ESI, *m/z*, %): 344.1 ($[M+H]^+$, 100).

(2*R*,3*R*,4*R*)-29 (29b). Following general procedure D using **19b** (65 mg, 0.20 mmol), compound **29b** was obtained as a yellow solid (59 mg, 87%).

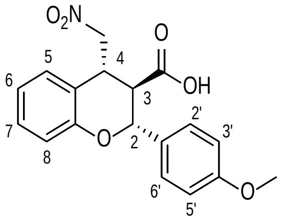

$[\alpha]^D_{20}$= +10.4 (c = 0.33, methanol). Spectroscopic data were in agreement with those described for enantiomer **29a**.

6-Methoxy-2,2-dimethyl-4-(nitromethyl)-3,4-dihydro-2*H*-chromene-3-carboxylic acid, 30.

(3*S*,4*S*)-30 (30a). Following general procedure D using **20a** (270 mg, 0.97 mmol), compound **30a** was obtained as a white solid (247 mg, 86%).

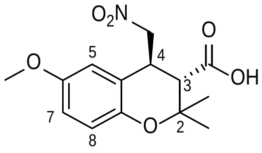

M.p.: 118-120 ºC. R_f: 0.29 (EtOAc). $[\alpha]^D_{20}$ = -8.3 (c = 0.28, $CHCl_3$). IR (ATR): ν 2925 (OH), 1709 (C=O), 1553 (NO_2), 1243 (COC). ^{1}H-NMR ($CDCl_3$): δ 1.26 (CH_3), 1.58 (CH_3), 3.10 (dd, *J* = 11.2, 1H, H_3), 3.76 (s, 3H, OCH_3), 3.92-3.99 (m, 1H, H_4), 4.70 (dd, *J* = 13.5, 4.4, 1H, ½CH_2NO_2), 4.77 (dd, *J* = 13.5, 5.6, 1H, ½CH_2NO_2), 6.71-6.74 (m, 1H, H_5) 6.77-6.81 (m, 2H, H_7, H_8). ^{13}C-NMR ($CDCl_3$): δ 20.6 (CH_3), 28.7 (CH_3), 35.0 (C_4), 52.2 (C_3), 55.9 (OCH_3), 75.2 (C_2), 78.3 (CH_2NO_2), 111.6 (C_5), 115.2 (C_7), 119.2 (C_8), 119.7 (C_{4a}), 146.8 (C_{8a}), 154.2 (C_6), 176.5 (COOH). HPLC (t_R, min): 16.8. MS (ESI, *m/z*, %): 294.1 ([M-H]$^-$, 20).

(3*R*,4*R*)-30 (30b). Following general procedure D using **20b** (364 mg, 1.29 mmol), compound **30b** was obtained as a white solid (300 mg, 79%).

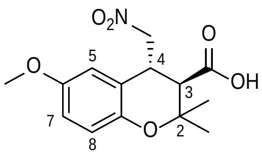

$[\alpha]^D_{20}$= +6.5 (c = 0.18, $CHCl_3$). Spectroscopic data were in agreement with those described for enantiomer **30a**.

_N_-(Cyclopropylmethyl)-2-(4-methoxyphenyl)-4-(nitromethyl)-3,4-dihydro-2_H_-chromene-3-carboxamide, 31.

(2_S_,3_S_,4_S_)-31 (31a). Following general procedure E using **29a** (45 mg, 0.13 mmol), compound **31a** was obtained as an oil (30 mg, 58%).

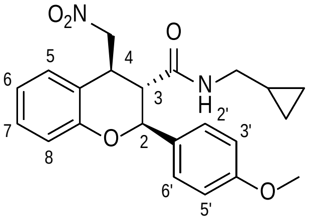

R_f: 0.53 (EtAOc). $[\alpha]^D_{20}$ = -18.0 (c = 0.48, $CHCl_3$). IR (ATR): ν 3316 (NH), 1650 (C=O), 1553 (NO_2), 1243 (COC). ^{1}H-NMR ($CDCl_3$): δ -0.16-0.02 (m, 2H, CH_{2cpr}), 0.26-0.34 (m, 2H, CH_{2cpr}), 0.47-0.60 (m, 1H, CH_{cpr}), 2.80-2.88 (m, 2H, NHC$\underline{H}_2$), 2.99 (t, *J* = 10.2, 1H, H_3), 3.81 (s, 3H, CH_3), 4.17-4.25 (m, 1H, H_4), 4.65 (dd, *J* = 13.1, 3.5, 1H, ½CH_2NO_2), 4.99 (d, *J* = 10.2, 1H, H_2), 5.00 (dd, *J* = 13.1, 4.8, 1H, ½CH_2NO_2), 5.33-5.41 (m, 1H, NH), 6.91 (d, *J* = 8.7, 2H, $H_{3'}$, $H_{5'}$), 6.93 (d, *J* = 7.9, 1H, H_8), 6.99 (td, *J* = 7.6, 1.2, 1H, H_6), 7.20 (t, *J* = 7.8, 1H, H_7), 7.30 (d, *J* = 7.8, 1H, H_5), 7.34 (d, *J* = 8.7, 2H, $H_{2'}$, $H_{6'}$). ^{13}C-NMR ($CDCl_3$): δ 3.3 (CH_{2cpr}), 3.5 (CH_{2cpr}), 10.4 (CH_{cpr}), 37.8 (C_4), 44.6 ($NHCH_2$), 51.7 (C_3), 55.5 (CH_3), 76.0 (CH_2NO_2), 79.5 (C_2), 114.3 ($C_{3'}$, $C_{5'}$), 117.9 (C_8), 119.2 (C_{4a}), 121.6 (C_6), 126.6 (C_5), 128.3 ($C_{2'}$, $C_{6'}$), 128.9 (C_7), 130.7 ($C_{1'}$), 155.2 (C_{8a}), 160.2 ($C_{4'}$), 169.8 (CONH). 1D ^{1}H-NMR NOE: irradiation of the signal at δ 2.99 ppm (t, H_3) yielded NOE on 7.34 (d, $H_{2'}$, $H_{6'}$); irradiation of the signal at δ 4.17-4.15 ppm (m, H_4) yielded NOE on 4.99 (d, H_2); and irradiation of the signal at δ 4.65 ppm (d, ½CH_2NO_2) yielded NOE on 2.99 (t, H_3). HPLC (t_R, min): 19.97. MS (ESI, *m/z*, %): 397.2 ($[M+H]^+$, 100).

(2_R_,3_R_,4_R_)-31 (31b). Following general procedure E using **29b** (60 mg, 0.17 mmol), compound **31b** was obtained as an oil (38 mg, 55%).

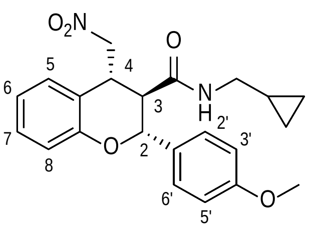

$[\alpha]^D_{20}$= +20.8 (c = 0.47, $CHCl_3$). Spectroscopic data were in agreement with those described for enantiomer **31a**.

N-(Cyclopropylmethyl)-6-methoxy-2,2-dimethyl-4-(nitromethyl)-3,4-dihydro-2*H*-chromene-3-carboxamide, 32.

(3*S*,4*S*)-32 (32a). Following general procedure E using **30a** (170 mg, 0.97 mmol), compound **32a** was obtained as an oil (179 mg, 90%).

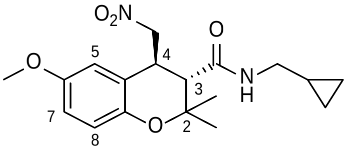

R_f: 0.32 (hexane/EtOAc 7:3). $[\alpha]^D_{20}$ = +18.9 (c = 1.00, $CHCl_3$). IR (ATR): ν 3320 (NH), 1648 (C=O), 1549 (NO_2), 1225 (COC). ^{1}H-NMR ($CDCl_3$): δ 0.21-0.26 (m, 2H, CH_{2cpr}), 0.52-0.55 (m, 2H, CH_{2cpr}), 0.97-1.03 (m, 1H, CH_{cpr}), 1.27 (CH_3), 1.48 (CH_3), 2.82 (d, *J* = 11.7, 1H, H_3), 3.10-3.27 (m, 2H, NHC$\underline{H}_2$), 3.76 (s, 3H, OCH_3), 3.85 (dt, *J* = 11.7, 3.9, 1H, H_4), 4.57 (dd, *J* = 13.4, 3.3, 1H, ½CH_2NO_2), 4.97 (dd, *J* = 13.4, 4.6, 1H, ½CH_2NO_2), 6.12 (br s, 1H, NH), 6.74-6.77 (m, 3H, H_5, H_7, H_8). ^{13}C-NMR ($CDCl_3$): δ 3.60 (CH_{2cpr}), 3.65 (CH_{2cpr}), 11.0 (CH_{cpr}), 20.1 (CH_3), 28.7 (CH_3), 35.5 (C_4), 44.8 ($NHCH_2$), 52.6 (C_3), 55.9 (OCH_3), 75.5 (C_2), 76.2 (CH_2NO_2), 111.5 (C_5), 114.9 (C_7), 118.9 (C_8), 119.4 (C_{4a}), 147.3 (C_{8a}), 153.7 (C_6), 170.6 (NHCO). HPLC (t_R, min): 19.4. MS (ESI, *m/z*, %): 349.2 ($[M+H]^+$, 100).

(3*R*,4*R*)-32 (32b). Following general procedure E using **30b** (63 mg, 1.29 mmol), compound **32b** was obtained as an oil (74 mg, quantitative).

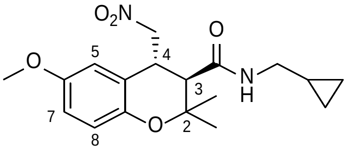

$[\alpha]^D_{20}$= -16.9 (c = 1.05, $CHCl_3$). Spectroscopic data were in agreement with those described for enantiomer **32a**.

4-(Aminomethyl)-*N*-(cyclopropylmethyl)-2-(4-methoxyphenyl)-3,4-dihydro-2*H*-chromene-3-carboxamide, 10.

(2*S*,3*S*,4*S*)-10 (10a). Following general procedure C using **31a** (80 mg, 0.20 mmol), compound **10a** was obtained as white solid (47 mg, 64%, er 99:1). Chromatography: DCM to DCM/methanol 98:2.

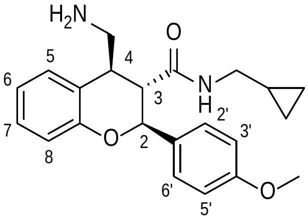

M.p.: 129-130 ºC. R_f: 0.52 (DCM/methanol 9:1). $[\alpha]^D_{20}$ = -5.6 (c = 0.44, methanol). Chiral HPLC (method H, t_R, min): 4.94. IR (ATR): ν 3309 (NH), 1641 (C=O), 1241 (COC). ^{1}H-NMR (methanol-*d4*): δ -0.10-0.00 (m, 2H, CH_{2cpr}), 0.24-0.34 (m, 2H, CH_{2cpr}), 0.52-0.66 (m, 1H, CH_{cpr}), 2.72 (dd, *J* = 13.8, 7.2, 1H, ½NHC$\underline{H_2}$), 2.83-2.97 (m, 2H, H_3, ½NH_2C$\underline{H_2}$), 2.89 (dd, *J* = 13.8, 3.5, 1H, ½NHC$\underline{H_2}$), 3.31-3.37 (m, 1H, ½NH_2C$\underline{H_2}$), 3.53 (dt, *J* = 11.4, 3.5, 1H, H_4), 3.80 (s, 3H, CH_3), 4.88 (d, *J* = 10.1, 1H, H_2), 6.87 (dd, *J* = 8.1, 1.2, 1H, H_8), 6.93 (d, *J* = 8.7, 2H, $H_{3'}$, $H_{5'}$), 7.00 (td, *J* = 7.6, 1.3, 1H, H_6), 7.16 (t, *J* = 7.3, 1H, H_7), 7.32-7.40 (m, 1H, H_5), 7.35 (d, *J* = 8.7, 2H, $H_{2'}$, $H_{6'}$). ^{13}C-NMR (methanol-*d4*): δ 3.6 (CH_{2cpr}), 3.8 (CH_{2cpr}), 11.2 (CH_{cpr}), 41.5 (C_4), 42.9 (NH_2CH_2), 44.9 ($NHCH_2$), 51.5 (C_3), 55.8 (CH_3), 80.6 (C_2), 114.8 ($C_{3'}$, $C_{5'}$), 118.4 (C_8), 122.6 (C_6), 123.2 (C_{4a}), 128.2 (C_5), 128.8 (C_7), 129.8 ($C_{2'}$, $C_{6'}$), 132.3 ($C_{1'}$), 157.5 (C_{8a}), 161.4 ($C_{4'}$), 173.2 (NHCO). 1D ^{1}H-NMR NOE: irradiation of the signal at δ 4.88 (d, H_2) yielded NOE on 3.53 (dt, H_4). HPLC (t_R, min): 13.48. MS (ESI, *m/z*, %): 367.2 ($[M+H]^+$, 100). Elemental analysis calculated for $C_{22}H_{26}N_2O_3$·HCl: %C 65.58, %H 6.75, %N 6.95; experimental: %C 65.18, %H 6.80, %N 6.64.

(2*R*,3*R*,4*R*)-10 (10b). Following general procedure C using **31b** (72 mg, 0.18 mmol), compound **10b** was obtained as a white solid (44 mg, 67%, er 99:1).

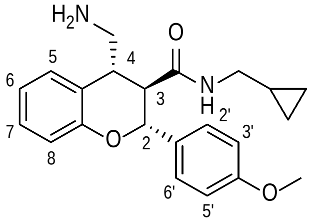

$[\alpha]^D_{20}$= +5.3 (c = 0.47, methanol). Chiral HPLC (method H, t_R, min): 6.34. Elemental analysis calculated for $C_{22}H_{26}N_2O_3$·HCl: %C 65.58, %H 6.75, %N 6.95; experimental: %C 65.20, %H 6.56, %N 6.74. Spectroscopic data were in agreement with those described for enantiomer **10a**.

4-(Aminomethyl)-*N*-(cyclopropylmethyl)-6-methoxy-2,2-dimethyl-3,4-dihydro-2*H*-chromene-3-carboxamide, 11.

(3*S*,4*S*)-11 (11a). Following general procedure C using **32a** (179 mg, 0.52 mmol), compound **11a** was obtained as an oil (144 mg, 87%, er 99:1). Chromatography: DCM to DCM/ethanol 9:1.

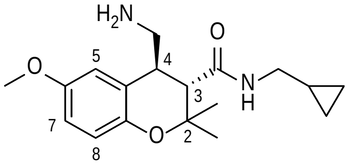

R_f: 0.36 (DCM/ethanol 9:1). $[\alpha]^D{}_{20}$ = +6.7 (c = 0.83, $CHCl_3$). Chiral HPLC (method B, t_R, min): 8.08. IR (ATR): ν 3309 (NH), 1651 (CO), 1254 (COC). ^{1}H-NMR ($CDCl_3$): δ 0.20-0.25 (m, 2H, CH_{2cpr}), 0.50-0.53 (m, 2H, CH_{2cpr}), 0.94-1.02 (m, 1H, CH_{cpr}), 1.30 (CH_3), 1.46 (CH_3), 2.79-2.86 (m, 2H, H_3, ½$NH_2C\underline{H}_2$), 3.08-3.24 (m, 2H, $NHC\underline{H}_2$), 3.31-3.41 (m, 2H, H_4, ½$NH_2C\underline{H}_2$), 3.77 (s, 3H, OCH_3), 6.44 (br s, 1H, NHCO), 6.69-6.77 (m, 2H, H_7, H_8), 6.80 (d, *J* = 2.5, 1H, H_5). ^{13}C-NMR ($CDCl_3$): δ 3.5 (CH_{2cpr}), 3.6 (CH_{2cpr}), 11.0 (CH_{cpr}), 20.5 (CH_3), 28.6 (CH_3), 36.7 (C_4), 42.2 (NH_2CH_2), 44.5 ($NHCH_2$), 51.6 (C_3), 55.9 (OCH_3), 75.4 (C_2), 111.9 (C_5), 113.5 (C_7), 118.6 (C_8), 122.7 (C_{4a}), 148.4 (C_{8a}), 153.9 (C_6), 172.1 (NHCO). HPLC (t_R, min): 12.8. MS (ESI, *m/z*, %): 319.2 ($[M+H]^+$, 100). Elemental analysis calculated for $C_{18}H_{26}N_2O_3{\cdot}HCl$: %C 60.92, %H 7.67, %N 7.89; experimental: %C 60.96, %H 7.46, %N 7.48.

(3*R*,4*R*)-11 (11b). Following general procedure C using **32b** (240 mg, 0.69 mmol), compound **11b** was obtained as an oil (142 mg, 65%, er 99:1). Chromatography: DCM to DCM/ethanol 9:1.

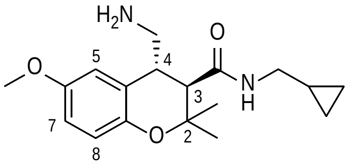

$[\alpha]^D{}_{20}$= -8.8 (c = 0.97, $CHCl_3$). Chiral HPLC (method B, t_R, min): 9.28. Elemental analysis calculated for $C_{18}H_{26}N_2O_3{\cdot}HCl$: %C 60.92, %H 7.67, %N 7.89; experimental: %C 60.66, %H 7.56, %N 7.54. Spectroscopic data were in agreement with those described for enantiomer **11a**.

1-[4-(Aminomethyl)-6-methoxy-2,2-dimethyl-3,4-dihydro-2*H*-chromen-3-yl]-*N*-(cyclopropylmethyl)methanamine, 12. To a solution of enantiomer **11a** or **11b** (1.00 eq) in anhydrous THF (5 mL/mmol), $LiAlH_4$ (5.00 eq) was added portionwise at 0 ºC and the mixture was refluxed for 24 h. The reaction was then quenched with water and 10% aqueous NaOH and stirred for 10 min. Then, the mixture was extracted with EtOAc (x2) and the combined organic layers were washed with water and brine, dried over Na_2SO_4, filtered, and concentrated under reduced pressure. The crude was purified by glass column cromatography (DCM to DCM/ethanol/NH_3 8:2:0.05) to afford compound (3*R*,4*S*)- or (3*S*,4*R*)-**12**.

(3*R*,4*S*)-12 (12a) was obtained from **11a** (55 mg, 0.17 mmol) as an oil (10 mg, 20%, er 99:1).

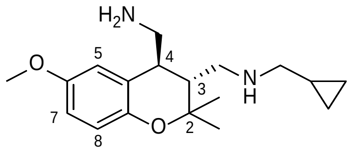

R_f: 0.27 (DCM/ethanol/NH_3) 9:1:0.05). $[\alpha]^D_{20}$ = -10.56 (c = 0.18, $CHCl_3$). Chiral HPLC (method I, t_R, min): 6.05. ^{1}H-NMR ($CDCl_3$): δ 0.10-0.15 (m, 2H, CH_{2cpr}), 0.46-0.52 (m, 2H, CH_{2cpr}), 0.89-1.01 (m, 1H, CH_{cpr}), 1.09 (CH_3), 1.49 (CH_3), 1.74 (br m, 3H, 3NH), 1.93 (ddd, *J* = 9.5, 5.8, 4.0, 1H, H_3), 2.48 (d, *J* = 6.8, 2H, $NHC\underline{H}_2CH_{cpr}$), 2.56 (dd, *J* = 12.4, 5.9, 1H, ½$NHC\underline{H}_2$), 2.66-2.72 (m, 1H, H_4), 2.81 (dd, *J* = 12.4, 3.8, 1H, ½$NHC\underline{H}_2$), 3.16 (dd, *J* = 13.4, 3.4, 1H, ½$NH_2C\underline{H}_2$), 3.23 (dd, *J* = 13.4, 4.5, 1H, ½$NH_2C\underline{H}_2$), 3.76 (s, 3H, OCH_3), 6.66-6.75 (m, 2H, H_7, H_8), 6.78 (d, *J* = 2.6, 1H, H_5). ^{13}C-NMR ($CDCl_3$): δ 3.56 (CH_{2cpr}), 3.59 (CH_{2cpr}), 11.2 (CH_{cpr}), 21.1 (CH_3), 28.7 (CH_3), 41.2 (C_4), 44.4 (C_3), 44.5 (NH_2CH_2), 51.9 ($NHCH_2$), 55.4 ($NH\underline{C}H_2CH_{cpr}$), 55.8 ($OCH_3$), 76.9 ($C_2$), 112.5 ($C_5$), 113.2 ($C_7$), 118.4 ($C_8$), 125.2 ($C_{4a}$), 148.6 ($C_{8a}$), 153.8 ($C_6$). 1D ^{1}H-NMR NOE: irradiation of the signal at δ 1.09 ppm (s, CH_3) yielded NOE on 2.66-2.72 (m, H_4), and 2.81 (dd, ½$NHC\underline{H}_2$); and irradiation of the signal at δ 1.49 ppm (m, CH_3) yielded NOE on 1.93 (ddd, H_3). HPLC (t_R, min): 4.10. MS (ESI, *m/z*, %): 305.2 ($[M+H]^+$, 100). Elemental analysis calculated for $C_{18}H_{28}N_2O_2$·2HCl: %C 57.29, %H 8.01, %N 7.42; experimental: %C 57.59, %H 7.67, %N 7.12.

(3*S*,4*R*)-12 (12b) was obtained from **11b** (72 mg, 0.23 mmol) as an oil (15 mg, 22%, er 99:1).

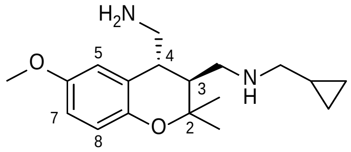

$[\alpha]^D_{20}$= +9.05 (c = 0.11, $CHCl_3$). Chiral HPLC (method I, t_R, min): 6.79. Elemental analysis calculated for $C_{18}H_{28}N_2O_2$·2HCl: %C 57.29, %H 8.01, %N 7.42; experimental: %C 57.65, %H 7.81, %N 7.48. Spectroscopic data were in agreement with those described for enantiomer **11a**.

6-(4-Methoxyphenyl)-5-methyl-5,6-dihydropyrido[2,3-*b*]pyrazine-7-carbaldehyde, 33. To a solution of enantiomer **21a** or **21b** (1.00 eq) in anhydrous DMF (5 mL/mmol), Cs_2CO_3 (2.20 eq) and iodomethane (5.00 eq) were added and the reaction was stirred for 1 h. Then, water was added and the mixture was neutralized with 1 M HCl and extracted with EtOAc (x3). The combined organic layers were washed with a 1:1 mixture of water/brine (x4), dried over Na_2SO_4 and filtered, and the solvent was evaporated under

reduced pressure to afford (*R*)- or (*S*)-**33**, which was used in the next step without further purification.

(*R*)-33 (33a) was obtained from **21a** (65 mg, 0.24) as a yellow oil (64 mg, 95%).

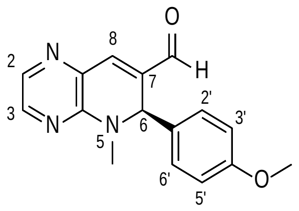

R_f: 0.40 (hexane/EtOAc 1:1). $[\alpha]^D_{20}$ = -228.7 (c = 0.60, $CHCl_3$). IR (ATR): ν 1675 (C=O), 1250 (COC). ^{1}H-NMR ($CDCl_3$): δ 2.95 (s, 3H, NCH_3), 3.76 (s, 3H, OCH_3), 5.63 (s, 1H, H_6), 6.81 (d, *J* = 8.7, 2H, $H_{3'}$, $H_{5'}$), 7.22 (d, *J* = 8.7, 2H, $H_{2'}$, $H_{6'}$), 7.30 (s, 1H, H_8), 7.81 (d, *J* = 2.6, 1H, H_2), 7.97 (d, *J* = 2.6, 1H, H_3), 9.54 (s, 1H, CHO). ^{13}C-NMR ($CDCl_3$): δ 33.5 (NCH_3), 55.4 (OCH_3), 61.8 (C_6), 114.2 ($C_{3'}$, $C_{5'}$), 128.0 ($C_{2'}$, $C_{6'}$), 132.5 ($C_{1'}$), 133.4 (C_2), 134.5 (C_{8a}), 137.8 (C_7), 140.5 (C_8), 144.9 (C_3), 153.5 (C_{4a}), 159.9 ($C_{4'}$), 190.3 (CHO). HPLC (t_R, min): 17.7. MS (ESI, *m/z*, %): 282.1 ($[M+H]^+$, 100).

(*S*)-33 (33b) was obtained from **21b** (180 mg, 0.67) as a yellow oil (188 mg, quantitative).

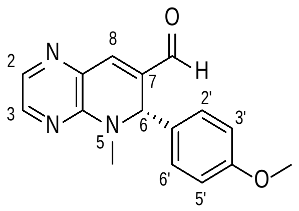

$[\alpha]^D_{20}$= +232.2 (c = 0.50, $CHCl_3$). Spectroscopic data were in agreement with those described for enantiomer **33a**.

1-Cyclopropyl-*N*-{[-6-(*p*-methoxyphenyl)-5-methyl-5,6-dihydropyrido-[2,3-*b*]pyrazin-7-yl]methyl}methanamine, 13.

(*R*)-13 (13a). Following general procedure B using **33a** (65 mg, 0.23 mmol) and (cyclopropylmethyl)amine (40 µL, 0.46 mmol), compound **13a** was obtained as a yellow oil (35 mg, 50%, er 98:2). Chromatography: DCM to DCM/ethanol 9:1.

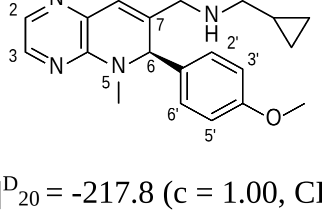

R_f: 0.40 (DCM/ethanol 9:1). $[\alpha]^D_{20}$ = -217.8 (c = 1.00, $CHCl_3$). Chiral HPLC (method E, t_R, min): 12.43. IR (ATR): ν 3405 (NH), 1248 (COC). ^{1}H-NMR ($CDCl_3$): δ 0.04-0.09 (m, 2H, CH_{2cpr}), 0.42-0.48 (m, 2H, CH_{2cpr}), 0.82-0.95 (m, 1H, CH_{cpr}), 2.36 (dd, *J* = 12.1, 6.9,

1H, ½NHC$\underline{H}_2$CH), 2.45 (dd, *J* = 12.1, 6.8, 1H, ½NHC$\underline{H}_2$CH), 2.83 (s, 3H, NCH_3), 3.12 (s, 2H, NHC$\underline{H}_2$), 3.79 (s, 3H, OCH_3), 5.31 (s, 1H, H_6), 6.54 (s, 1H, H_8), 6.84 (d, *J* = 8.7, 2H, $H_{3'}$, $H_{5'}$), 7.19 (d, *J* = 8.7, 2H, $H_{2'}$, $H_{6'}$), 7.60 (d, *J* = 3.0, 1H, H_2), 7.75 (d, *J* = 2.9 Hz, 1H, H_3). ^{13}C-NMR ($CDCl_3$): δ 3.4 (CH_{2cpr}), 3.5 (CH_{2cpr}), 11.3 (CH_{cpr}), 33.3 (NCH_3), 51.7 ($NHCH_2$), 54.4 (NH$\underline{C}H_2$CH), 55.4 (OCH_3), 66.8 (C_6), 114.3 ($C_{3'}$, $C_{5'}$), 122.2 (C_8), 128.2 ($C_{2'}$, $C_{6'}$), 131.4 (C_2), 132.6 ($C_{1'}$), 137.6 (C_{8a}), 140.6 (C_3), 143.2 (C_7), 151.4 (C_{4a}), 159.9 ($C_{4'}$). HPLC (t_R, min): 12.9. MS (ESI, *m/z*, %): 266.1 ($[M\text{-}NHCH_2CH(CH_2)_2]^+$, 100), 337.2 ($[M+H]^+$, 80). Elemental analysis calculated for $C_{20}H_{24}N_4O{\cdot}2HCl{\cdot}2H_2O$: %C 53.94, %H 6.79, %N 12.58; experimental: %C 54.07, %H 6.71, %N 12.75.

(*S*)-13 (13b). Following general procedure B using **33b** (180 mg, 0.64 mmol) and (cyclopropylmethyl)amine (1.11 mL, 1.28 mmol), compound **13b** was obtained, as a yellow oil (105 mg, 49%, er 98:2). Chromatography: DCM to DCM/ethanol 9:1.

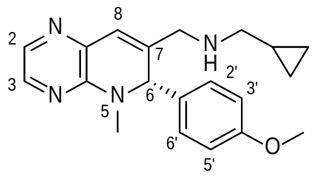

$[\alpha]^D_{20}$= +220.1 (c = 0.90, $CHCl_3$). Chiral HPLC (method E, t_R, min): 13.12. Elemental analysis calculated for $C_{20}H_{24}N_4O{\cdot}2HCl{\cdot}2H_2O$: %C 53.94, %H 6.79, %N 12.58; experimental: %C 53.59, %H 6.91, %N 12.16. Spectroscopic data were in agreement with those described for enantiomer **13a**.

***tert*-Butyl (2*R*,3*R*,4*R*)-2-(4-methoxyphenyl)-3-nitro-4-{2-[(prop-2-yn-1-yl)amino]ethyl}-1,2,3,4-tetrahydro-9*H*-carbazole-9-carboxylate, 34.** Following general procedure B using **16b** (130 mg, 0.280 mmol) and prop-2-yn-1-amine (31 mg, 0.560 mmol), compound **34** was obtained as a solid (80 mg, 57%), which was used in the next step without further purification.

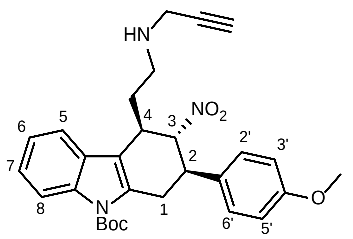

R_f: 0.37 (Hexane/EtOAc, 7:3). ^{1}H-NMR ($CDCl_3$): δ 1.64 (s, 9H, $3CH_3$), 1.98-2.09 (m, 1H, ½C$\underline{H}_2$CH$_2$NH), 2.18 (t, *J* = 2.4, 1H, C$\underline{H}$CCH$_2$), 2.41 (d, *J* = 5.5, 1H, ½C$\underline{H}_2$CH$_2$NH), 2.60-2.80 (m, 2H, CH$_2$C$\underline{H}_2$NH), 3.18-3.30 (m, 1H, H_1), 3.37 (d, *J* = 2.4, 2H, C$\underline{H}_2$CCH), 3.42-3.56 (m, 2H, H_2, H_1), 3.80 (s, 3H, OCH_3), 3.90 (td, *J* = 6.1, 2.6, 1H, H_4), 5.28 (dd, *J*

= 11.4, 9.4, 1H, H_3), 6.87 (d, *J* = 8.7, 2H, $H_{3'}$, $H_{5'}$), 7.21 (d, *J* = 8.6, 2H, $H_{2'}$, $H_{6'}$), 7.23-7.33 (m, 2H, H_6, H_7), 7.58 (d, *J* = 6.4, 1H, H_5), 8.12 (dd, *J* = 8.3, 1.2, 1H, H_8). ^{13}C-NMR: ($CDCl_3$) δ 28.4 ($3CH_3$), 29.8 ($\underline{C}H_2CH_2NH$), 32.9 (C_1), 37.8 ($\underline{C}H_2CCH$), 38.5 (C_4), 44.1 ($CH_2\underline{C}H_2NH$), 45.5 (C_2), 55.4 (OCH_3), 72.4 ($CH_2C\underline{C}H$), 79.9 ($CH_2\underline{C}CH$), 84.5 ($\underline{C}(CH_3)_3$), 93.5 (C_3), 114.4 ($C_{3'}$, $C_{5'}$), 115.0 (C_{4a}), 115.8 (C_8), 119.1 (C_5), 123.2 (C_6), 124.3 (C_7), 127.8 (C_{4b}), 128.8 ($C_{2'}$, $C_{6'}$), 131.0 (C1'), 134.5 (C_{9a}), 136.4 (C_{8a}), 150.3 (CO), 159.3 ($C_{4'}$). HPLC (t_R, min): 11.35. MS (ESI, *m/z*, %): 504.3 ($[M+H]^+$, 100).

***tert*-Butyl (2*R*,3*R*,4*R*)-3-amino-2-(4-methoxyphenyl)-4-{2-[(prop-2-yn-1-yl)amino]ethyl}-1,2,3,4-tetrahydro-9*H*-carbazole-9-carboxylate, 35**. Following general procedure C using **34** (155 mg, 0.308 mmol), compound **35** was obtained as a solid (85 mg, 58%), which was used in the next step without further purification.

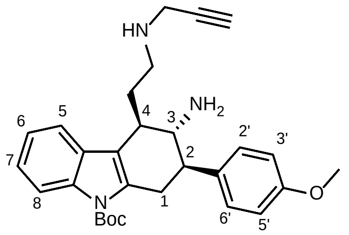

R_f: 0.43 (DCM/methanol/NH_3 10:1:0.1). ^{1}H-NMR (methanol-*d4*): δ 1.62 (s, 9H, $3CH_3$), 1.88-2.01 (m, 1H, ½$C\underline{H}_2CH_2NH$), 2.48-2.59 (m, 1H, ½$C\underline{H}_2CH_2NH$), 2.67 (t, *J* = 2.3, 1H, $CH_2CC\underline{H}$), 2.70-2.81 (m, 1H, ½$CH_2C\underline{H}_2NH$), 2.94 (dt, *J* = 11.0, 5.4, 1H, ½$CH_2C\underline{H}_2NH$), 2.99-3.14 (m, 2H, H_1, H_4), 3.17-3.26 (m, 1H, H_2), 3.32-3.36 (m, 1H, H_1), 3.37-3.48 (m, 3H, H_3, $C\underline{H}_2CCH$), 3.82 (s, 3H, OCH_3), 7.00 (d, *J* = 8.6, 2H, $H_{3'}$, $H_{5'}$), 7.22-7.30 (m, 2H, H_6, H_7), 7.34 (d, *J* = 8.7, 2H, $H_{2'}$, $H_{6'}$), 7.56 (dd, *J* = 6.2, 2.8, 1H, H_5), 8.14 (dd, *J* = 6.8, 2.6, 1H, H_8). ^{13}C-NMR (methanol-*d4*): δ 28.4 ($3CH_3$), 33.3 ($\underline{C}H_2CH_2NH$), 34.3 (C_1), 37.7 ($\underline{C}H_2CCH$), 41.3 (C_4), 43.0 (C_2), 46.8 ($CH_2\underline{C}H_2NH$), 55.8 (OCH_3), 58.0 (C_3), 74.3 ($CH_2C\underline{C}H$), 80.4 ($CH_2\underline{C}CH$), 85.3 ($\underline{C}(CH_3)_3$), 115.7 ($C_{3'}$, $C_{5'}$), 116.6 (C_8), 118.3 (C_{4a}), 119.8 (C_5), 123.8 (C_6), 124.9 (C_7), 129.3 (C_{4b}), 130.2 ($C_{2'}$, $C_{6'}$), 134.1 ($C_{1'}$), 135.9 (C_{9a}), 137.9 (C_{8a}), 151.6 (CO), 160.7 ($C_{4'}$). HPLC (t_R, min): 9.65. MS (ESI, *m/z*, %): 474.4 ($[M+H]^+$, 100).

(2*R*,3*R*,4*R*)-2-(4-Methoxyphenyl)-4-{2-[(prop-2-yn-1-yl)amino]ethyl}-2,3,4,9-tetrahydro-1*H*-carbazol-3-amine, 36. To a solution of **35** (80 mg, 0.169 mmol) in methanol (0.5 mL), HCl (37% aq solution, 1.4 mL) was added and the reaction mixture was stirred at rt for 4 h. The reaction was quenched with a sat. $NaHCO_3$ solution until pH 8 and the compound was extracted with EtOAc (2x). The combined organic phases were washed with brine, dried over Na_2SO_4, filtered and evaporated under reduce pressure to

afford compound **36** as a solid (42 mg, 67%), which was used in the next step without further purification

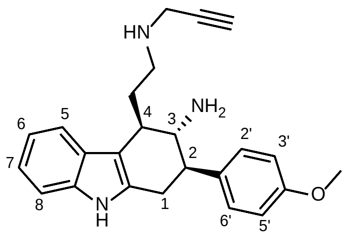

R$_f$: 0.34 (DCM/methanol/NH$_3$ 10:1:0.1). ^{1}H-NMR (methanol-*d4*): δ 1.72-1.85 (m, 1H, ½C$\underline{H}_2$CH$_2$NH), 2.76 (t, *J* = 2.5, 1H, CH$_2$CC$\underline{H}$), 2.78-3.02 (m, 3H, ½C$\underline{H}_2$CH$_2$NH, ½CH$_2$C$\underline{H}_2$NH, H$_1$), 3.07-3.24 (m, 4H, ½CH$_2$C$\underline{H}_2$NH, H$_2$, H$_4$, H$_1$), 3.46-3.52 (m, 3H, C$\underline{H}_2$CHCH, H$_3$), 3.83 (s, 3H, OCH$_3$), 6.99-7.04 (m, 3H, H$_{3'}$, H$_{5'}$, H$_6$), 7.05-7.11 (m, 1H, H$_7$), 7.30 (d, *J* = 7.5, 1H, H$_5$), 7.36 (d, *J* = 8.7, 2H, H$_{3'}$, H$_{5'}$), 7.49 (d, *J* = 7.2, 1H, H$_8$). ^{13}C-NMR (methanol-*d4*): δ 31.5 (C$_1$), 35.0 ($\underline{C}$H$_2$CH$_2$NH), 37.5 (CH$_2\underline{C}$H$_2$NH), 42.0 (C$_4$), 46.8 (C$_2$), 47.5 ($\underline{C}$H$_2$CCH), 55.8 (OCH$_3$), 59.8 (C$_3$), 74.8 (CH$_2\underline{C}$CH), 80.1 (CH$_2$C$\underline{C}$H), 110.0 (C$_{4a}$), 112.1 (C$_8$), 115.9 (C$_{3'}$, C$_{5'}$), 119.3 (C$_5$), 119.9 (C$_6$), 122.1 (C$_7$), 127.5 (C$_{4b}$), 130.2 (C$_{3'}$, C$_{5'}$), 133.4 (C$_{1'}$), 134.4 (C$_{9a}$), 138.4 (C$_{8a}$), 161.0 (C$_{4'}$). HPLC (t$_R$, min): 10.16. MS (ESI, *m/z*, %): 374.4 ([M+H]$^+$, 100).

1-{6-[(3-{4-[({2-[(2*R*,3*R*,4*R*)-3-Amino-2-(4-methoxyphenyl)-2,3,4,9-tetrahydro-1*H*-carbazol-4-yl]ethyl}amino)methyl]-1*H*-1,2,3-triazol-1-yl}propyl)amino]-6-oxohexyl}-2-{(1*E*,3*E*,5*E*)-5-[3,3-dimethyl-5-sulfo-1-(3-sulfopropyl)-1,3-dihydro-2*H*-indol-2-ylidene]penta-1,3-dien-1-yl}-3,3-dimethyl-3*H*-indol-1-ium-5-sulfonate, 37 (UCM-13369-Cy5). To a solution of compound **36** (4.9 mg, 13.1 µmol), CuSO$_4$·5H$_2$O (1.6 mg, 6.6 µmol) and sodium ascorbate (2.6 mg, 13.1 µmol) in a 1:3 mixture of DMF/H$_2$O (560 µL), sulfo-cyanine 5 azide (10.9 mg, 13.1 µmol) was added and the reaction was stirred at rt in the dark until complete consumption of starting material (36 h). Next, solvent was evaporated and the crude was purified by preparative TLC (ACN/H$_2$O, 1:1) to obtain the fluorescent probe **37** as a dark blue solid (10.8 mg, 67%)

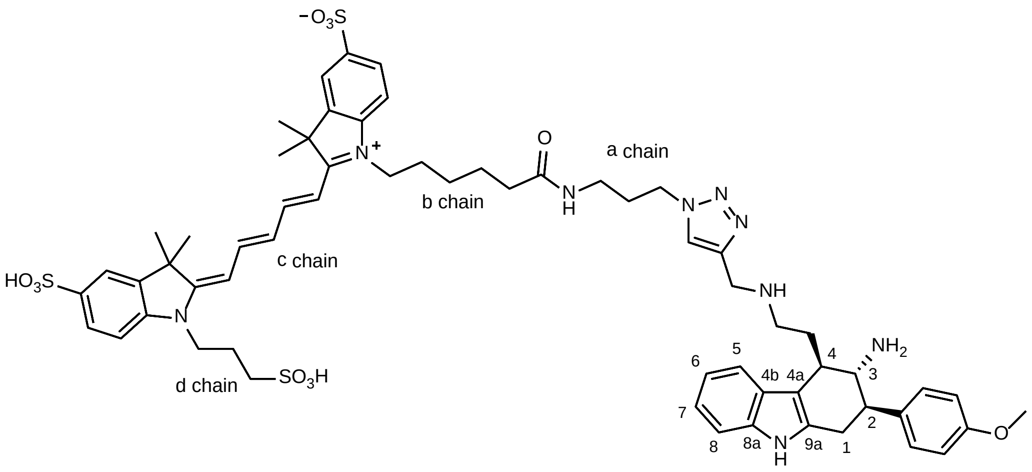

R_f: 0.33 (ACN/H_2O, 1:1). ^{1}H-NMR (700 MHz, DMSO-*d*6): δ 1.28-1.30 (m, 2H, $CH_{2\ b}$), 1.44-1.47 (m, 2H, $CH_{2\ b}$), 1.51-1.53 (m, 2H, $CH_{2\ b}$), 1.65-1.67 (m, 14H, $4CH_3$, $CH_{2\ d}$), 1.85 (t, *J* = 6.8, 2H, $CH_{2\ a}$), 1.96-2.04 (m, 6H, $CH_{2\ d}$, $CH_{2\ b}$, carbz-C$\underline{H}_2$$CH_2$NH), 2.58 (t, *J* = 6.8, 1H, H_2), 2.79 (br d, *J* = 13.5, 1H, H_1), 2.93-3.03 (m, 5H, H_1, $CH_{2\ a}$, carbz-CH_2C$\underline{H}_2$NH), 3.07 (t, *J* = 9.6, 1H, H_4), 3.75 (s, 3H, OCH_3), 3.85-3.90 (m, 2H, NHCH_2-triazol), 4.08 (br s, 2H, $CH_{2\ d}$), 4.24 (br s, 2H, $CH_{2\ b}$), 4.32 (t, *J* = 6.9, 2H, $CH_{2\ a}$), 6.29 (d, *J* = 13.8, 1H, $CH_{\ c}$), 6.46 (d, *J* = 12.7, 1H, $CH_{\ c}$), 6.49 (t, *J* = 12.5, 1H, $CH_{\ c}$), 6.92-6.95 (m, 3H, H_6, $H_{3'}$, $H_{5'}$), 7.01 (t, *J* = 7.5, 1H, H_7), 7.28 (dd, *J* = 8.2, 2.8, 2H, H_8, CH_{Ar}), 7.34 (d, *J* = 8.2, 2H, $H_{2'}$, $H_{6'}$), 7.36 (d, *J* = 8.3, 1H, CH_{Ar}), 7.48 (d, *J* = 8.0, 1H, H_5), 7.61 (t, *J* = 8.1, 2H, $2CH_{Ar}$), 7.80 (d, *J* = 1.9, 2H, $2CH_{Ar}$), 7.90 (s, 1H, OH), 8.09 (s, 1H, $CH_{triazol}$), 8.35 (t, *J* = 12.7, 2H, $2CH_{\ c}$), 10.85 (s, 1H, OH); H_3 signal is under the signal of the water from deuterated solvent. HRMS (ESI, *m/z*): calculated for $C_{61}H_{74}N_9O_{11}S_3$ $[M\text{-}H]^-$: 1204.4675, found: 1204.4636.

2.2. Cellular assays

Cell lines. Two different solid cancer, MCF-7 (breast cancer) and HCT-116 (colon cancer), one control cell fibroblasts cell line (IMR90) and two different AML cell lines, OCI-AML3 (NPM1 C+) and MOLM13 (WT NPM1) were obtained from the American Type Culture Collection (ATCC) and the German Collection of Microorganisms and Cell Cultures (DSMZ) cell repositories. Cell lines were expanded and cryogenically frozen upon acquisition to establish stocks in liquid nitrogen until use. OCI-AML3 cells also were lentivirally transduced with a third generation pRLL-luc/GFP virus and helper plasmids (pMDLg/RRE, pREV and VSV-G). Vector titration was performed using dilutions of lentiviral supernatants. Cells expressed firefly luciferase and GFP in a fusion protein driven by the CMV promoter. GFP+ cells were detected by flow cytometry and/or

quantitative real-time PCR (qPCR), or were sorted using a FACS BD INFLUX cell sorter (BD Biosciences, Franklin Lakes, NJ, USA). All cells were maintained at 37 °C in a humidified incubator in an atmosphere of 5% CO_2, and passaged every 2–3 days. Additionally, they were cultured using RPMI media (Gibco/Life Technologies; Grand Island, NY, USA) supplemented with 10% fetal bovine serum (FBS) (Hyclone) and penicillin/streptomycin.

Primary cells. $1x10^6$/mL total mononuclear bone marrow cells (as source of CD34+ HSCs) of healthy donors and AML patients were used. They were cultured using Stem Span™ supplement with pencillin/streptomycin (500 U/ml), TPO (10 µg/mL), SCF (10 µg/mL), FLT3 (10 µg/mL), IL-6 (10 µg/mL) and IL-3 (10 µg/mL). After 24 h fresh medium was added. After 72 h in maintenance cells were ready to start the assays. All cells were maintained at 37 °C in a humidified incubator in an atmosphere of 5% CO_2.

Colonospheres, mamospheres and colony formation unit assays. 30.000 cells per well of MCF-7 cells (mamospheres), HCT-116 cells (colonospheres) and CD34+ cells from patients (colony formation units) were seeded in serum free non-adherent medium or MethoCult (Stem Cell). After 10 days (mamospheres and colonospheres) and 14 days (CFU-E) we quantified the number of colonies/spheres and number of cells (CFU-E) by trypan-blue.

RNA and DNA extraction and quantitative PCR (qPCR). RNA and DNA were extracted from *in vitro* culture cells using the AllPrep DNA/RNA Mini Kit (Qiagen, Valencia, CA, USA). Gene expression was studied from the extracted RNA, processed into cDNA to quantify the expression of *NPM1* and *c-MYC*. *RPLPO* was used as a housekeeping gene. qPCR was performed on an ABI PRISM 7900HT instrument using SDS 2.2 software (Applied Biosystems; Grand Island, NY, USA) with SYBR Green (Sigma Aldrich) and the following oligos:

RPLP0: Forward: CCCTGAAGTGCTCGACATCA; Reverse: TGCGGACACCCTCCAGAA

c-MYC: Forward: CTGCGTAGTTGTGCTGATG; Reverse: GCCTCCTGGCAAAAGGTCA

NPM1: Forward: GGAGGTGGTAGCAAGGTTCC Reverse: TTCACTGGCGCTTTTTCTTCA

The relative amount of targets was determined by the comparative Ct method.

Western blot. Cells were harvested and then lysed in RIPA buffer. Lysates were cleared by centrifugation (14,000*g*, 4 °C, 30 min), protein concentration determined by Bradford

Assay (Bio-Rad Laboratories, Hercules, CA, USA) and 20 µg of total proteins was loaded onto 10% SDS-PAGE gels followed by transfer onto a nitrocellulose membrane. Blots were incubated with primary antibodies α-NPM1 (Abcam), α-MYC (Cell Signaling), α-FBXW7 (Abcam) and anti-mouse or anti-rabbit secondary antibodies (BD). An antibody to β-actin was used to check for equivalent protein loading. Membranes were developed by enhanced chemiluminescence (Clarity Western ECL substrate; Bio-Rad Laboratories). Densitometry was performed with Image Lab software, normalized to the signal of β-actin, and r-normalized with the control.

Proteomic analysis. A supplementary table (Excel file) includes all the data of the proteomic analysis, showing all the proteins identified, not only those ablated in the treated population, but also those found with different expression levels in both populations under study, and even those that were not detected in the control population but were detected in the treated population. Follows the image of a Venn diagram showing the number of proteins detected in each of the populations and the proteins that converge in both populations.

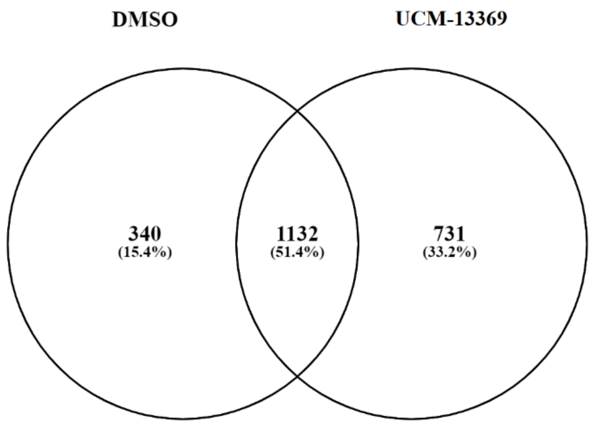

2.3. NPM1 protein studies

DNA constructs. For recombinant expression, pET28a(+) vectors encoding the native, N-end pentameric domain (residues 1-130) and the C-end domain (residues 225-294) constructs of NPM1 –all of which contained a N-terminal 6xHis tag– were available at the lab.[4] Site directed mutagenesis was performed by polymerase chain reaction (PCR) using the pET28a-NPM1 C-end WT vector to obtain the NPM1 C-end AML mutant construct (NPM1 C-end C+). The following primers were designed: 5' – TCTGTCTGGCAGTGGAGGAAGTCTCTTTAAGAAAATAGCTCGAGCACCACC ACCAC

CACC– 3’ and 5’ – TATTTTCTTAAAGAGACTTCCTCCACTGCCAGACAGAGATCTTGA ATAGCCTCTTGGTCAG– 3’. All constructs were confirmed through automated sequencing. All oligonucleotides were purchased from Sigma.

Protein expression and purification. NPM1 constructs were used to transform *E. coli* BL21(DE3) strains that were cultured overnight in lysogeny-broth (LB) agar plates supplemented with 50 μg mL^{-1} kanamycin at 37 °C. Transformed cells were grown in 250 mL precultures of LB medium supplemented with 50 μg mL^{-1} kanamycin at 37 ºC overnight to inoculate cultures of 2.5 L LB in 5 L flasks. Cells were grown to OD_{600} = 0.8 at 37 ºC, then induced with 1 mM isopropyl 1-thio-β-D-galactopyranoside (IPTG) and incubated with shaking at 30 ºC, 16 h (WT NPM1 N-end) or 18 ºC, 48 h (WT and mutant NPM1 C+ C-end). Then, cells were collected at 5,000 *g* for 10 min and resuspended in 40 mL of lysis buffer composed of 20 mM Tris-HCl (pH 8.0), 0.8 M NaCl, 10 mM imidazole, 1 mM phenylmethylsulfonyl fluoride (PMSF), protease inhibitors (cOmplete Mini, EDTA-free; Roche), 0.2 mg mL^{-1} lysozyme, 1 mM dithiothreitol (DTT) and 0.02 mg mL^{-1} DNase. Cell suspensions were sonicated for 6 min and centrifuged at 20,000 *g*, 1 h to remove cell debris. Protein purification was achieved by affinity chromatography using a Ni Sepharose 6 Fast Flow column (GE Healthcare) previously equilibrated with lysis buffer. NPM1 proteins were eluted using an imidazole gradient from 0 to 300 mM.

The protein-containing fractions were concentrated in Amicon® Ultra-15 Centrifugal Filter Units (3 or 10 kDa cutoff) until reaching the required protein concentration and dialyzed against sodium phosphate buffer (pH 7.2) with 150 mM NaCl and 1 mM DTT. The purity of the protein samples was checked by SDS–PAGE analysis (Figure S3) and protein quantification was performed using the Bradford protein assay.[5] NPM molar concentration was expressed as the pentameric form for NPM1 WT N-end and as a monomer for NPM1 C-end WT and mutant.

Mass spectrometry. The more intense band in SDS-PAGE corresponding to NPM1 C+ C-end was unstained using NH_4HCO_3 and acetonitrile and submitted to mass spectrometry analysis. Proteins were reduced by using DTT, carbaminomethylated with iodocetamide and digested using bovine tripsin in a 1:10 proportion overnight at 37 ºC. After extraction with acetonitrile and acidification, samples were desalted and concentrated with C18 tips. Mass spectra were recorded in a Hybrid Quadrupole-Orbitrap™ Mass Spectrometer (Thermo Scientific, Waltham, Massachusetts, USA)

coupled to a liquid nanochromatography Easy nLC 1200 System (Thermo Scientific). Once digested, mutant NPM1 C+ C-end sample was injected into a EASY-Spray™ C18 column, and digestion peptides were separated by using a lineal phase gradient (A: 0.1% formic acid in water; B: 20% acetonitrile, 0.1% formic acid in water) for 120 min. Spectra were acquired in DDA mode, analyzing the 10 higher intensity parent ions in each cycle. Proteome Discoverer (Thermo Scientific) was used to align the spectra with the C-end C+ protein sequence.

Nuclear magnetic resonance. One-dimensional (1D) ^{1}H NMR spectra of NPM1 C-end WT and C+ were performed on a Bruker Avance 500 MHz provided with a cryoprobe at 25 °C. Water signal was suppressed using the Excitation Sculpting solvent suppression technique. All samples were prepared in 3 mm NMR tubes containing a final volume of 0.2 mL. Protein samples were in 20 mM sodium phosphate buffer (pH 7.2) with 150 mM NaCl and 1 mM TCEP. To adjust the lock signal, 10% D_2O was added. All NMR data were processed with the TopSpin NMR 2.0 software (Bruker) and normalized using the 'nc_proc' command.

Protein-ligand molecular docking. Ab-initio cocking calculations were performed with the high ambiguity driven docking (HADDOCK2.4) web server.[6,7] Protein structure was retrieved from the NMR-resolved NPM1 C-end domain (PDB: 2LLH)[8] structure. The UCM-13369 ligand 3D structure was obtained from its SMILES string using experimental-torsion and additional basic knowledge distance geometry (ETKDG) algorithm implemented in RDKit open source cheminformatic tool.[9] Ambiguous Interaction Restraints (AIRs) for docking simulation were generated with default parameters for NPM1 C-end residues with a high relative solvent accessibility (> 50%). Random exclusion of AIRs was set to 2%. Initially, 10000 structures were generated for rigid body minimization. The 400 models with lowest AIRs energies were filtered and subjected to semi-flexible simulated annealing followed by a refinement in explicit water of the 200 top-ranked complexes. Clusters were assigned based on root-mean-squared deviations (RMSD) clustering method with 2.5 Å cutoff. Clusters were classified according to HADDOCK score and structures were analyzed with UCSF ChimeraX).[10]

2.4. Plasma protein binding (PPB) assay. The Thermo Scientific™ Single-Use RED (rapid equilibrium dialysis) Plate (8K molecular-weight cutoffs) was used for determining PPB in mouse serum (EQMS-0500, Europa Bioproducts, Ltd), following the procedure provided by the manufacturer. Samples for each replicate were prepared by spiking test

compounds (10 or 1 mM DMSO stock solutions) with mouse serum at the compound incubation concentration of 10 or 1 μM. Next, 50-400 μL of serum solution were placed into the sample chamber of the RED insert and the relative volume of the dialysis buffer (pH 7.4 PBS containing 100 mM sodium phosphate, 150 mM sodium chloride) indicated in the procedure was added to the buffer chamber. The RED block was covered with sealing tape and incubated at 37 °C on an orbital shaker at 250 rpm for 6 h. After this time, the seal was removed and 25-50 μL of each of post-dialysis samples from the buffer and the plasma chambers were pipetted into separate microcentrifuge tubes. A corresponding 25 μL or 50 μL of plasma were added to the buffer sample and an equal volume of buffer to the collected plasma sample, followed by addition of 300 μL of internal standard containing precipitation buffer (cold 90/10 acetonitrile/water with 0.1% formic acid). The samples were vortexed, incubated 30 min on ice, centrifuged for 10 min at 14,000*g*, and the supernatants were transferred to a vial. Test compound concentrations were determined in the buffer and plasma chambers from peak areas relative to the internal standard. The percentage of the test compounds bound was calculated with formula:

% Free = (Concentration buffer chamber/Concentration plasma chamber) x 100%

% Bound = 100% - % Free

Propranolol was used as a reference compound and a bound fraction of 86±2% was obtained.

3. NMR spectra

Compound **1**

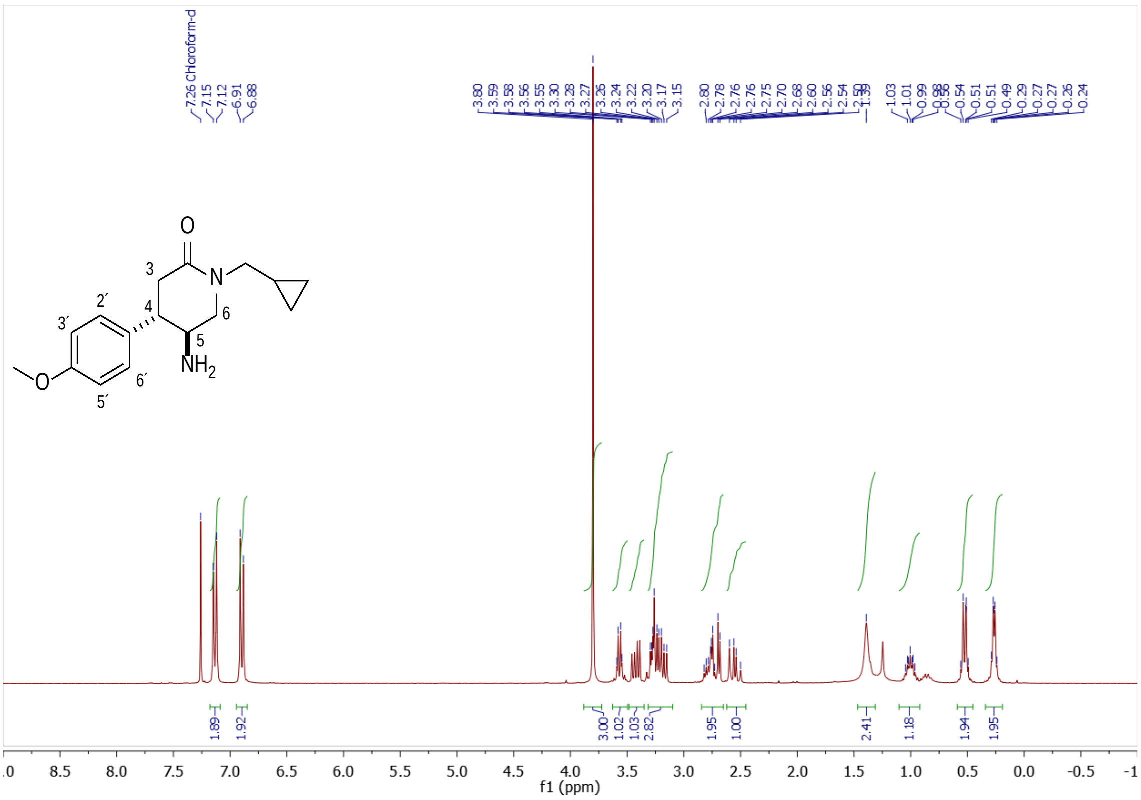

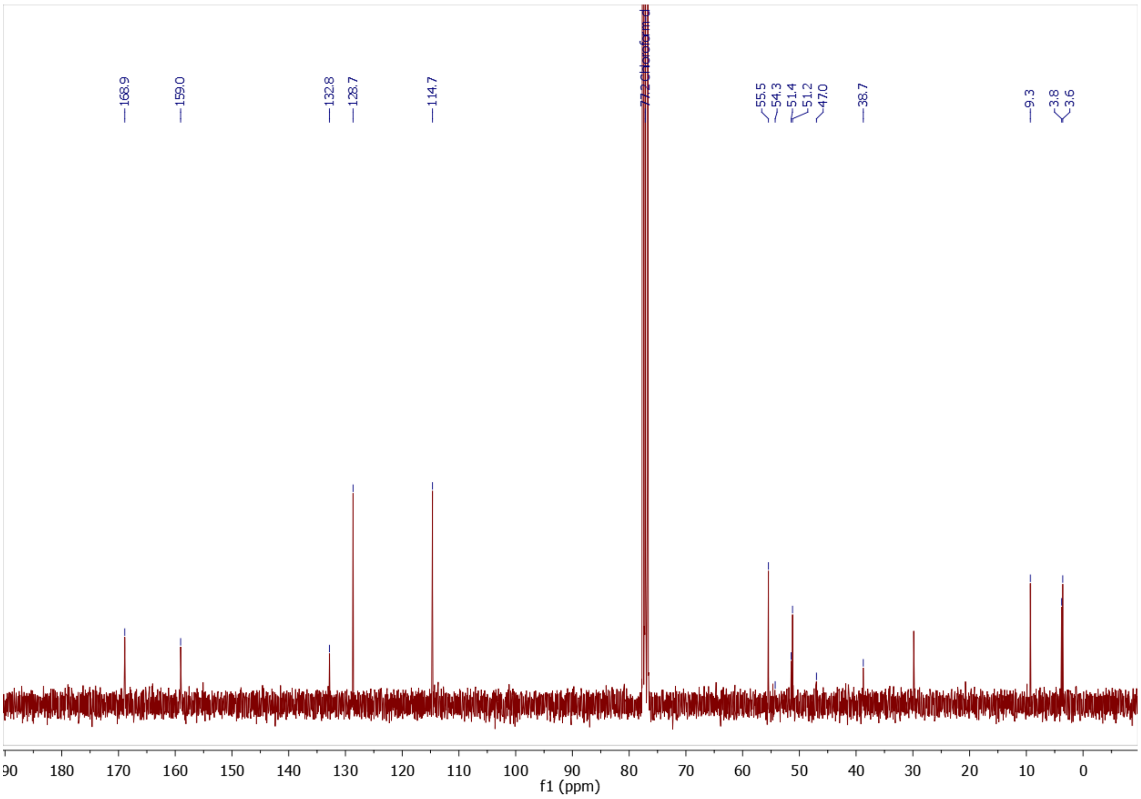

Compound **2**

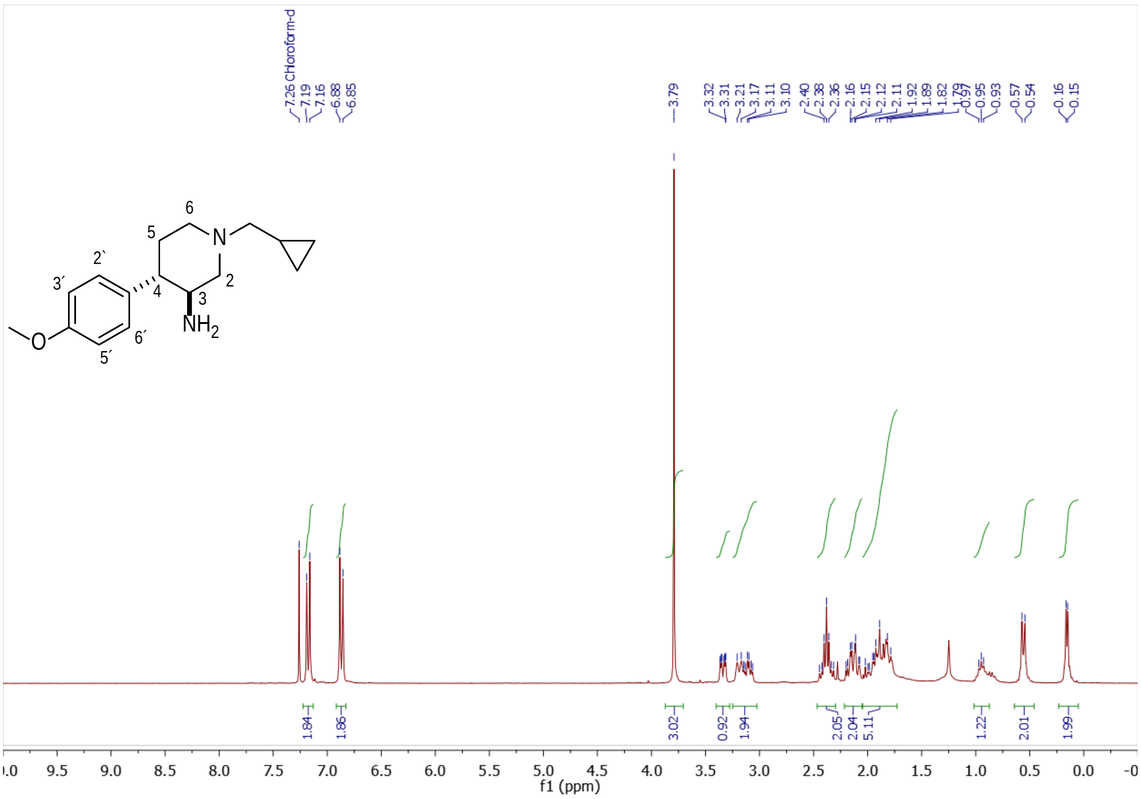
7.26 Chloroform-d
7.19
7.16
6.88
6.85
3.79
3.32
3.31
3.21
3.17
3.11
3.10
2.40
2.38
2.36
2.16
2.15
2.12
2.11
1.92
1.89
1.82
1.79
0.97
0.95
0.93
0.57
0.54
0.16
0.15
1.84
1.86
3.02
0.92
1.94
2.05
2.04
5.11
1.22
2.01
1.99
f1 (ppm)

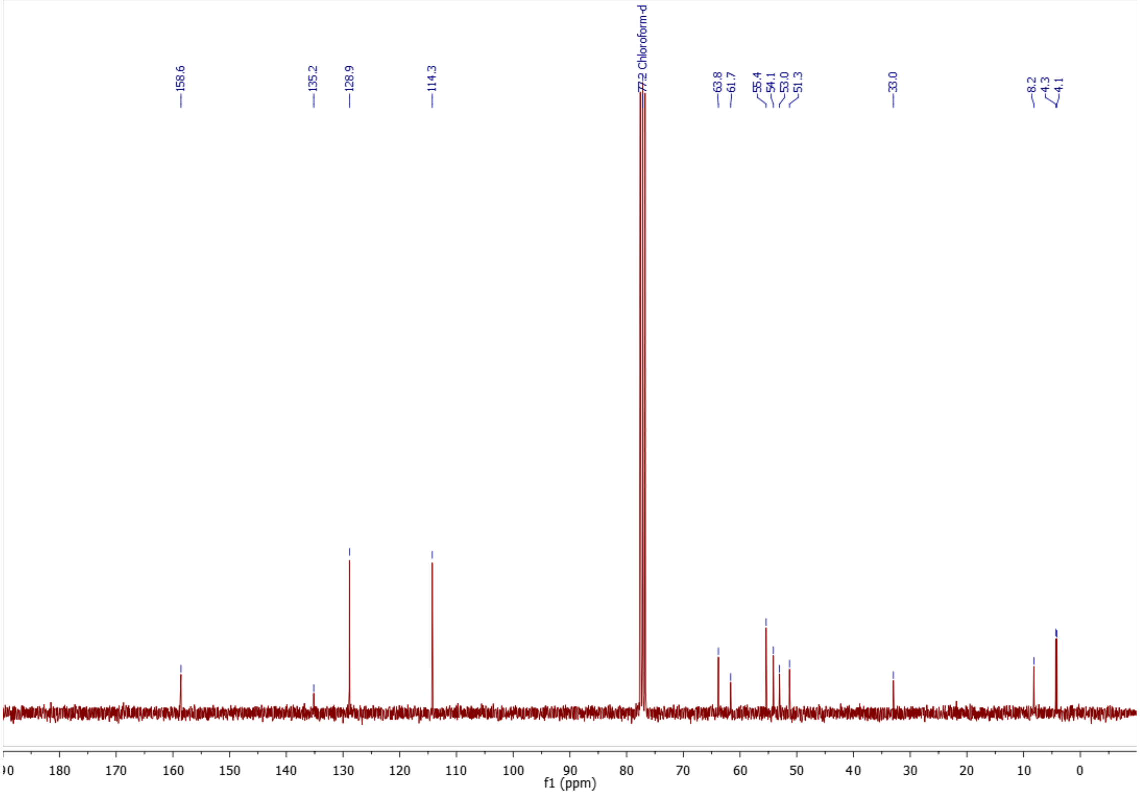
158.6
135.2
128.9
114.3
77.2 Chloroform-d
63.8
61.7
55.4
54.1
53.0
51.3
33.0
8.2
4.3
4.1
f1 (ppm)

Compound **3**

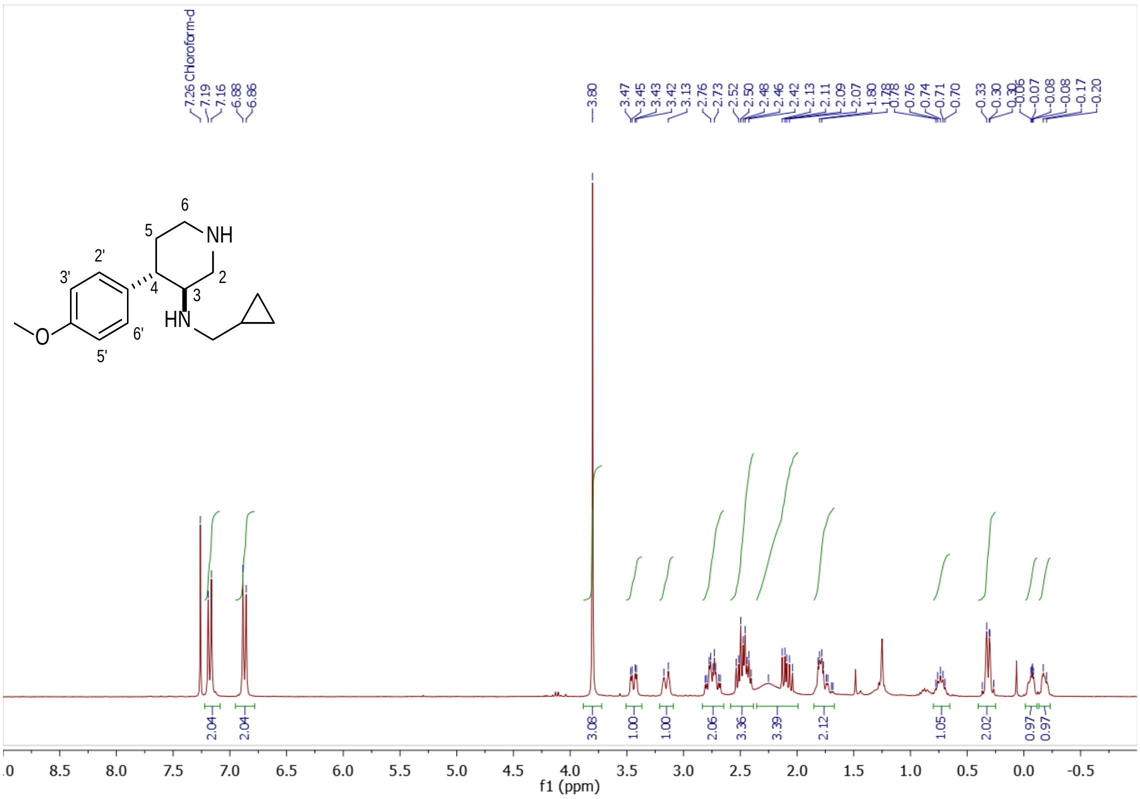
7.26 Chloroform-d
7.19
7.16
6.88
6.86
3.80
NH
HN
O
2.04
2.04
3.08
1.00
1.00
2.06
3.36
3.39
2.12
1.05
2.02
0.97
0.97
f1 (ppm)

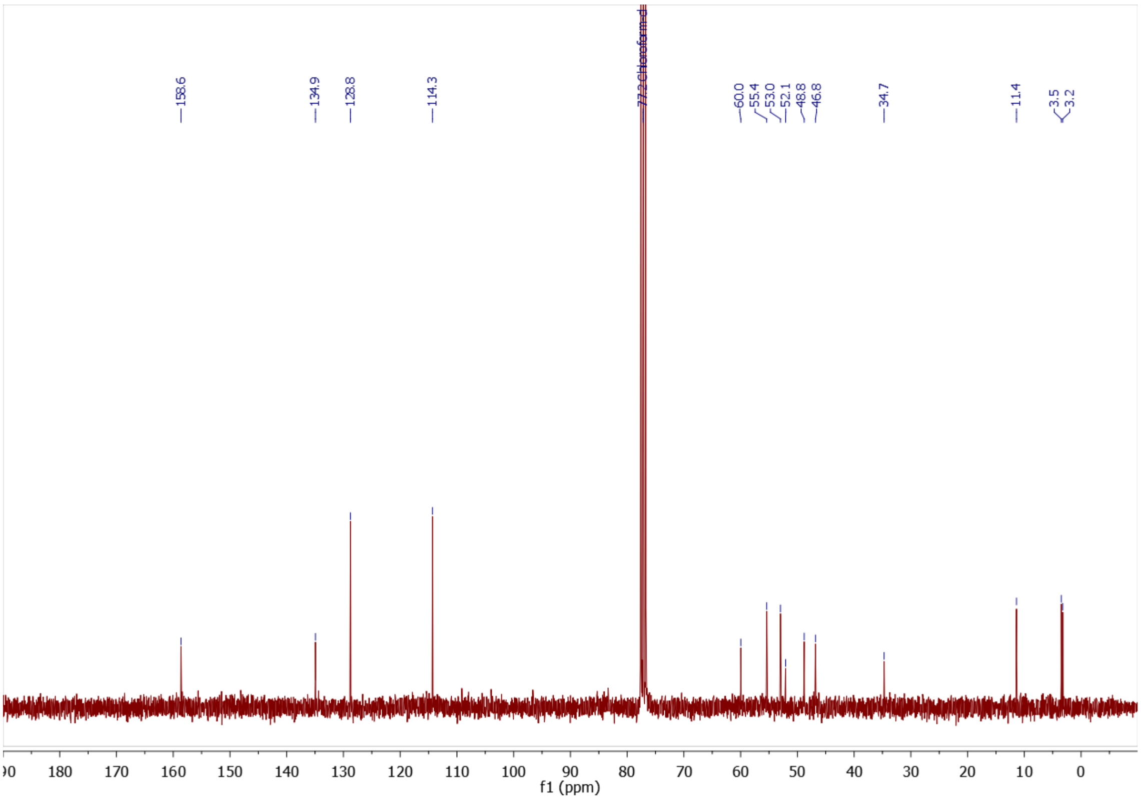
158.6
134.9
128.8
114.3
77.2 Chloroform-d
60.0
55.4
53.0
52.1
48.8
46.8
34.7
11.4
3.5
3.2
f1 (ppm)

Compound **4**

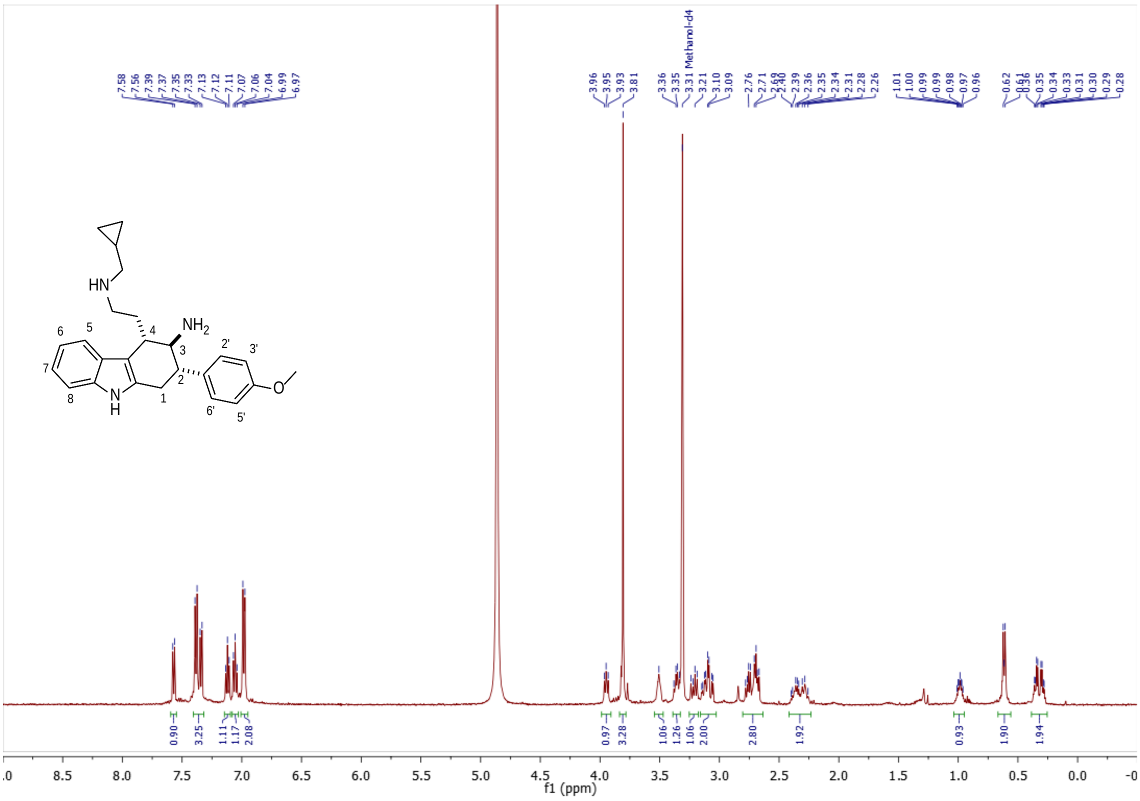
7.58 7.56 7.39 7.37 7.35 7.33 7.13 7.12 7.11 7.07 7.06 7.04 6.99 6.97
3.96 3.95 3.93 3.81 3.36 3.35 3.31 Methanol-d4 3.21 3.10 3.09
2.76 2.71 2.36 2.35 2.34 2.31 2.28 2.26
1.01 1.00 0.99 0.99 0.98 0.97 0.96
0.62 0.61 0.36 0.35 0.34 0.33 0.31 0.30 0.29 0.28
HN
NH2
N
H
O
1 2 3 4 5 6 7 8 2' 3' 5' 6'
0.90 3.25 1.11 1.17 2.08 0.97 3.28 1.06 1.26 1.06 2.00 2.80 1.92 0.93 1.90 1.94
f1 (ppm)

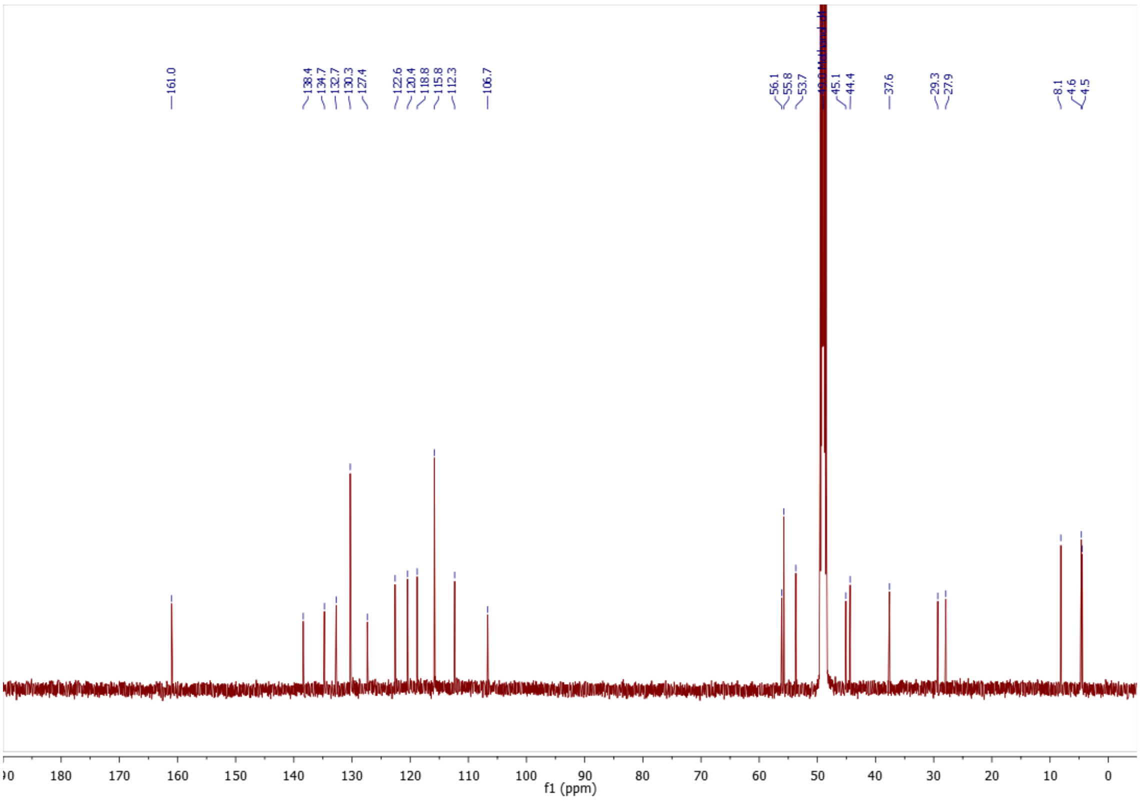
161.0
138.4 134.7 132.7 130.3 127.4
122.6 120.4 118.8 115.8 112.3
106.7
56.1 55.8 53.7 45.1 44.4 37.6 29.3 27.9 8.1 4.6 4.5
f1 (ppm)

Compound **5**

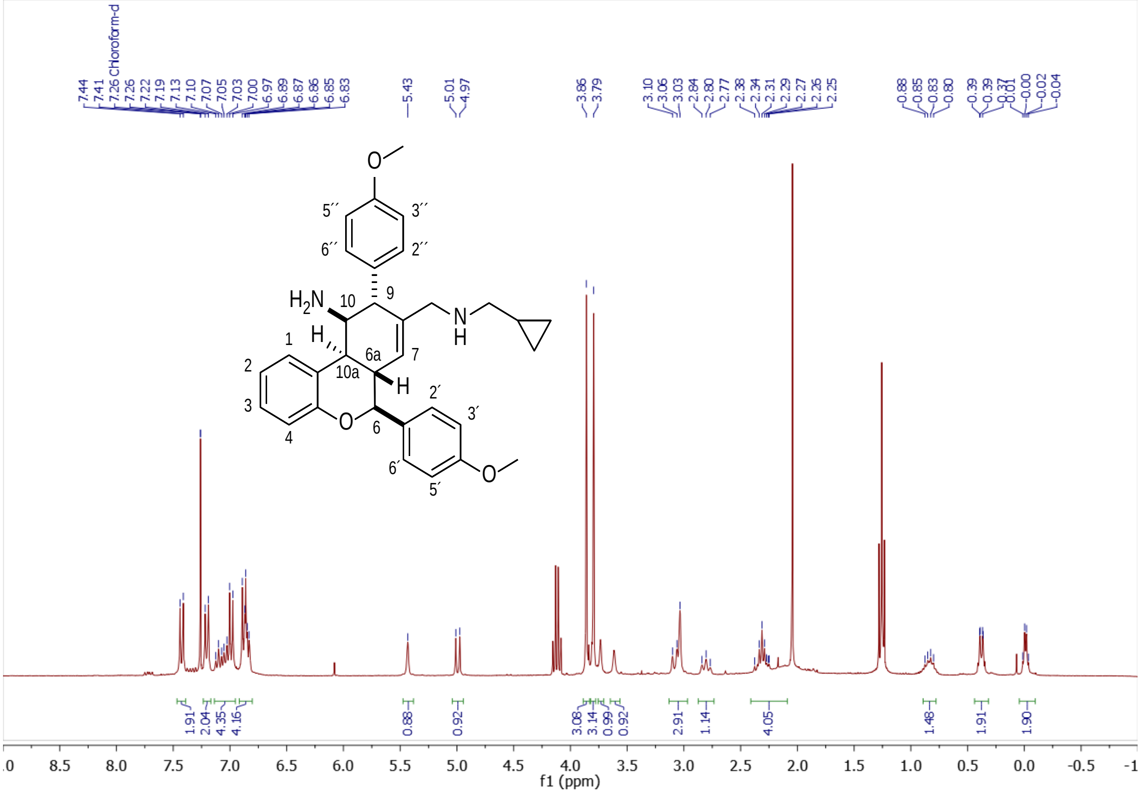

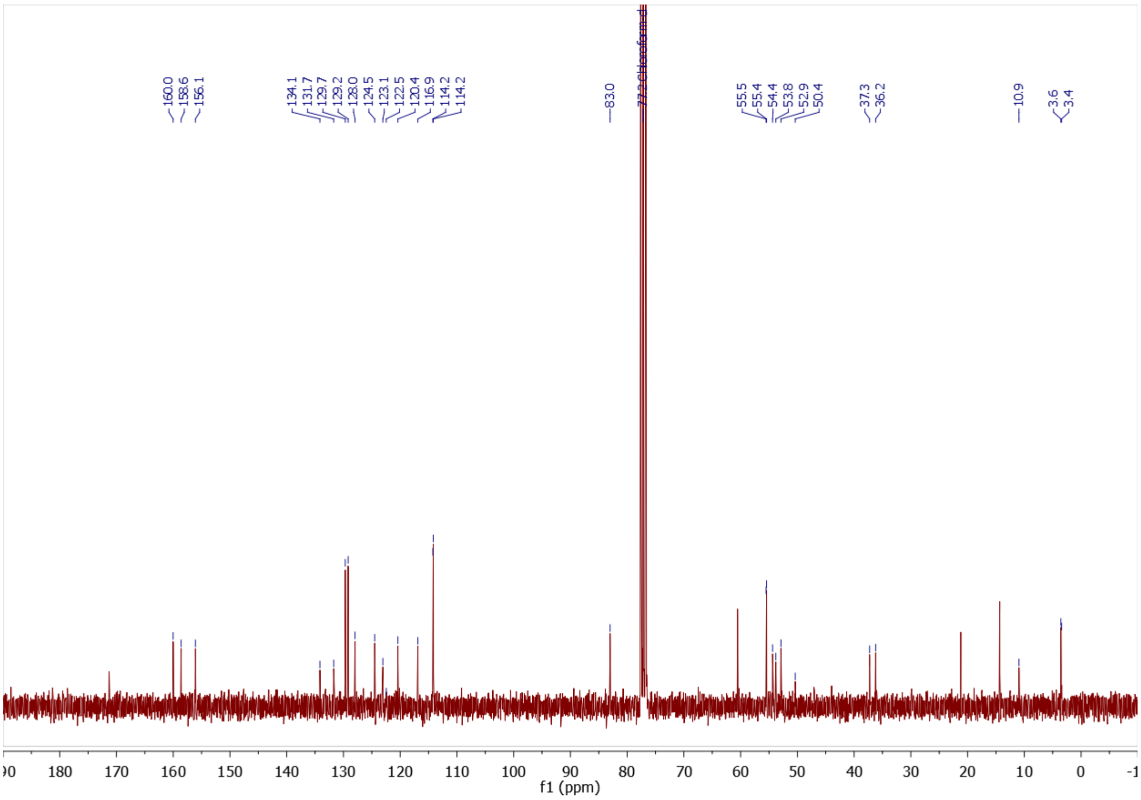

Compound **6**

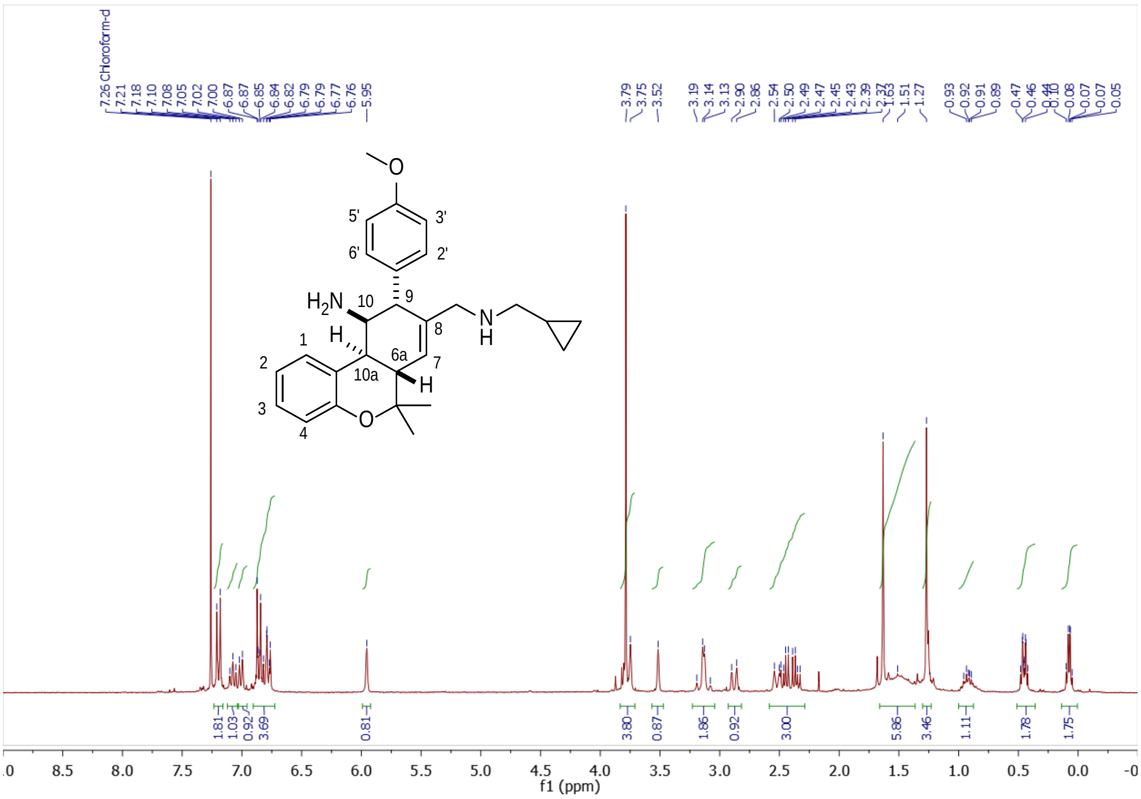
7.26 Chloroform-d
7.21
7.18
7.10
7.08
7.05
7.02
7.00
6.87
6.87
6.85
6.84
6.82
6.79
6.79
6.77
6.76
5.95
3.79
3.75
3.52
3.19
3.14
3.13
2.90
2.86
2.54
2.50
2.49
2.47
2.45
2.43
2.39
2.37
1.63
1.51
1.27
0.93
0.92
0.91
0.89
0.47
0.46
0.44
0.10
0.08
0.07
0.05
O
5'
3'
6'
2'
H2N
10
9
8
N
H
H
1
6a
7
2
10a
H
3
O
4
1.81
1.03
0.92
3.69
0.81
3.80
0.87
1.86
0.92
3.00
5.86
3.46
1.11
1.78
1.75
f1 (ppm)

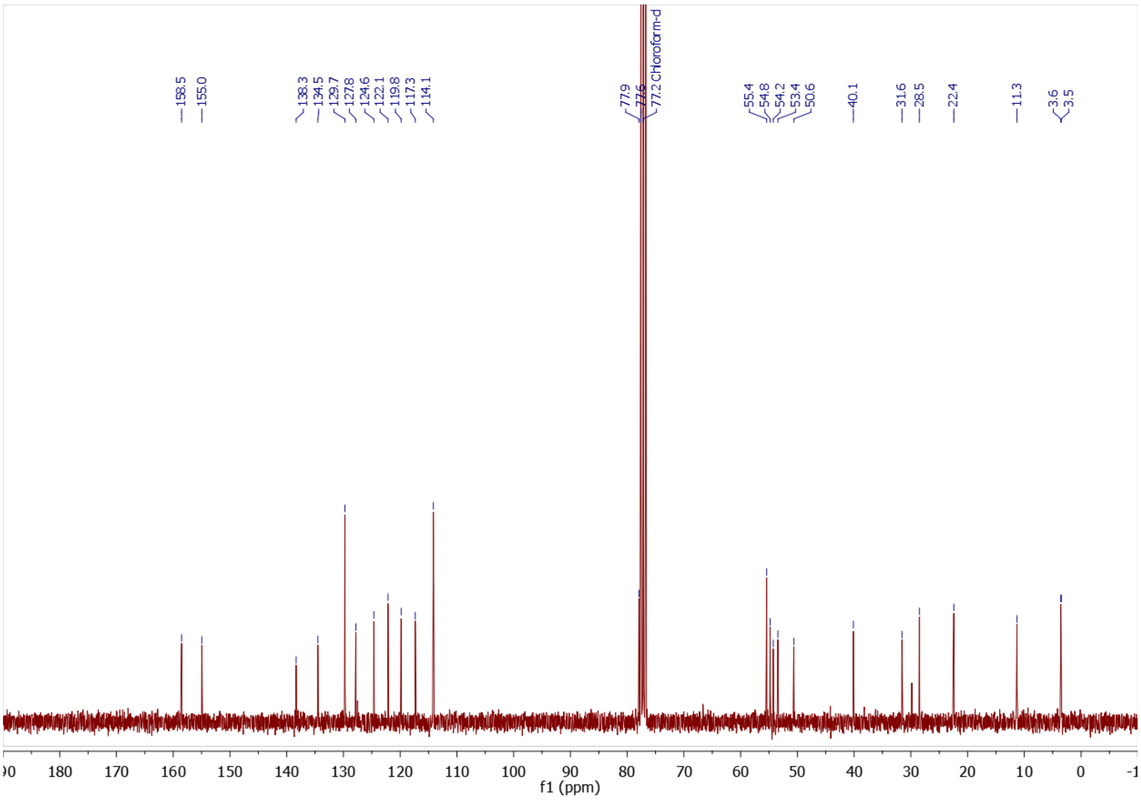
158.5
155.0
138.3
134.5
129.7
127.8
124.6
122.1
119.8
117.3
114.1
77.9
77.6
77.2 Chloroform-d
55.4
54.8
54.2
53.4
50.6
40.1
31.6
28.5
22.4
11.3
3.6
3.5
f1 (ppm)

Compound **7**

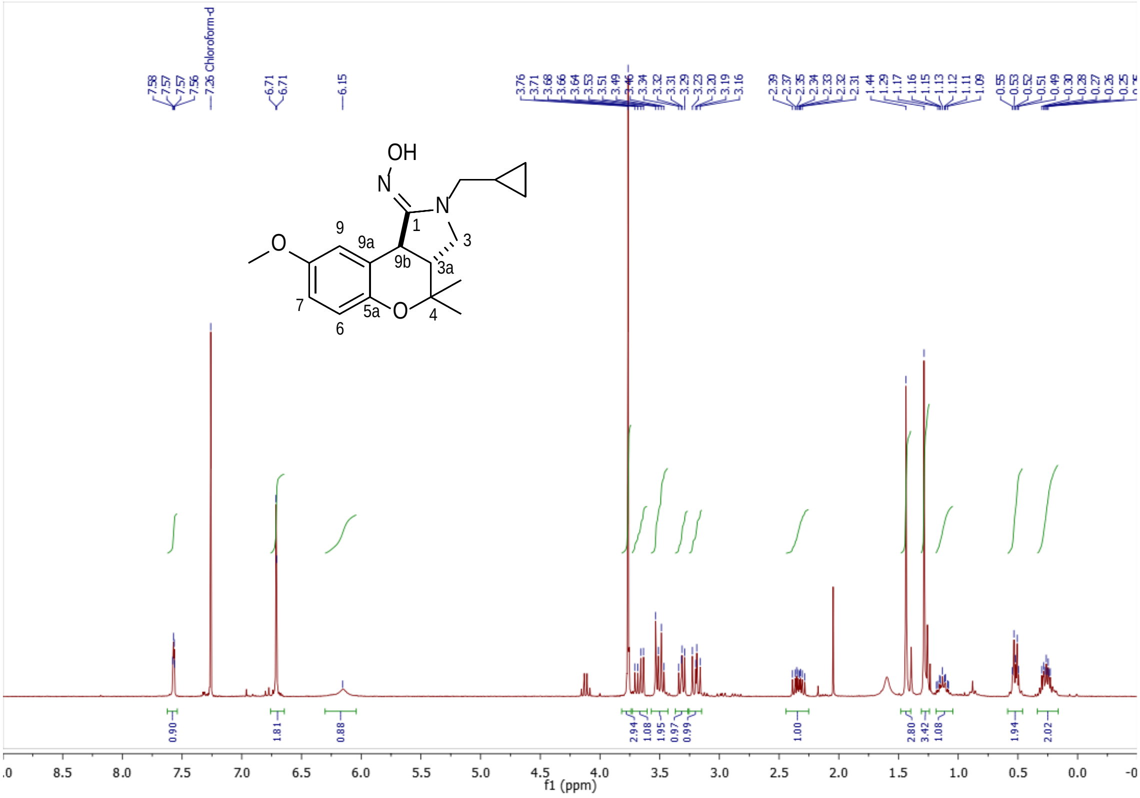
7.58
7.57
7.57
7.56
7.26 Chloroform-d
6.71
6.71
6.15
3.76
3.71
3.68
3.66
3.64
3.53
3.51
3.49
3.46
3.34
3.32
3.31
3.29
3.23
3.20
3.19
3.16
2.39
2.37
2.35
2.34
2.33
2.32
2.31
1.44
1.29
1.17
1.16
1.15
1.13
1.12
1.11
1.09
0.55
0.53
0.52
0.51
0.49
0.30
0.28
0.27
0.26
0.25
OH
N
N
O
O
9
9a
9b
1
3
3a
4
5a
6
7
0.90
1.81
0.88
2.94
1.08
1.95
0.97
0.99
1.00
2.80
3.42
1.08
1.94
2.02
f1 (ppm)

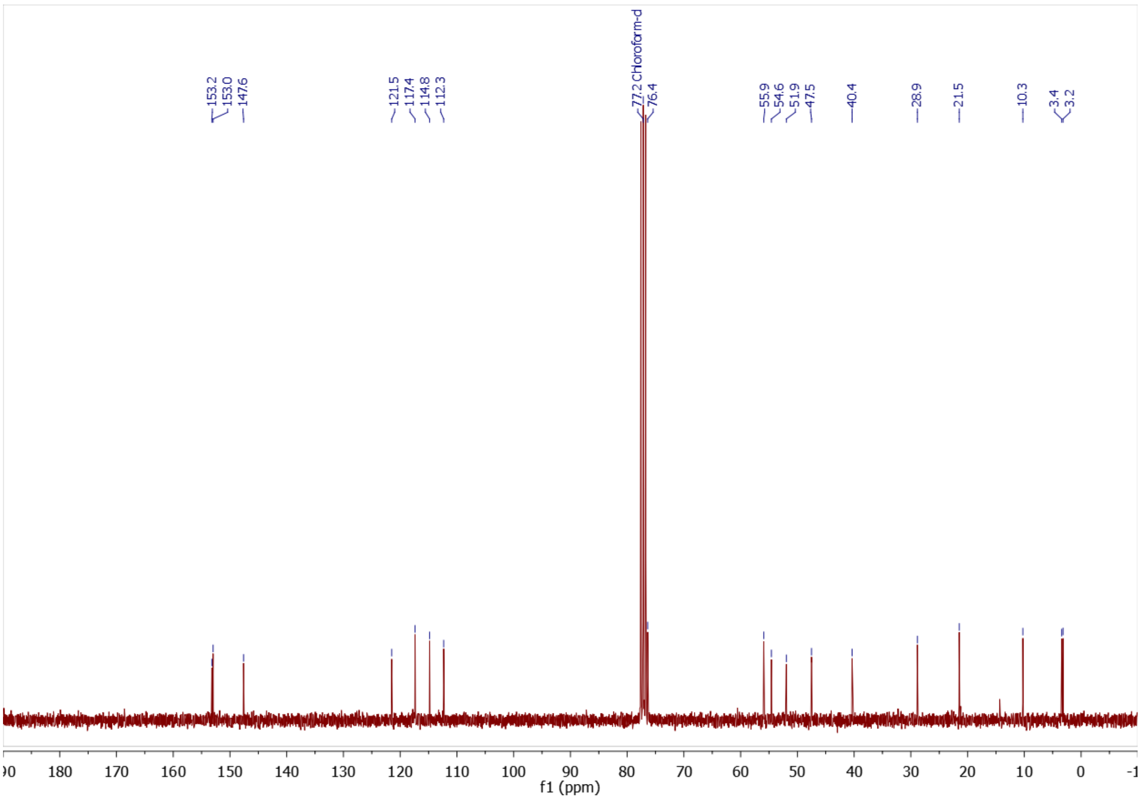
153.2
153.0
147.6
121.5
117.4
114.8
112.3
77.2 Chloroform-d
76.4
55.9
54.6
51.9
47.5
40.4
28.9
21.5
10.3
3.4
3.2
f1 (ppm)

Compound **8**

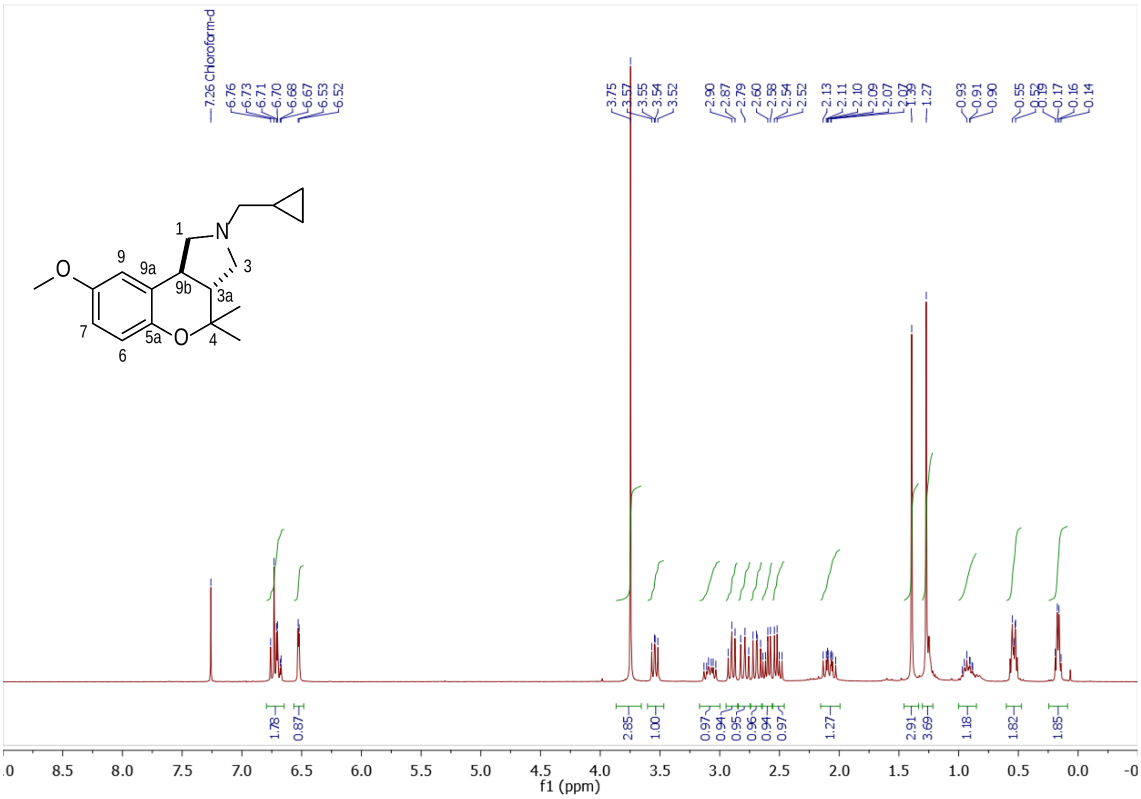
7.26 Chloroform-d
6.76
6.73
6.71
6.70
6.68
6.67
6.53
6.52
3.75
3.57
3.55
3.54
3.52
2.90
2.87
2.79
2.60
2.58
2.54
2.52
2.13
2.11
2.10
2.09
2.07
1.39
1.27
0.93
0.91
0.90
0.55
0.17
0.16
0.14
1.78
0.87
2.85
1.00
0.97
0.94
0.95
0.96
0.94
0.97
1.27
2.91
3.69
1.18
1.82
1.85
f1 (ppm)

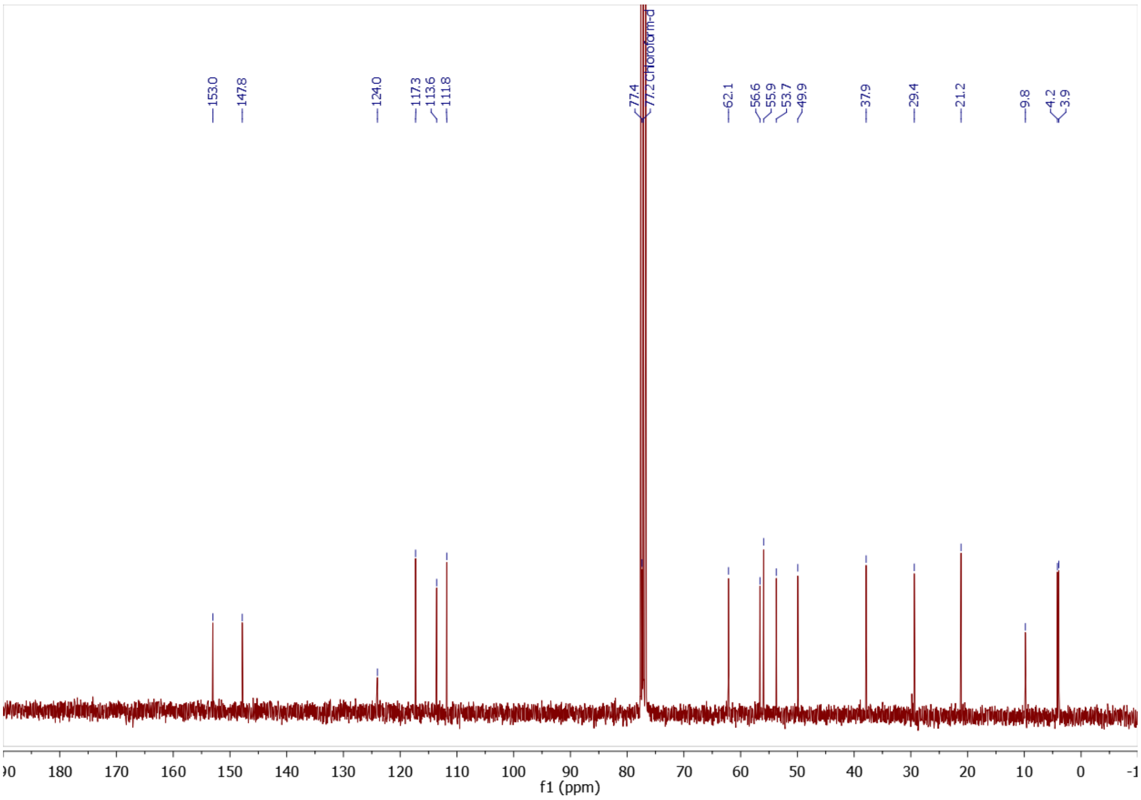
153.0
147.8
124.0
117.3
113.6
111.8
77.4
77.2 Chloroform-d
62.1
56.6
55.9
53.7
49.9
37.9
29.4
21.2
9.8
4.2
3.9
f1 (ppm)

Compound **9**

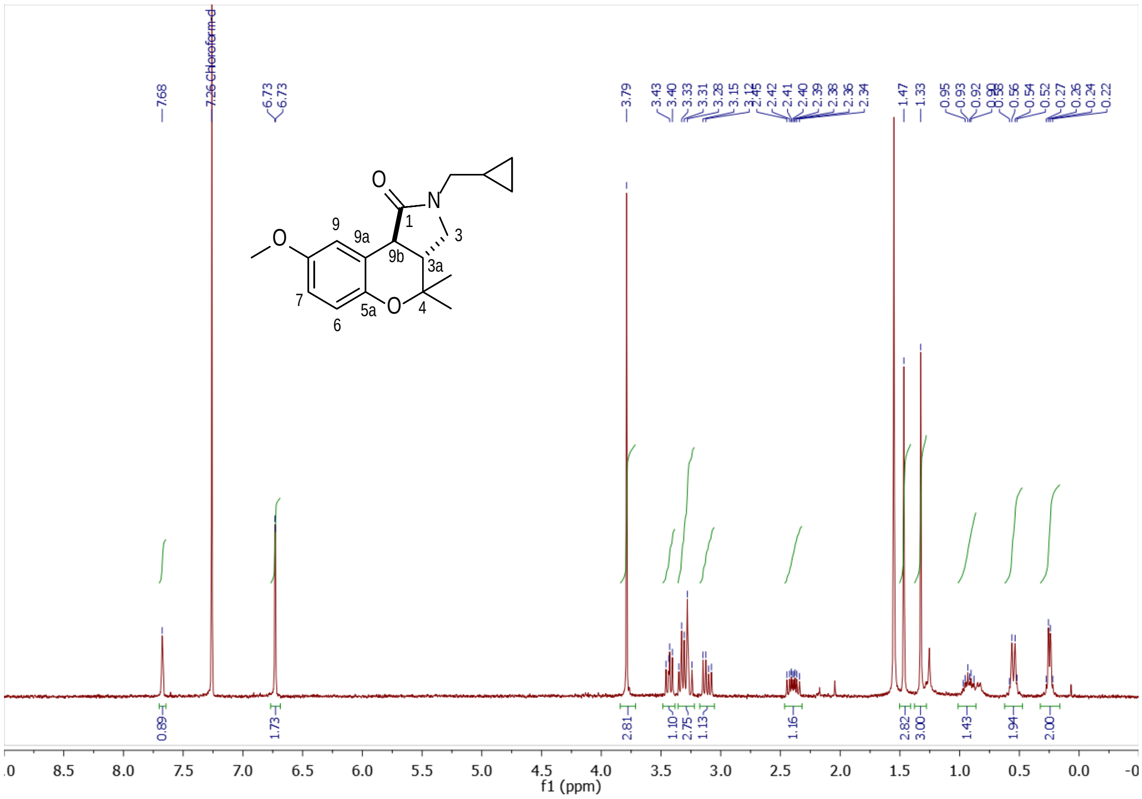

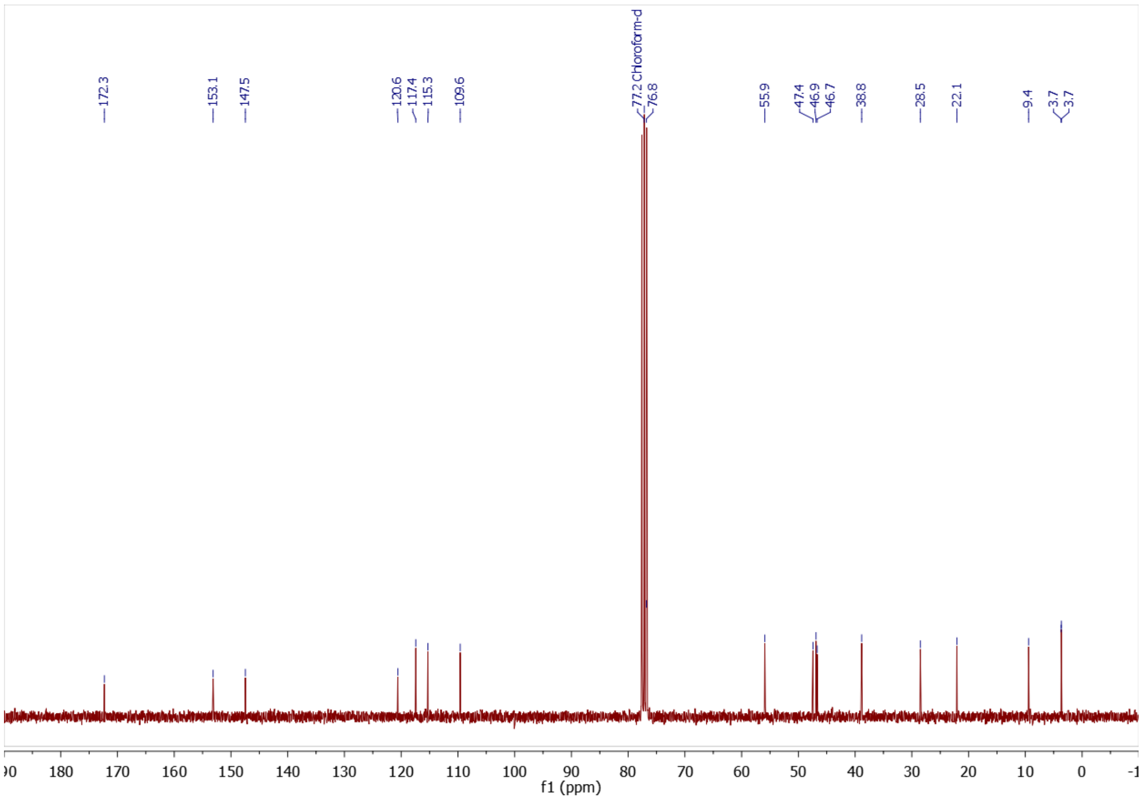

Compound **10**

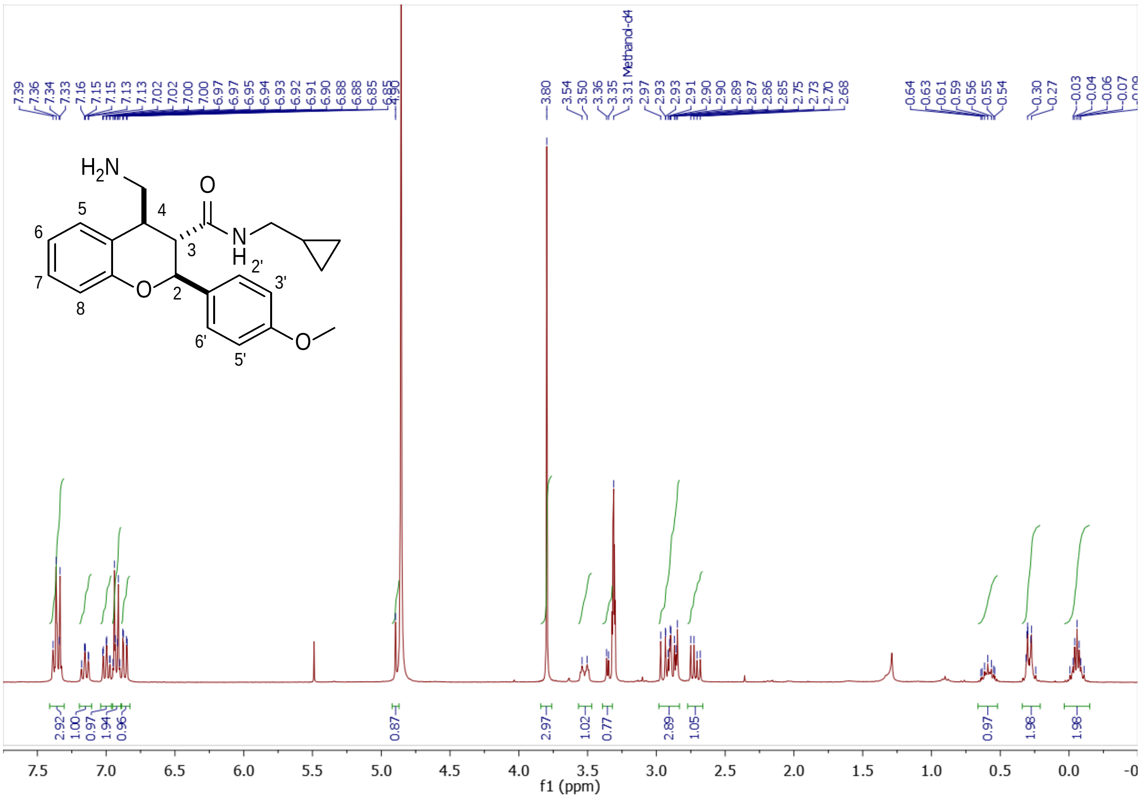

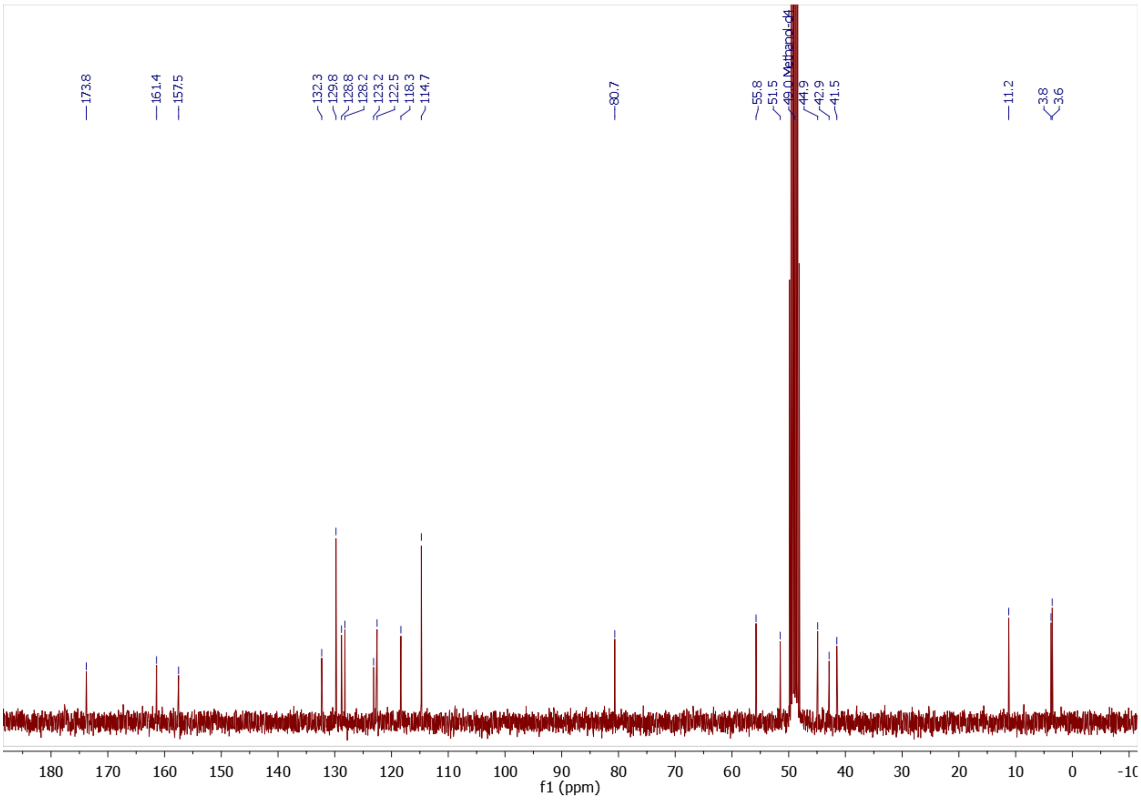

Compound **11**

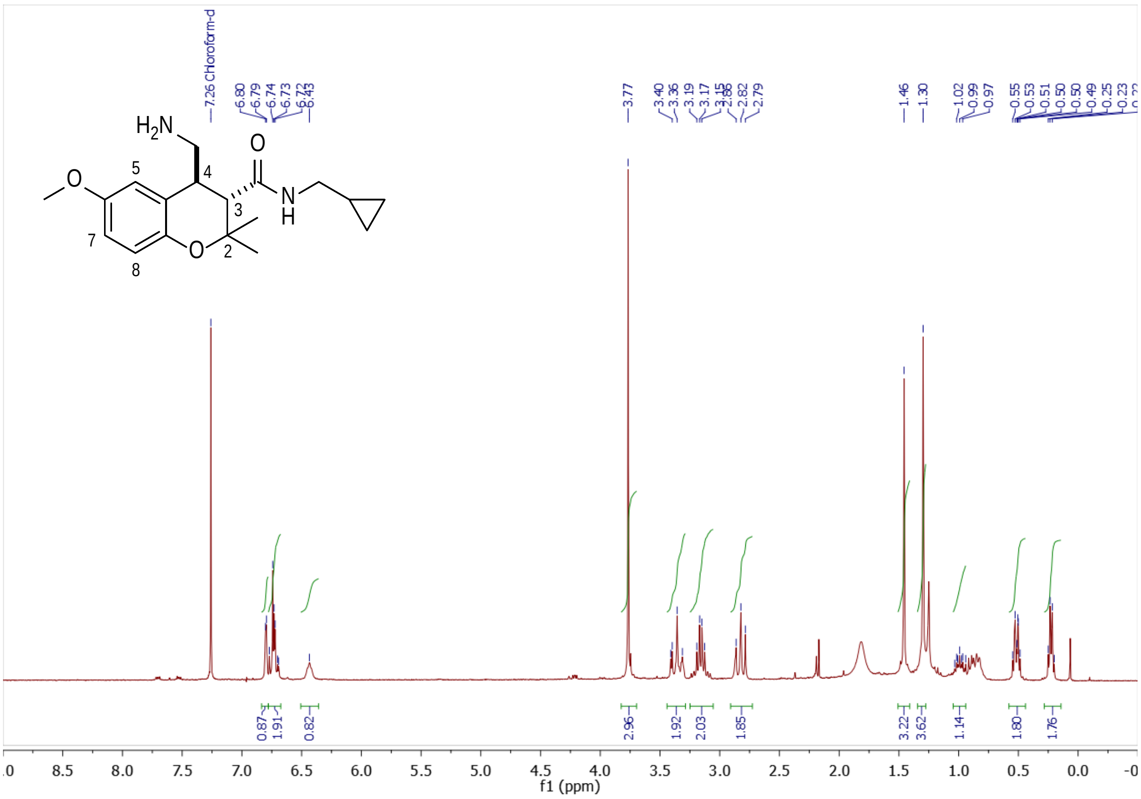

7.26 Chloroform-d
6.80
6.79
6.74
6.73
6.72
6.43
3.77
3.40
3.36
3.19
3.17
3.15
2.86
2.82
2.79
1.46
1.30
1.02
0.99
0.97
0.55
0.53
0.51
0.50
0.50
0.49
0.25
0.23
0.87
1.91
0.82
2.96
1.92
2.03
1.85
3.22
3.62
1.14
1.80
1.76
f1 (ppm)

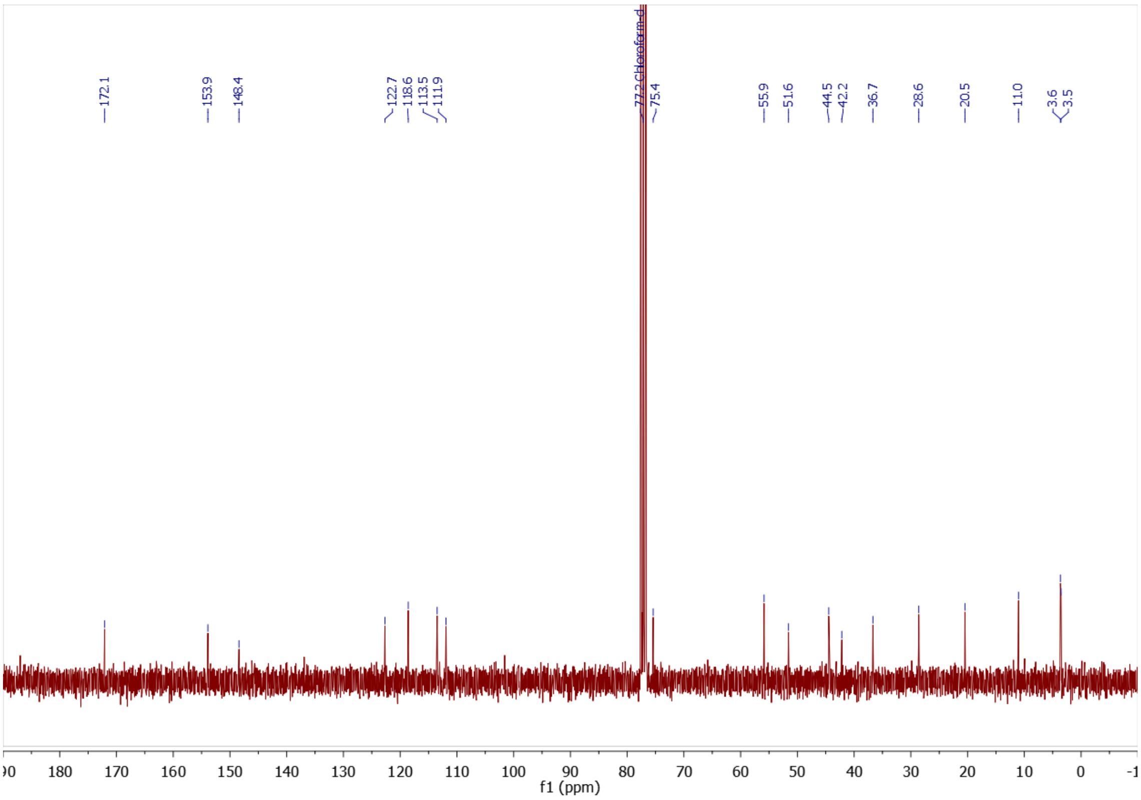

172.1
153.9
148.4
122.7
118.6
113.5
111.9
77.2 Chloroform-d
75.4
55.9
51.6
44.5
42.2
36.7
28.6
20.5
11.0
3.6
3.5
f1 (ppm)

Compound **12**

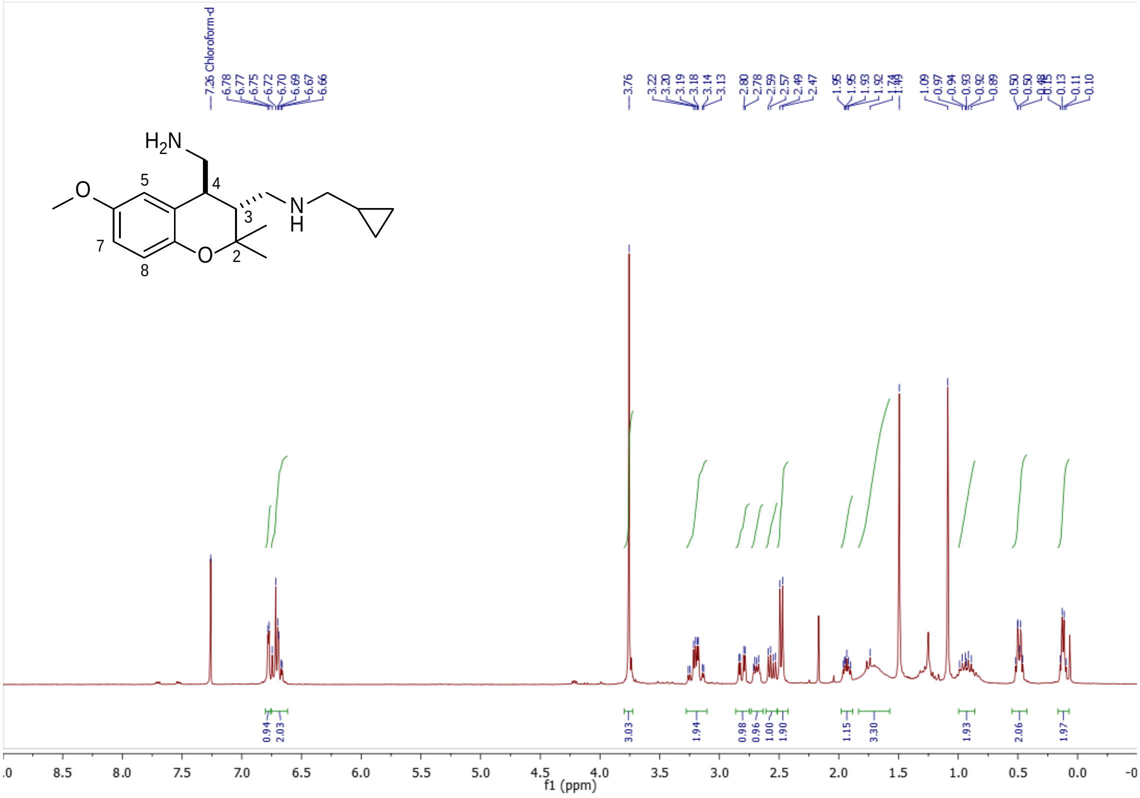

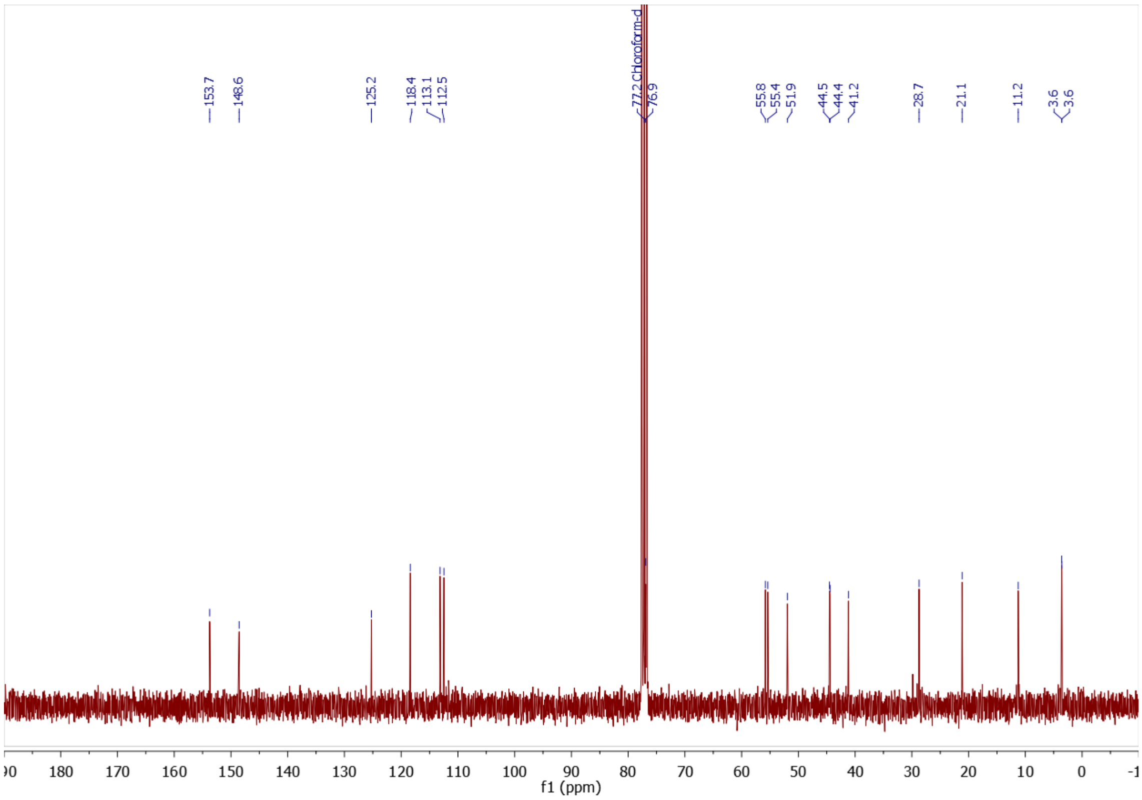

Compound **13**

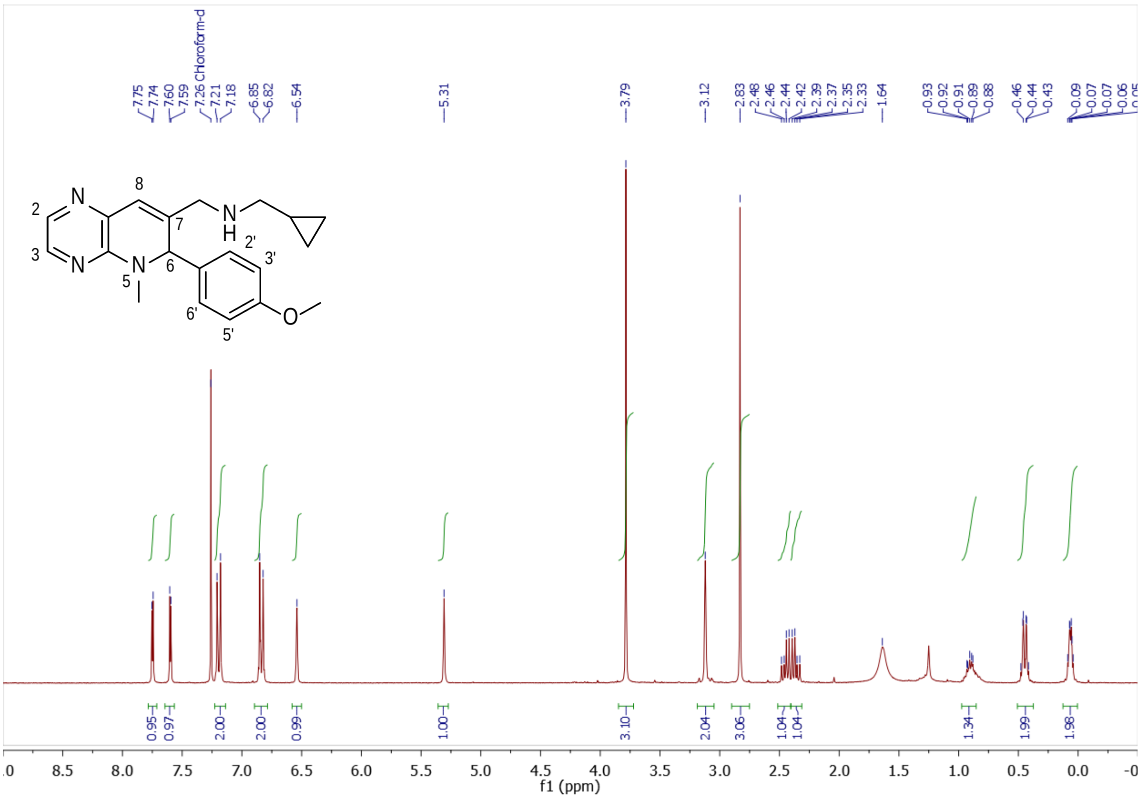

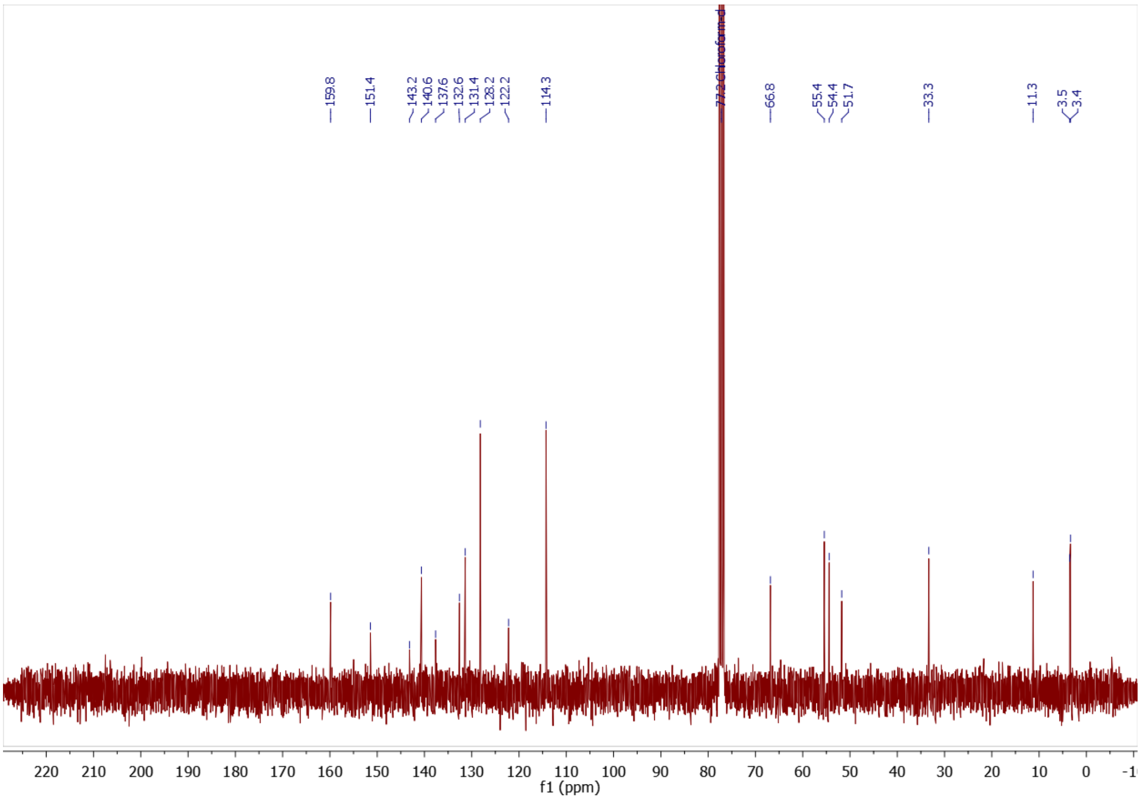

Probe **UCM-13369-Cy5**

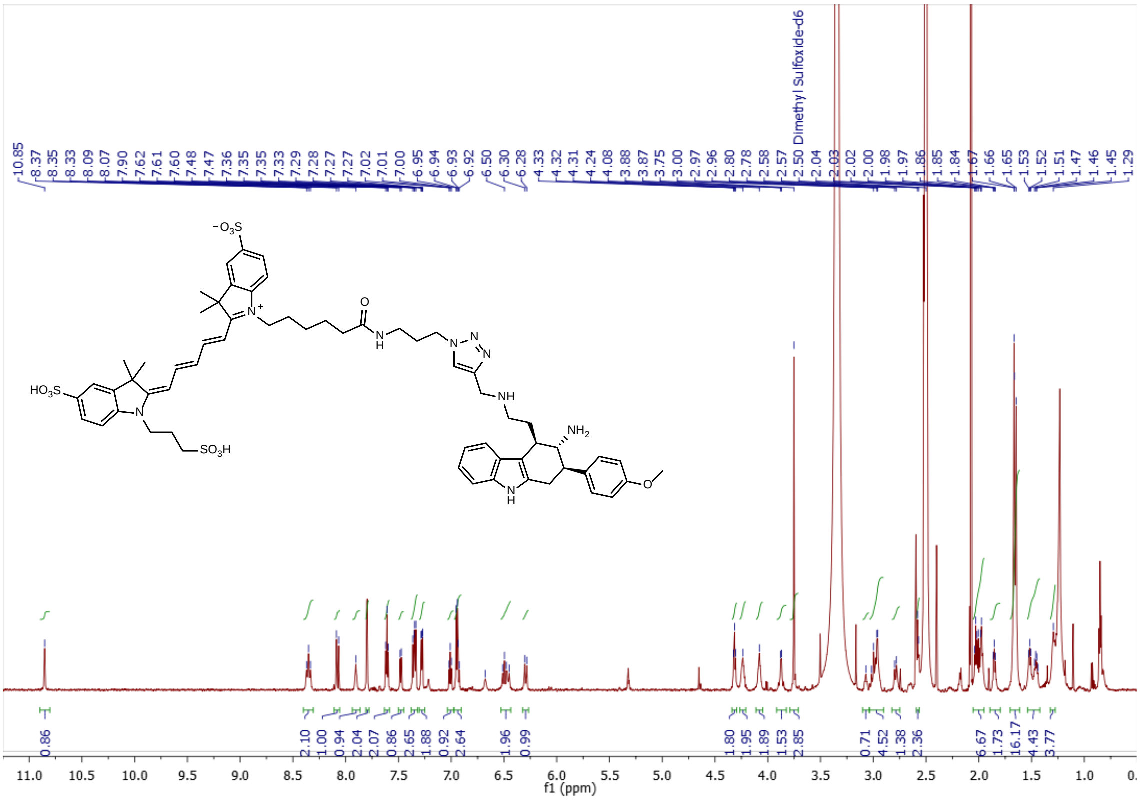

4. HPLC traces

Compound **1**

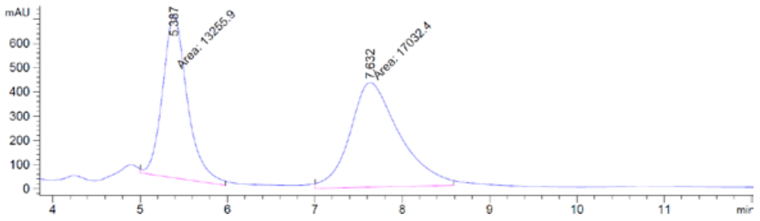

Signal 2: DAD1 C, Sig=210,8 Ref=360,100

Peak #	RetTime [min]	Type	Width [min]	Area [mAU*s]	Height [mAU]	Area %
1	5.387	MM	0.3278	1.32559e4	673.91461	43.7657
2	7.632	MM	0.6566	1.70324e4	432.33118	56.2343
Totals :				3.02882e4	1106.24579	

(4*S*,5*S*)-**1 (1a)**

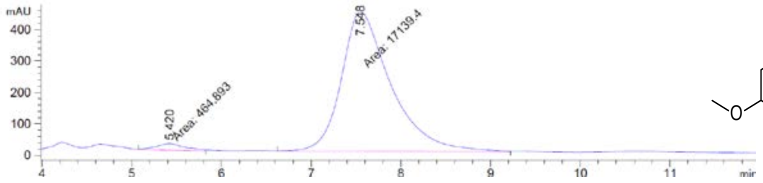

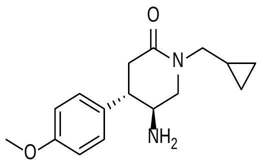

1a

Signal 2: DAD1 C, Sig=210,8 Ref=360,100

Peak #	RetTime [min]	Type	Width [min]	Area [mAU*s]	Height [mAU]	Area %
1	5.420	MM	0.3727	464.89279	20.79115	2.6408
2	7.548	MM	0.6435	1.71394e4	443.87711	97.3592
Totals :				1.76043e4	464.66825	

(4*R*,5*R*)-**1 (1b)**

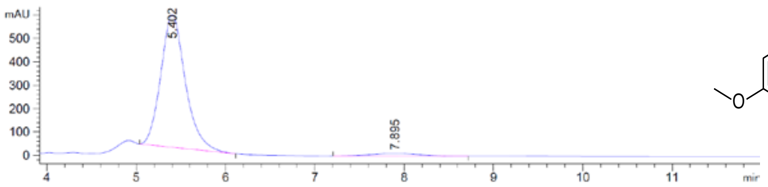

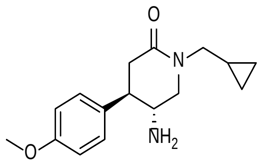

1b

Signal 2: DAD1 C, Sig=210,8 Ref=360,100

Peak #	RetTime [min]	Type	Width [min]	Area [mAU*s]	Height [mAU]	Area %
1	5.402	BB	0.2881	1.08097e4	567.87463	96.4931
2	7.895	BB	0.4609	392.86212	10.94185	3.5069
Totals :				1.12026e4	578.81648	

Compound **2**

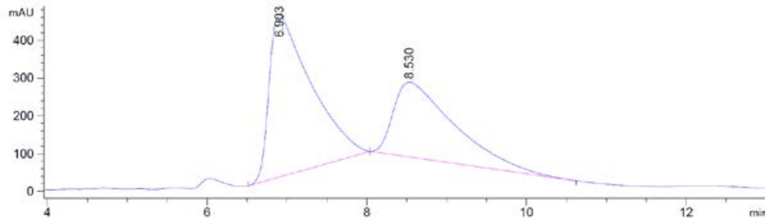

Signal 2: DAD1 C, Sig=210,8 Ref=360,100

Peak #	RetTime [min]	Type	Width [min]	Area [mAU*s]	Height [mAU]	Area %
1	6.903	BB	0.4949	1.56275e4	429.07260	59.6219
2	8.530	BB	0.7351	1.05835e4	197.51636	40.3781
Totals :				2.62110e4	626.58896	

(3*S*,4*S*)-**2** (**2a**)

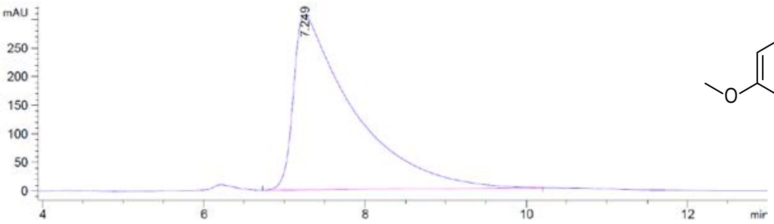

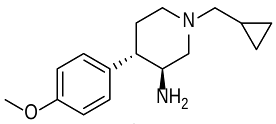

2a

Signal 2: DAD1 C, Sig=210,8 Ref=360,100

Peak #	RetTime [min]	Type	Width [min]	Area [mAU*s]	Height [mAU]	Area %
1	7.249	BB	0.6887	1.57614e4	306.09824	100.0000
Totals :				1.57614e4	306.09824	

(3*R*,4*R*)-**2** (**2b**)

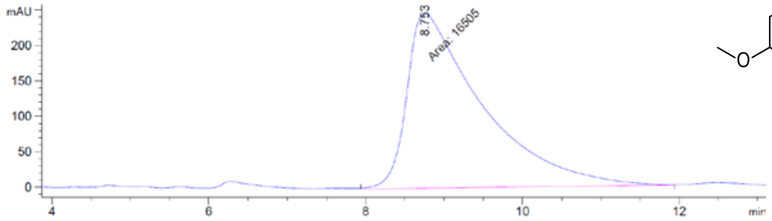

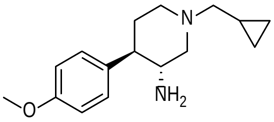

2b

Signal 2: DAD1 C, Sig=210,8 Ref=360,100

Peak #	RetTime [min]	Type	Width [min]	Area [mAU*s]	Height [mAU]	Area %
1	8.753	MM	1.1160	1.65050e4	246.49368	100.0000
Totals :				1.65050e4	246.49368	

Compound **16b** (central core precursor of compound **4b** (**UCM-13369**)):

The er was determined after reduction to the corresponding alcohol and chiral HPLC analysis:

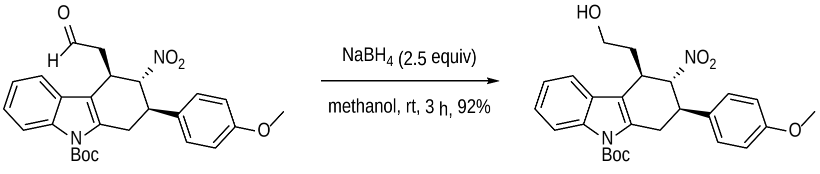

16b

***tert*-Butyl 4-(2-hydroxyethyl)-2-(4-methoxyphenyl)-3-nitro-1,2,3,4-tetrahydro-9*H*-carbazole-9-carboxylate:** ^{1}H-NMR (300 MHz, $CDCl_3$): δ 1.64 (s, 9H, $3CH_3$), 2.15-2.25 (m, 1H, ½C$\underline{H}_2$$CH_2$OH), 2.31-2.41 (m, 1H, ½C$\underline{H}_2$$CH_2$OH), 3.19-3.31 (m, 1H, H_1), 3.42-3.56 (m, 2H, H_1, H_2), 3.59-3.72 (m, 2H, CH_2C$\underline{H}_2$OH), 3.79 (s, 3H, OCH_3), 3.93-3.98 (m, 1H, H_4), 5.37 (dd, J = 11.3, 9.3, 1H, H_3), 6.87 (d, J = 8.7, 2H, $H_{3'}$, $H_{5'}$), 7.21 (d, J = 8.7, 2H, $H_{2'}$, $H_{6'}$), 7.21-7.34 (m, 2H, H_6, H_7), 7.55 (dd, J = 7.6, 0.8, 1H, H_5), 8.14 (dd, J = 7.6, 0.9, 1H, H_8).

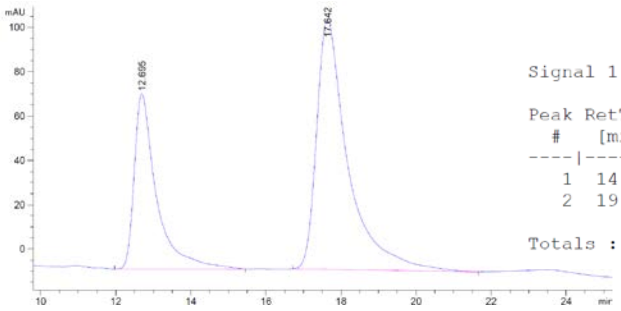

Signal 1: DAD1 A, Sig=310,4 Ref=800,230

Peak #	RetTime [min]	Type	Width [min]	Area [mAU*s]	Height [mAU]	Area %
1	14.265	MM	0.5213	126.42560	4.04190	54.8409
2	19.970	MM	0.7880	104.10609	2.20192	45.1591
Totals :				230.53168	6.24382	

***tert*-Butyl (2*S*,3*S*,4*S*)-4-(2-hydroxyethyl)-2-(4-methoxyphenyl)-3-nitro-1,2,3,4-tetrahydro-9*H*-carbazole-9-carboxylate** (obtained from **16a** intermediate)

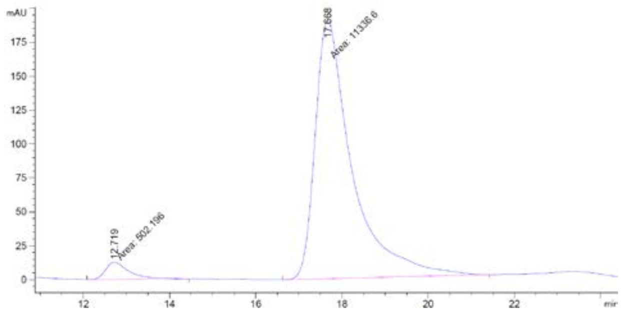

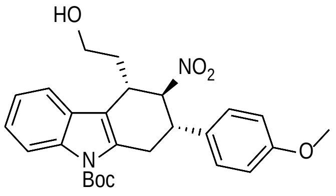

Signal 1: DAD1 A, Sig=250,4 Ref=800,100

Peak #	RetTime [min]	Type	Width [min]	Area [mAU*s]	Height [mAU]	Area %
1	12.719	MM	0.6568	502.19580	12.74315	4.2419
2	17.668	MM	0.9926	1.13366e4	190.35667	95.7581
Totals :				1.18388e4	203.09983	

tert-Butyl (2S,3S,4S)-4-(2-hydroxyethyl)-2-(4-methoxyphenyl)-3-nitro-1,2,3,4-tetrahydro-9H-carbazole-9-carboxylate (obtained from **16b** intermediate)

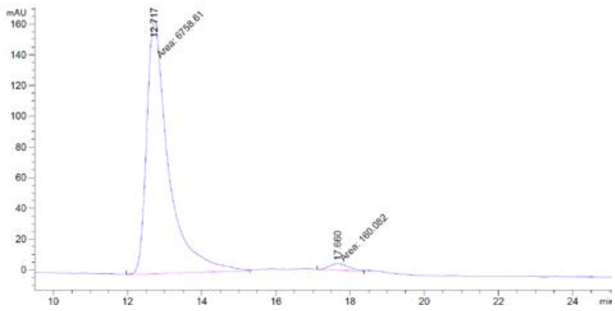

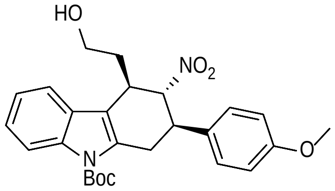

Signal 1: DAD1 A, Sig=250,4 Ref=800,100

Peak #	RetTime [min]	Type	Width [min]	Area [mAU*s]	Height [mAU]	Area %
1	12.717	MM	0.6823	6758.60938	165.08936	97.6862
2	17.660	MM	0.6214	160.08203	4.29393	2.3138

Totals : 6918.69141 169.38328

Compound **4**:

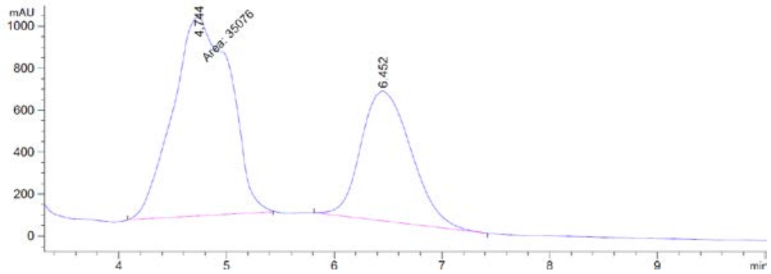

Signal 3: DAD1 D, Sig=230,16 Ref=360,100

Peak #	RetTime [min]	Type	Width [min]	Area [mAU*s]	Height [mAU]	Area %
1	4.744	MM	0.6171	3.50760e4	947.29657	63.5558
2	6.452	BB	0.4000	2.01133e4	617.43408	36.4442
Totals :				5.51893e4	1564.73065	

(2*S*,3*S*,4*S*)-**4**

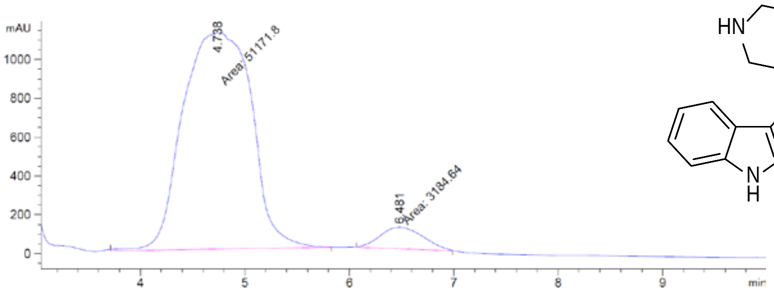

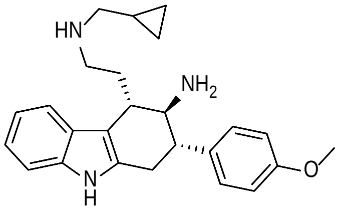

4a

Signal 3: DAD1 D, Sig=230,16 Ref=360,100

Peak #	RetTime [min]	Type	Width [min]	Area [mAU*s]	Height [mAU]	Area %
1	4.738	MM	0.7636	5.11718e4	1116.86780	94.1412
2	6.481	MM	0.4745	3184.63916	111.85084	5.8588
Totals :				5.43564e4	1228.71864	

(2*R*,3*R*,4*R*)-**4** (**4b, UCM-13369**)

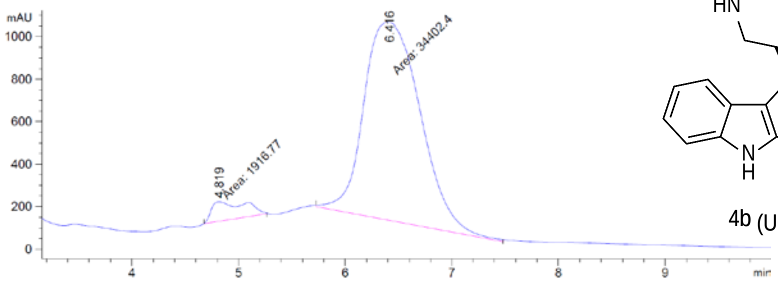

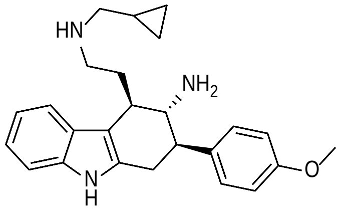

4b (UCM-13369)

Peak #	RetTime [min]	Type	Width [min]	Area [mAU*s]	Height [mAU]	Area %
1	4.819	MM	0.3474	1916.76611	91.94691	5.2776
2	6.416	MM	0.6152	3.44024e4	931.93939	94.7224
Totals :				3.63192e4	1023.88630	

Compound **5**

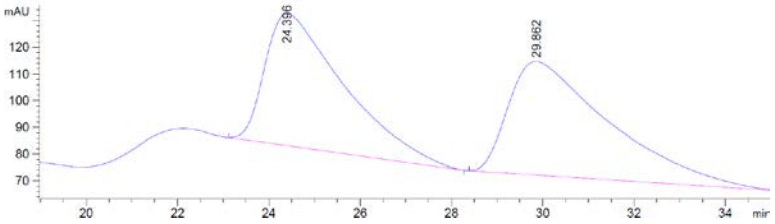

Signal 2: DAD1 C, Sig=210,8 Ref=360,100

Peak #	RetTime [min]	Type	Width [min]	Area [mAU*s]	Height [mAU]	Area %
1	24.396	BB	1.6638	5943.87695	49.39870	47.7076
2	29.862	BBA	2.0457	6515.09473	42.56524	52.2924
Totals :				1.24590e4	91.96394	

(6*S*,6a*S*,9*R*,10*R*,10a*S*)-**5** (**5a**)

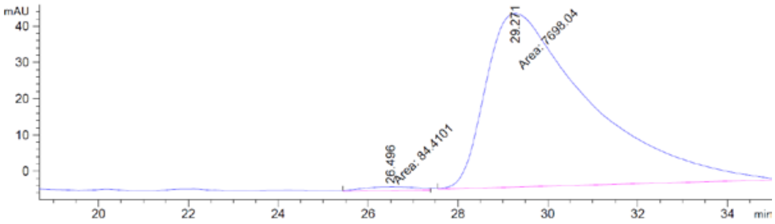

Signal 2: DAD1 C, Sig=210,8 Ref=360,100

Peak #	RetTime [min]	Type	Width [min]	Area [mAU*s]	Height [mAU]	Area %
1	26.496	MM	1.4229	84.41015	9.88676e-1	1.0921
2	29.271	MM	2.6723	7644.99414	47.67975	98.9079
Totals :				7729.40429	48.66842	

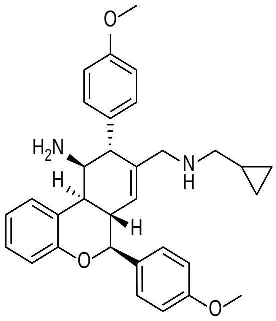

5a

(6*R*,6a*R*,9*S*,10*S*,10a*R*)-**5** (**5b**)

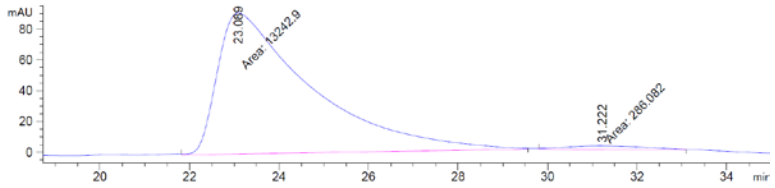

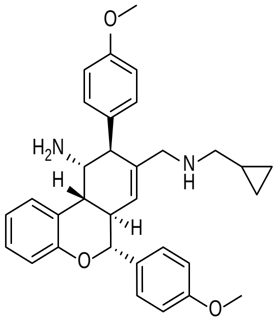

5b

Signal 2: DAD1 C, Sig=210,8 Ref=360,100

Peak #	RetTime [min]	Type	Width [min]	Area [mAU*s]	Height [mAU]	Area %
1	23.089	MM	2.4102	1.32429e4	91.57568	97.8854
2	31.222	MM	2.0909	286.08182	2.28034	2.1146
Totals :				1.35290e4	93.85603	

Compound **6**:

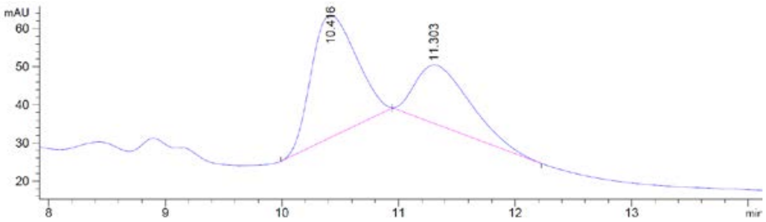

Signal 3: DAD1 D, Sig=230,16 Ref=360,100

Peak #	RetTime [min]	Type	Width [min]	Area [mAU*s]	Height [mAU]	Area %
1	10.416	BB	0.4218	855.49292	32.21457	62.6954
2	11.303	BB	0.5096	509.03079	15.32021	37.3046
Totals :				1364.52371	47.53478	

(6a*S*,9*R*,10*R*,10a*S*)-**6** (**6a**)

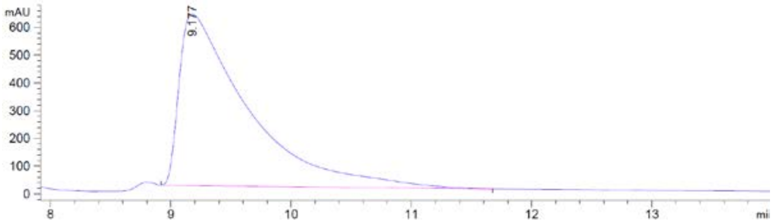

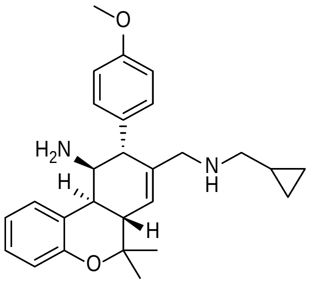

6a

Signal 3: DAD1 D, Sig=230,16 Ref=360,100

Peak #	RetTime [min]	Type	Width [min]	Area [mAU*s]	Height [mAU]	Area %
1	9.177	BB	0.5527	2.46316e4	618.38342	100.0000
Totals :				2.46316e4	618.38342	

(6a*R*,9*S*,10*S*,10a*R*)-**6** (**6b**)

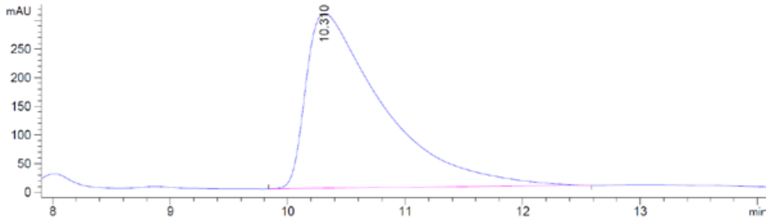

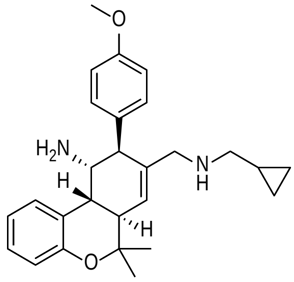

6b

Signal 3: DAD1 D, Sig=230,16 Ref=360,100

Peak #	RetTime [min]	Type	Width [min]	Area [mAU*s]	Height [mAU]	Area %
1	10.310	BB	0.6466	1.38381e4	305.39648	100.0000
Totals :				1.38381e4	305.39648	

Compound **7**:

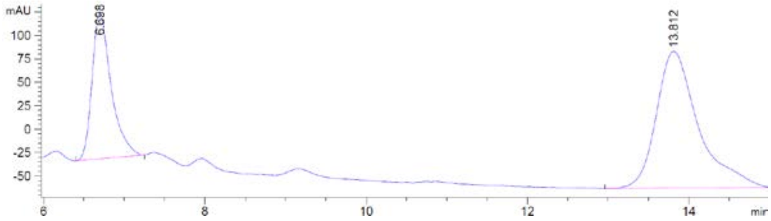


```
Signal 2: DAD1 C, Sig=210,8 Ref=360,100

Peak RetTime Type  Width     Area       Height      Area
  #    [min]         [min]   [mAU*s]     [mAU]        %
----|-------|----|-------|----------|----------|--------|
   1    6.698 BB     0.2435 2552.05786  155.46359  32.6397
   2   13.812 BBA    0.5097 5266.81201  145.69586  67.3603

Totals :                    7818.86987  301.15945
```

(3a*R*,9b*S*)-**7** (**7a**)

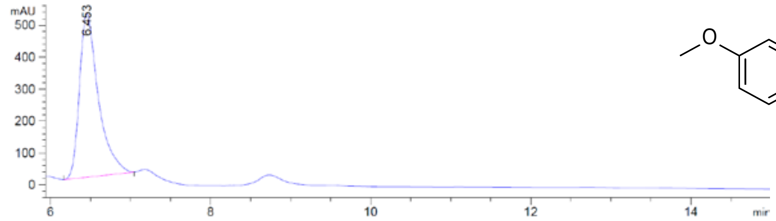

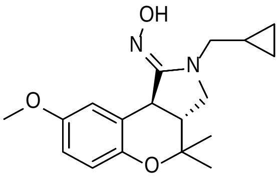

7a

```
Signal 2: DAD1 C, Sig=210,8 Ref=360,100

Peak RetTime Type  Width     Area       Height      Area
  #    [min]         [min]   [mAU*s]     [mAU]        %
----|-------|----|-------|----------|----------|--------|
   1    6.453 BB     0.2437 8433.85059  513.36176 100.0000

Totals :                    8433.85059  513.36176
```

(3a*S*,9b*R*)-**7** (**7b**)

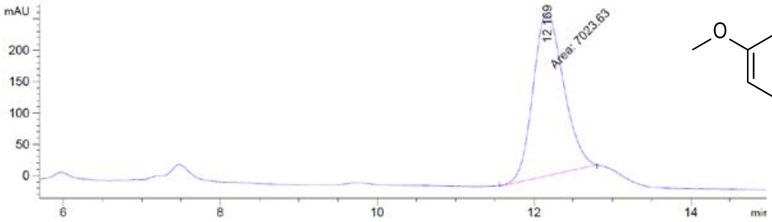

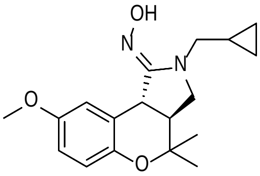

7b

```
Signal 2: DAD1 C, Sig=210,8 Ref=360,100

Peak RetTime Type  Width     Area       Height      Area
  #    [min]         [min]   [mAU*s]     [mAU]        %
----|-------|----|-------|----------|----------|--------|
   1   12.169 MM     0.4519 7023.63330  259.01773 100.0000

Totals :                    7023.63330  259.01773
```

Compound **8**:

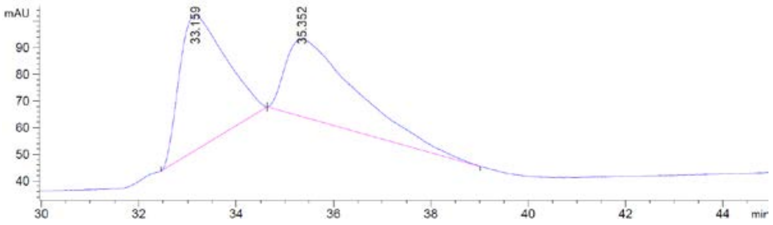

Signal 3: DAD1 D, Sig=230,16 Ref=360,100

Peak #	RetTime [min]	Type	Width [min]	Area [mAU*s]	Height [mAU]	Area %
1	33.159	BB	0.7793	3314.94678	50.79771	52.2633
2	35.352	BB	1.2269	3027.84009	29.07595	47.7367

Totals : 6342.78687 79.87365

(3a*R*,9b*S*)-**8** (**8a**)

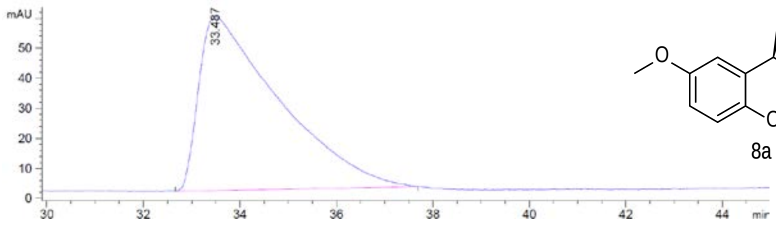

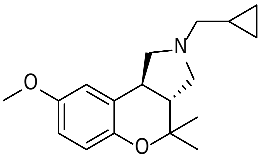

8a

Signal 3: DAD1 D, Sig=230,16 Ref=360,100

Peak #	RetTime [min]	Type	Width [min]	Area [mAU*s]	Height [mAU]	Area %
1	33.487	BB	1.3540	6693.68555	58.10400	100.0000

Totals : 6693.68555 58.10400

(3a*S*,9b*R*)-**8** (**8b**)

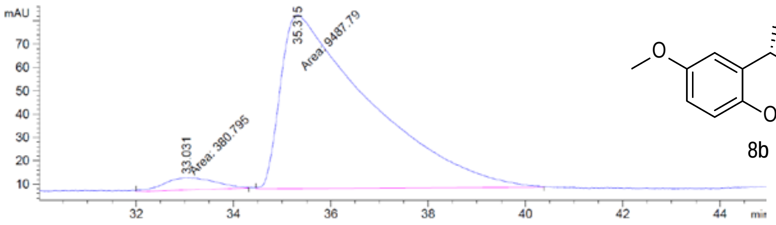

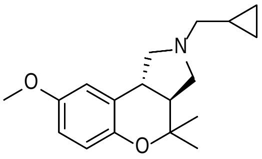

8b

Signal 3: DAD1 D, Sig=230,16 Ref=360,100

Peak #	RetTime [min]	Type	Width [min]	Area [mAU*s]	Height [mAU]	Area %
1	33.031	MM	1.2075	380.79468	5.25576	3.8587
2	35.315	MM	2.1226	9487.78516	74.49818	96.1413

Totals : 9868.57983 79.75395

Compound **10**:

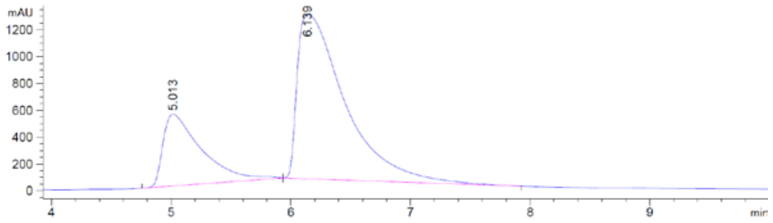

Signal 3: DAD1 D, Sig=230,16 Ref=360,100

Peak #	RetTime [min]	Type	Width [min]	Area [mAU*s]	Height [mAU]	Area %
1	5.013	BB	0.3221	1.19310e4	535.15918	25.9857
2	6.139	BB	0.4158	3.39829e4	1224.49792	74.0143
Totals :				4.59140e4	1759.65710	

(2*S*,3*S*,4*S*)-**10** (**10a**)

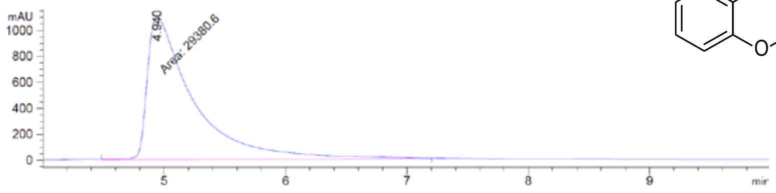

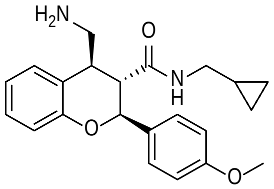

10a

Signal 3: DAD1 D, Sig=230,16 Ref=360,100

Peak #	RetTime [min]	Type	Width [min]	Area [mAU*s]	Height [mAU]	Area %
1	4.940	MM	0.4462	2.93806e4	1097.54919	100.0000
Totals :				2.93806e4	1097.54919	

(2*R*,3*R*,4*R*)-**10** (**10b**)

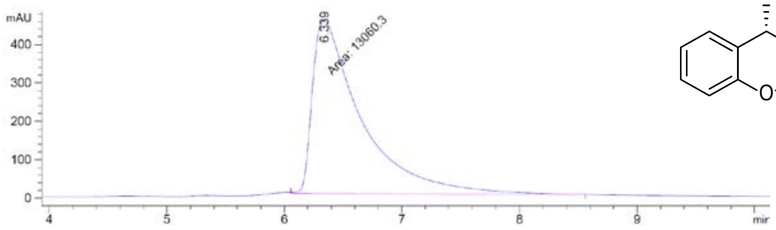

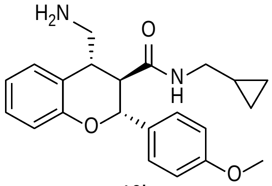

10b

Signal 3: DAD1 D, Sig=230,16 Ref=360,100

Peak #	RetTime [min]	Type	Width [min]	Area [mAU*s]	Height [mAU]	Area %
1	6.339	MM	0.4813	1.30603e4	452.24533	100.0000
Totals :				1.30603e4	452.24533	

Compound **11**:

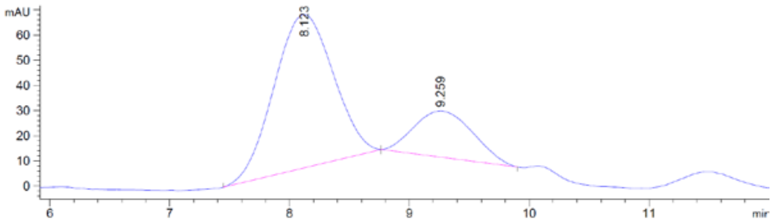

Signal 2: DAD1 C, Sig=210,8 Ref=360,100

Peak #	RetTime [min]	Type	Width [min]	Area [mAU*s]	Height [mAU]	Area %
1	8.123	BB	0.5204	2089.73120	60.89246	76.8471
2	9.259	BB	0.4349	629.60419	18.32236	23.1529

Totals : 2719.33539 79.21482

(3*S*,4*S*)-**11** (**11a**)

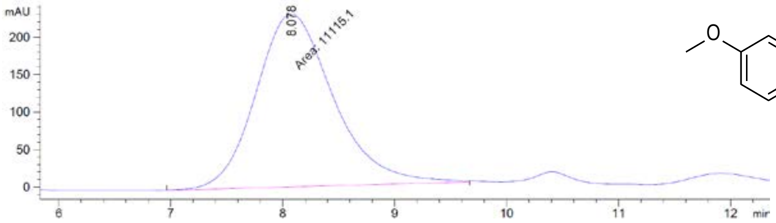

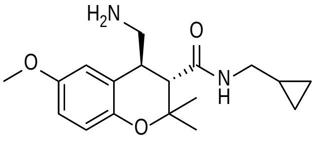

11a

Signal 2: DAD1 C, Sig=210,8 Ref=360,100

Peak #	RetTime [min]	Type	Width [min]	Area [mAU*s]	Height [mAU]	Area %
1	8.078	MM	0.8042	1.11151e4	230.35732	100.0000

Totals : 1.11151e4 230.35732

(3*R*,4*R*)-**11** (**11b**)

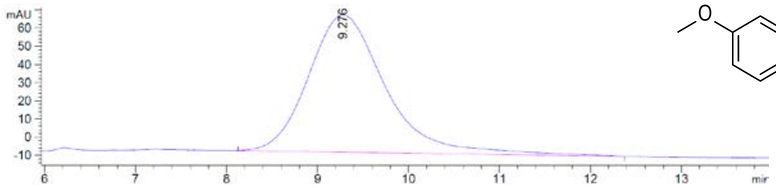

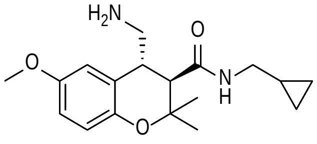

11b

Signal 2: DAD1 C, Sig=210,8 Ref=360,100

Peak #	RetTime [min]	Type	Width [min]	Area [mAU*s]	Height [mAU]	Area %
1	9.276	BB	0.8108	4452.05029	75.40199	100.0000

Totals : 4452.05029 75.40199

Compound **12**:

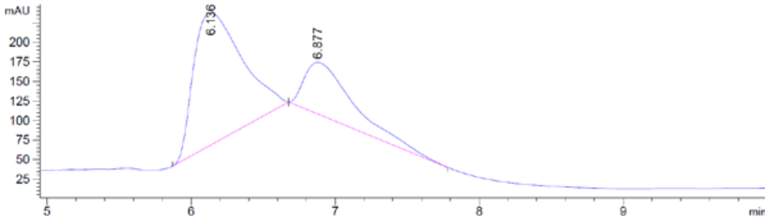

Signal 3: DAD1 D, Sig=230,16 Ref=360,100

Peak #	RetTime [min]	Type	Width [min]	Area [mAU*s]	Height [mAU]	Area %
1	6.136	BB	0.3601	3917.37036	169.19337	70.8045
2	6.877	BB	0.3597	1615.29065	65.96731	29.1955
Totals :				5532.66101	235.16068	

(3*R*,4*S*)-**12** (**12a**)

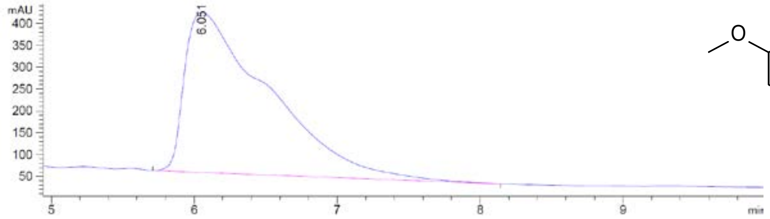

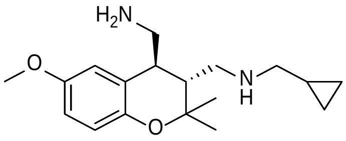

12a

Signal 3: DAD1 D, Sig=230,16 Ref=360,100

Peak #	RetTime [min]	Type	Width [min]	Area [mAU*s]	Height [mAU]	Area %
1	6.051	BB	0.5654	1.50648e4	366.59860	100.0000
Totals :				1.50648e4	366.59860	

(3*S*,4*R*)-**12** (**12b**)

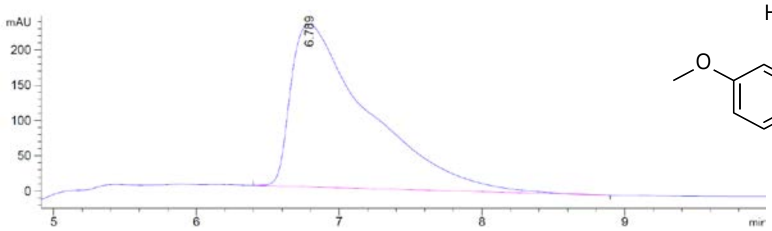

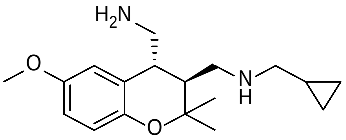

12b

Signal 3: DAD1 D, Sig=230,16 Ref=360,100

Peak #	RetTime [min]	Type	Width [min]	Area [mAU*s]	Height [mAU]	Area %
1	6.789	BB	0.5346	8749.04395	230.59550	100.0000
Totals :				8749.04395	230.59550	

5. References

(1) Huo, L. L.; Ma, A. Q.; Zhang, Y. H.; Ma, D. W. Assembly of 4-Substituted 3-Nitro-1,2,3,4-Tetrahydropyridines Via Organocatalytic Michael Addition. *Adv. Synth. Catal.* **2012**, *354*, 991-994.

(2) Kotame, P.; Hong, B. C.; Liao, J. H. Enantioselective Synthesis of the Tetrahydro-6h-Benzo[C]Chromenes Via Domino Michael-Aldol Condensation: Control of Five Stereocenters in a Quadruple-Cascade Organocatalytic Multi-Component Reaction. *Tetrahedron Lett.* **2009**, *50*, 704-707.

(3) Hong, B. C.; Kotame, P.; Tsai, C. W.; Liao, J. H. Enantioselective Total Synthesis of (+)-Conicol Via Cascade Three-Component Organocatalysis. *Org. Lett.* **2010**, *12*, 776-779.

(4) Gonzalez-Arzola, K.; Diaz-Quintana, A.; Bernardo-Garcia, N.; Martinez-Fabregas, J.; Rivero-Rodriguez, F.; Casado-Combreras, M. A.; Elena-Real, C. A.; Velazquez-Cruz, A.; Gil-Caballero, S.; Velazquez-Campoy, A.; Szulc, E.; Gavilan, M. P.; Ayala, I.; Arranz, R.; Rios, R. M.; Salvatella, X.; Valpuesta, J. M.; Hermoso, J. A.; De la Rosa, M. A.; Diaz-Moreno, I. Nucleus-Translocated Mitochondrial Cytochrome C Liberates Nucleophosmin-Sequestered Arf Tumor Suppressor by Changing Nucleolar Liquid-Liquid Phase Separation. *Nat. Struct. Mol. Biol.* **2022**, *29*, 1024-1036.

(5) Bradford, M. M. A Rapid and Sensitive Method for the Quantitation of Microgram Quantities of Protein Utilizing the Principle of Protein-Dye Binding. *Anal. Biochem.* **1976**, *72*, 248-254.

(6) Honorato, R. V.; Koukos, P. I.; Jimenez-Garcia, B.; Tsaregorodtsev, A.; Verlato, M.; Giachetti, A.; Rosato, A.; Bonvin, A. Structural Biology in the Clouds: The Wenmr-Eosc Ecosystem. *Front. Mol. Biosci.* **2021**, *8*, 729513.

(7) van Zundert, G. C. P.; Rodrigues, J.; Trellet, M.; Schmitz, C.; Kastritis, P. L.; Karaca, E.; Melquiond, A. S. J.; van Dijk, M.; de Vries, S. J.; Bonvin, A. The Haddock2.2 Web Server: User-Friendly Integrative Modeling of Biomolecular Complexes. *J. Mol. Biol.* **2016**, *428*, 720-725.

(8) Gallo, A.; Lo Sterzo, C.; Mori, M.; Di Matteo, A.; Bertini, I.; Banci, L.; Brunori, M.; Federici, L. Structure of Nucleophosmin DNA-Binding Domain and Analysis of Its Complex with a G-Quadruplex Sequence from the C-Myc Promoter. *J. Biol. Chem.* **2012**, *287*, 26539-26548.

(9) Riniker, S.; Landrum, G. A. Better Informed Distance Geometry: Using What We Know to Improve Conformation Generation. *J. Chem. Inf. Model.* **2015**, *55*, 2562-2574.

(10) Meng, E. C.; Goddard, T. D.; Pettersen, E. F.; Couch, G. S.; Pearson, Z. J.; Morris, J. H.; Ferrin, T. E. Ucsf Chimerax: Tools for Structure Building and Analysis. *Protein Sci.* **2023**, *32*, e4792.
